# Mechanochemistry: Unravelling the Impact of Metal Leaching in Organic Synthesis

**DOI:** 10.1002/cssc.202402547

**Published:** 2025-01-30

**Authors:** Francesco Basoccu, Pietro Caboni, Andrea Porcheddu

**Affiliations:** ^1^ Department of Chemical and Geological Sciences University of Cagliari Str. Interna Policlinico Universitario 09042 Monserrato Italy

**Keywords:** Mechanochemistry, AS200 control, Leaching, Unsymmetrical hydrazines, Thiourea dioxide (TDO)

## Abstract

Solvent‐free techniques have gained considerable attention in recent years due to their environmental advantages and potential to enable chemical reactivities beyond the reach of traditional solution‐based methods. Mechanochemistry has emerged as a groundbreaking approach to drive sustainable chemical processes. Despite its promise, some challenges still need to be explored, including the overlooked issue of material leaching during grinding, a phenomenon in which components from milling media or reaction vessels, such as stainless steel, unintentionally alter reaction outcomes. This study investigates the role of metal leaching in reducing arylnitrosamines by using a poorly soluble solid reagent, thiourea dioxide (TDO), focusing on stainless steel vessels. By comparing conventional mechanochemical methods with innovative solvent‐free vibratory techniques, we assess the extent of metal contamination and its impact on reaction efficiency. These findings provide new insights into how material leaching influences chemical processes and offer valuable guidance for optimizing these forward‐looking and green methodologies.

## Graphical abstract

The analysis of metal leaching from jar walls remains an overlooked aspect during the development of a mechanochemical reaction. This comprehensive study reveals the processes of metal erosion and the formation of rusty particles under intense grinding conditions, alongside their significant impact on chemical reaction efficiency, selectivity, and outcome.

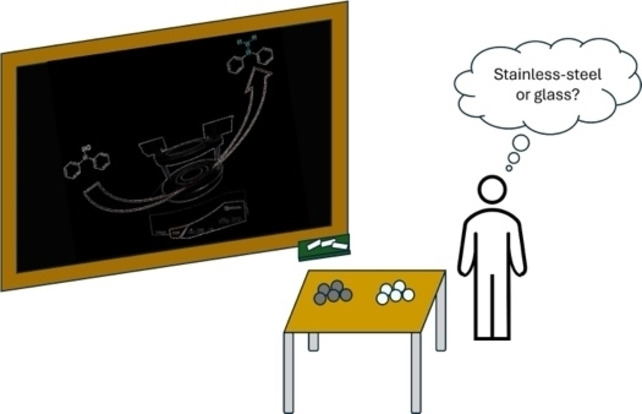

## Introduction

Over the past decade, mechanochemistry has become a highly effective technique for conducting solvent‐free reactions across various chemical disciplines.^[1]^ The widespread adoption of ball milling is primarily due to its exceptional versatility and simplicity, as extensively documented in the literature.^[13]^ This growing interest has spurred the development of diverse ball milling methodologies, resulting in various milling apparatuses constructed from different materials and engineered to produce distinct mechanical motions.^[26]^

**Figure 1:**
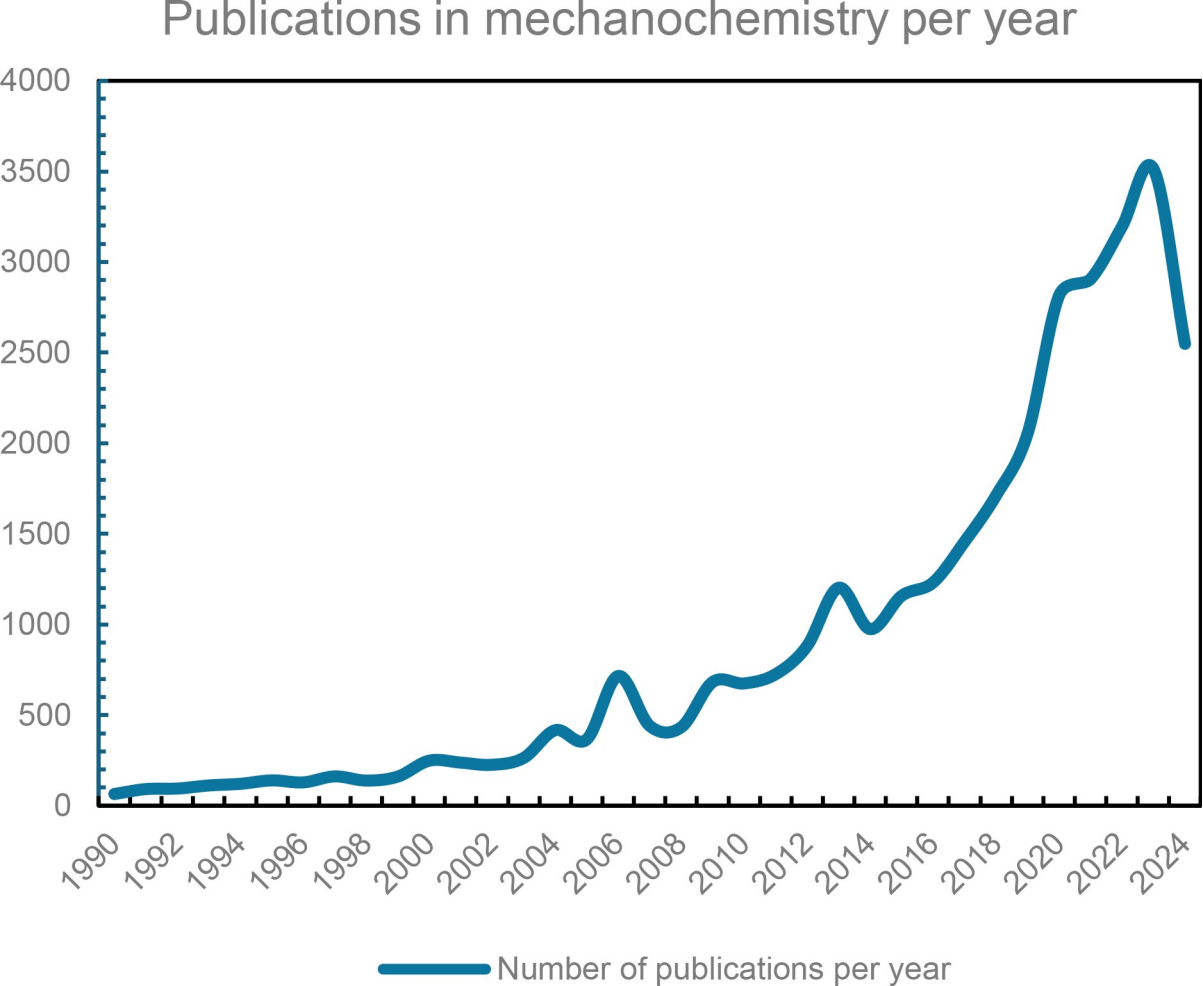
The total number of publications per year in mechanochemistry from 1990 to 2024. Source: https://app.dimensions.ai/discover/publication.

One of the most rapidly expanding applications of mechanochemistry is in organic chemistry, where its adoption has surged due to the urgent need to reduce the use of hazardous organic solvents (Figure 1).^[29]^ Moreover, it facilitates the direct formation of the target product, bypassing complex reaction conditions and laborious purification steps.[^24^, ^34^] The absence of a requirement to dissolve reactants in a solvent medium has allowed the integration of both inorganic and organic species within the same reaction system.^[35]^ This approach also provides a unique opportunity to investigate a specific solvent′s role in conventional solution‐based processes.^[36]^ Given these advantages, it might be tempting to consider mechanochemistry as the ultimate methodology for conducting various chemical processes. However, it is essential to acknowledge the drawbacks associated with this technique.^[39]^ One of the most significant issues is the challenge of reproducing specific experimental results. This difficulty arises primarily from the numerous parameters that must be carefully controlled in each mechanochemical processes include the milling frequency, the number and size of the milling balls, mechanical motion applied, and other operational variables.^[41]^ Moreover, the need for standardized procedures across different laboratories further complicates reproducibility.^[48]^

**Figure 2:**
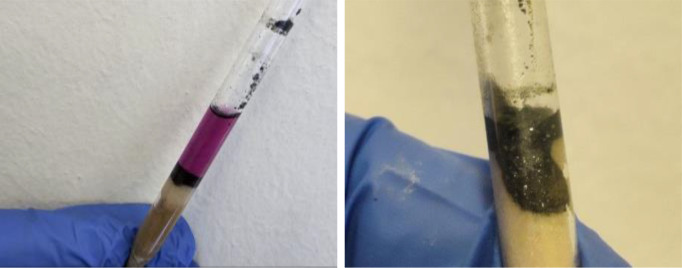
Typical filtration on celite for analyzing a crude mixture after milling in a stainless‐steel jar. The metal leaching generally blackens the reaction components. Besides, the presence of metals can be spotted even by the naked eye.

Mechanochemistry continues to evolve, so establishing uniform guidelines for experimental setups and reporting is essential to ensuring consistent and reliable outcomes and advancing the field. Among the various factors influencing mechanochemical reactions, the role of the vessel material is one of the least explored and reported aspects.^[50]^ Typically, the most common setup employs stainless‐steel jars due to their low cost and widespread availability. Consequently, numerous organic synthetic procedures utilize this approach. However, the intense grinding and crushing forces inherent in mechanochemical processes inevitably lead to wear of both the vessel and the milling balls, resulting in the leaching of metal particles into the reaction medium (Figure 2).

This unintended introduction of metal contaminants can affect reaction outcomes and product purity, underscoring the importance of carefully selecting vessel materials in mechanochemical applications.

**Figure 3:**
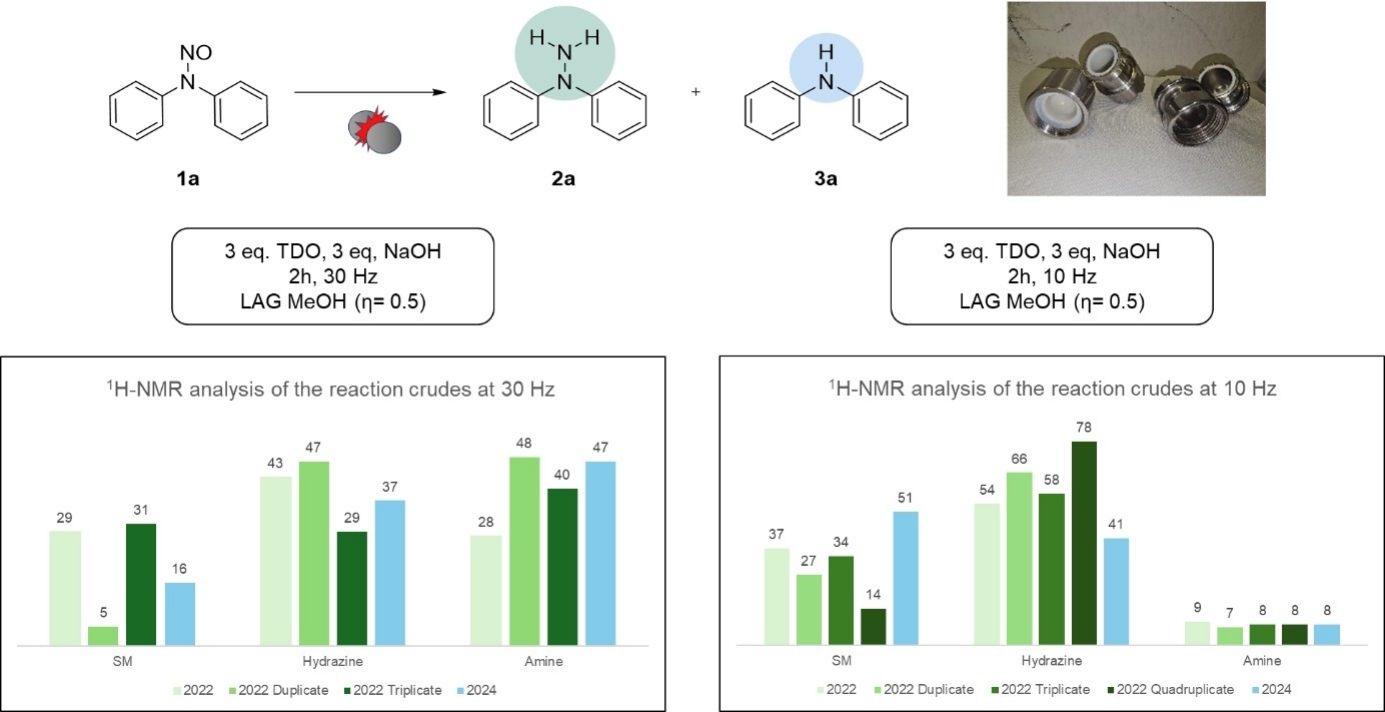
Analysis of the reproducibility of the redox process. The conversion rates depicted in the graph on the left were collected by grinding the starting material **1 a** with 3 eq. of TDO and 3 eq. of NaOH in the presence of MeOH (*η*=0.5) at 30 Hz for 2 hours with a stainless‐steel vessel equipped with 2 stainless‐steel balls (8 mm Ø, 2.0750 g per ball). The conversion rates depicted in the graph on the right were collected by grinding the starting material **1 a** with 3 eq. of TDO and 3 eq. of NaOH in the presence of MeOH (*η*=0.5) at 10 Hz for 2 hours with a stainless‐steel vessel equipped with 2 stainless‐steel balls (8 mm Ø, 2.0750 g per ball).In both cases, they were calculated through ^1^H‐NMR.

Given that metals are commonly employed as catalysts or reagents in redox processes, their unintentional introduction into mechanochemical reactions due to the wear of the milling vessels and balls can significantly influence the reactivity of the entire system.^[52]^ The unintentional presence of metal particles may alter reaction pathways, affect yields, or introduce impurities, thereby impacting the overall efficiency and selectivity of the solvent‐free technique. In this context, we analyzed a redox process conducted under solvent‐free conditions to investigate this phenomenon (Figure 3). Based on our experience with *N*‐nitroso derivatives, we proposed reducing these compounds to unsymmetrical hydrazines.[^35^, ^56^] This approach allowed us to assess the effect of metal contamination on the reaction and aims to develop a reliable method for synthesizing valuable hydrazine derivatives using mechanochemistry. The synthesis of unsymmetrical hydrazines typically relies on metal‐based reagents and requires stringent inert conditions due to their relevant instability.^[57]^ Acknowledging this, we opted to evaluate the influence of metal leaching on this redox process when performed in stainless‐steel jars, using a stoichiometric reducing agent such as thiourea dioxide (TDO).^[4]^ In this study, we report the analysis of a redox reaction conducted under solvent‐free conditions utilising both stainless‐steel and zirconia‐based milling vessels and balls. Experiments were carried out under newly proposed conditions to evaluate and, importantly, standardise the extent of metal leaching during mechanochemical grinding. To further validate our methodology, we compared the results with those obtained using a vibrational milling technique employing glass vials.

This comparative approach allowed us to assess the impact of vessel material and milling method on the reaction outcome, providing valuable insights into optimizing mechanochemical processes for sensitive redox reactions.

## Results and Discussion

We began our investigation by focusing on reducing diphenylnitrosamine (**1 a**) to synthesize diphenylhydrazine (**2 a**). In the reduction of nitroso groups, especially in aryl substrates, the formation of the corresponding amine derivative (**3 a**) as a side product is expected to be observed. Our initial experiments were conducted under mechanochemical conditions using a 10 mL stainless steel vessel with stainless steel milling balls. However, from the first trials, we encountered significant inconsistencies in the product ratio between the desired hydrazine and the denitrosated side product (Figure 3). This variability suggested that the reaction outcome was influenced by uncontrolled factors, possibly metal leaching from the stainless‐steel apparatus. Recognizing the importance of reproducibility and selectivity in our process, we decided to delve deeper into the underlying causes of this issue, which prompted further optimization and control experiments. These inconsistencies prompted us to evaluate the reaction performance in converting the starting material, as detailed in Table 1. Initially, we conducted the reaction in the presence of methanol (*η*=0.5) for 2 hours at a milling frequency of 30 Hz, achieving a 78 % conversion of the starting material (Table 1, entry 1). Unfortunately, increasing the amounts of thiourea dioxide (TDO) and sodium hydroxide (NaOH) to 4 equivalents did not improve the performance (Table 1, entry 2), suggesting that reagent concentration was not the limiting factor under these conditions. Therefore, the reaction time was increased to 180 min, permitting a complete conversion of the starting material to products **2 a** and **3 a** (Table 1, entry 3). Further screening of different hydroxides and sodium carbonate underlined how sodium hydroxide was the most suitable base for this redox process (Table 1, entries 4–6). Switching the solvent to water led to a significant drop in conversion to 14 % (Table 1, entry 7), highlighting that the solvent influences the reaction outcome.

**Table 1:** Optimization of the reduction of **1 a** under mechanochemical conditions.

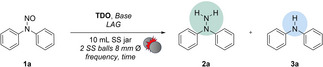				
Entry	TDO	Base (equiv)	Time	**1 a** ^[a]^ Conversion
1	3	NaOH (3)	120	78 %
2	4	NaOH (4)	120	78 %
**3**	**3**	**NaOH (3)**	**180**	100 %
4	3	LiOH (3)	180	93 %
5	3	KOH (3)	180	80 %
6	3	Na_2_CO_3_ (3)	180	20 %
7^[b]^	3	NaOH (3)	120	14 %

[a] The amount of base and TDO is expressed in terms of equivalents, the milling frequency in terms of Hertz, time in terms of minutes, and the amount of solvent in terms of *η* factor (it expresses the ratio of solvent volume to the weight of reaction mixture in mg mL^−1^). Otherwise stated, all the reactions were run in a Retsch‐MM500 Vario ball mill by using 1.00 mmol of **1 a**, TDO, NaOH, and a solvent additive (*η*=0.5) in a 10 mL stainless steel jar equipped with 2 SS balls (8 mm Ø, 2.0750 g per ball) and shaken at a frequency of 30 Hz. The conversion rate was calculated through ^1^H‐NMR. [b] Water was used as a solvent additive.

**Figure 4:**
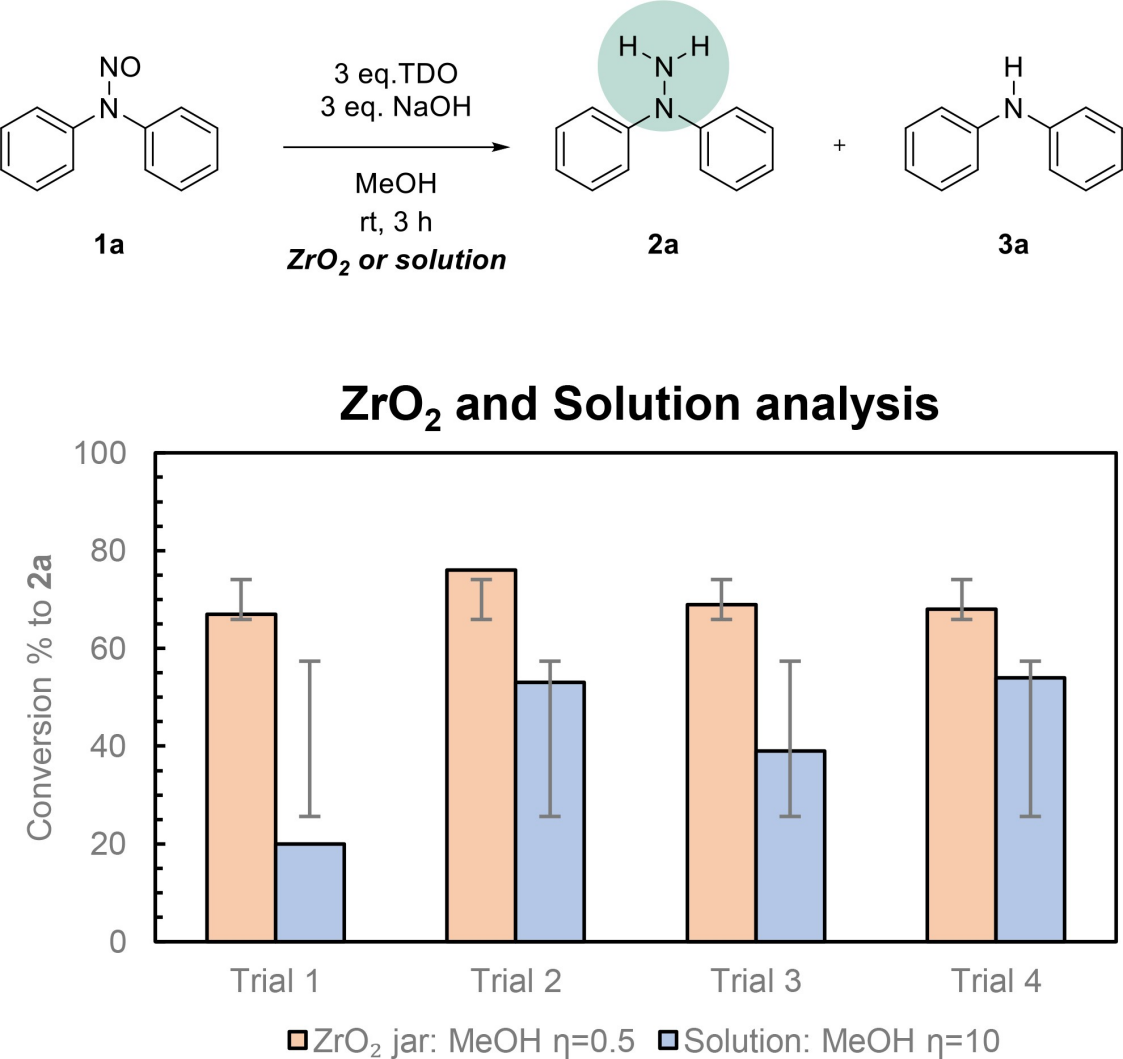
Results were collected under ball milling conditions with a zirconia apparatus and through an in‐solution approach. The conversion rate was analyzed through ^1^H‐NMR.

After finding the optimized conditions for our methodology, we began to analyze which parameters affected the ratio between products **2 a** and **3 a**. For this reason, the process was also tested with a zirconia apparatus and in solution (Figure 4). Regarding the zirconia trials, the reproducibility of the process was notably consistent compared to the experiments run with the stainless‐steel apparatus across different experiments. These results further underscore the significant role of the vessel material in mechanochemical reactions. In contrast, the solution‐based approach did not show a stable trend in several analyses, likely due to the high viscosity and heterogeneity of the reaction mixture caused by the insolubility of TDO in organic solvents, adversely affecting the mixing process.

Although it may seem reasonable to investigate the entire methodology using a zirconia apparatus, this approach has several practical drawbacks.^[39]^ Scaling up a mechanochemical process with a zirconia setup is complex and prohibitively expensive due to the high cost of the material.

Additionally, the intense crushing forces used during milling lead to vessel wear, resulting in the leaching of zirconia particles into the reaction mixture. Therefore, we need a completely different apparatus to achieve our objectives. The optimal conditions for mechanochemical processes involve using an inert material like glass to contain the reaction components.^[60]^ However, the fragility of glass makes it incompatible with the intense crushing forces typical of mechanochemistry. Similarly, attempts to employ conventional stirring techniques proved ineffective. This limitation underscored the need for a novel approach to utilize glass under solvent‐free conditions.

**Figure 5:**
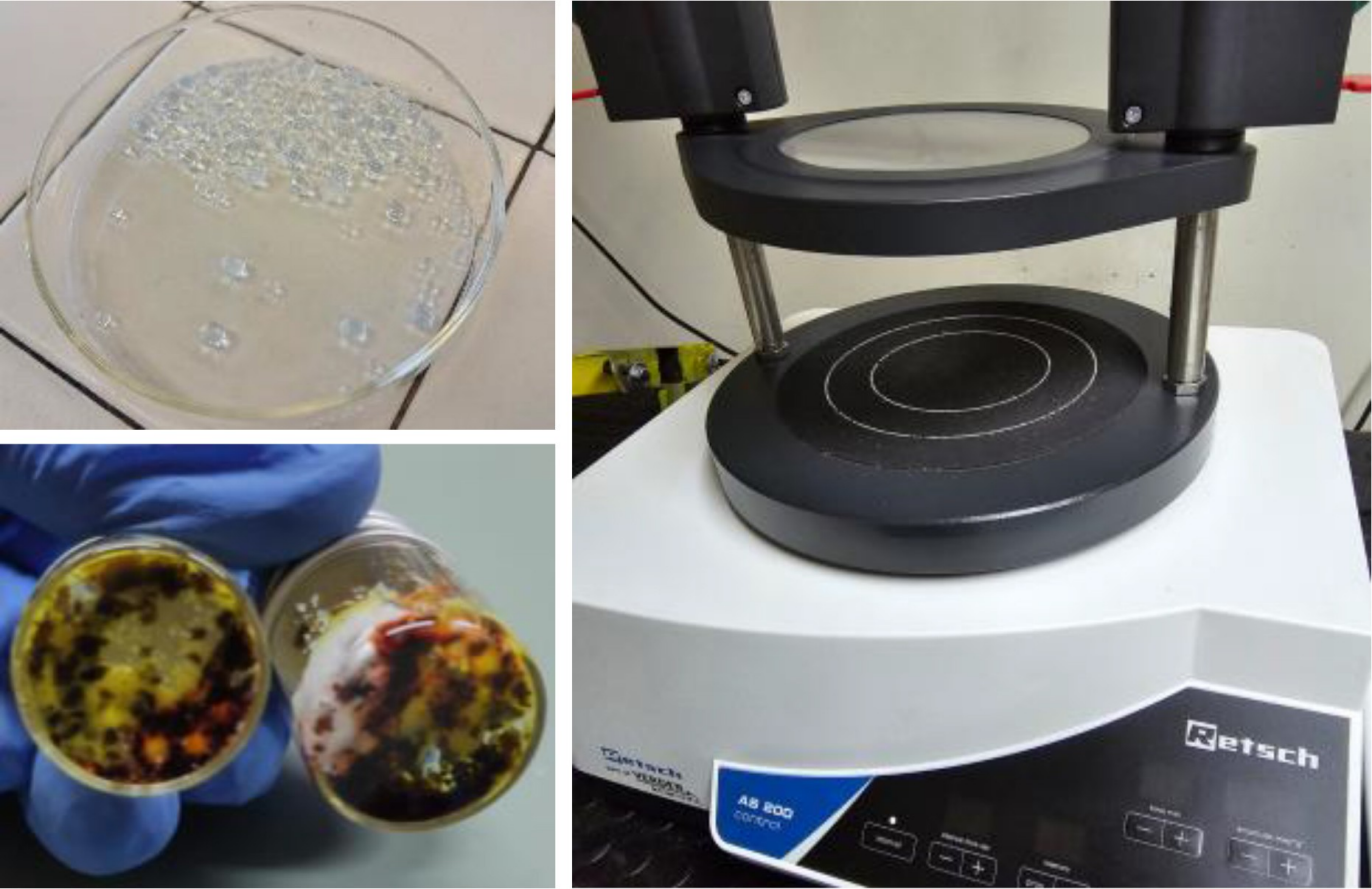
Standard instruments for running solvent‐free processes. Glass vessels and balls used for running processes in the AS200 control instrument (on the left). AS200 Control Siever developed by Retsch (on the right).

In light of recent advances in solvent‐free methodologies, we turned our attention to 3D vibrational mixing techniques as a potential solution for conducting organic reactions. These methods allow for precise mixing of bulk materials, promoting reactivity in both inorganic and organic systems under heterogeneous conditions without relying on large quantities of organic solvents. As a result, we decided to explore the reduction of *N*‐nitrosamines using an innovative vibrational instrument, precisely the Retsch AS200 Control machine (Figure 5, right image).

This instrument operates on a platform that generates three‐dimensional vibrations transmitted to the reaction mixture placed on it. The machine offers continuous operation, with the magnitude of the vibrations described by the “g” factor—a ratio of the amplitude of motion to the gravitational acceleration exerted on the mixture. This precise control of vibrational energy enhances mixing efficiency and potentially opens new possibilities for running organic reactions under solvent‐free conditions with minimal contamination. The maximum acceleration of the Retsch AS200 control machine is 3.0 mm/G, approximately 18 times the acceleration due to gravity (18 g). To make the mixing process comparable to traditional grinding methods, we loaded the glass vials used to react with glass balls (Figure 5, left images). Using an inert and inexpensive material like glass for both the reaction vessels and the milling media offers a viable alternative that is not feasible with conventional grinding approaches due to glass′s fragility under high‐impact forces.

Moreover, the ability to load multiple glass vials simultaneously onto the vibrating platform, along with the option to scale up the process to kilogram quantities, highlights the significant potential of this vibrational technique for solvent‐free applications. This scalability increases throughput and allows for the efficient synthesis of larger quantities without compromising reaction efficiency or introducing contaminants. By eliminating metal wear from milling equipment, we reduce the risk of metal leaching into the reaction mixture, enhancing product purity.

Following this line, the approach described aligns with green chemistry principles by minimizing waste and avoiding using hazardous materials. By employing this innovative vibrational method, we overcome the limitations associated with traditional grinding techniques and open new avenues for conducting mechanochemical reactions under solvent‐free conditions. This advancement holds promise for broader applications in organic synthesis, particularly for processes where product purity and scalability are crucial.

In this case, the reduction of diphenyl nitrosamine (**1 a**) to phenylhydrazine (**2 a**) was performed using a 5 mL glass vial equipped with 3 mm Ø glass balls (the ratio of the number of balls to the weight of the whole mixture was 1 glass ball to 32 mg of reacting mixture, and the weight of a single ball was 35.6 mg). The results obtained are summarised in Table 2.

**Table 2:** Optimization of the reduction of **1 a** under the AS200 conditions.

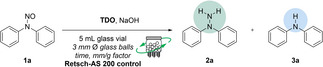				
Entry	TDO	NaOH	Time	Conversion (% **2 a**:**3 a**)^[a]^
1	3.0	3	60	53 : 11
2	3.0	3	120	79 : 8
3	3.0	3	240	83 : 9
4	4.0	4	120	85 : 11
5	4.0	8	120	53 : 47
6^[b]^	3.0	3	120	0: 3
7^[b]^	4.0	4	120	0 : 5
8^[c]^	4.0	4	120	47 : 13

[a] The amount of NaOH and TDO is expressed in terms of equivalents, the milling frequency in terms of Hertz, time in terms of minutes, and the amount of solvent in terms of η factor (it expresses the ratio of solvent volume to the weight of reaction mixture in mg mL^−1^). Otherwise stated, all the reactions were run in a Retsch‐AS200 basic by using 1.00 mmol of **1 a**, TDO, NaOH, and a solvent additive (*η*=1.0) in a 5 mL glass vial equipped with 3 mm Ø glass balls (ratio number of balls: weight of the whole mixture=1 glass ball: 32 mg of reacting mixture, the weight of 1 glass ball 3 mm Ø is 35.6 mg). The conversion rate was calculated through ^1^H‐NMR. [b] Water was used as a solvent additive. [c] The reaction was run with an amplitude of 1 mm/G.

The reactivity of compound **1 a** under vibrational mixing conditions was comparable to the results obtained in our previous mechanochemical trials (Table 2, entries 1 and 2). Increasing the reaction time to 240 minutes allowed a slight increase in the conversion rate to 83 % (Table 2, entry 3). Notably, an almost complete conversion of the starting material was achieved when 4 equivalents of thiourea dioxide (TDO) and sodium hydroxide (NaOH) were used (Table 2, entry 4). However, increasing the amount of NaOH to 8 equivalents led to more significant degradation of the target product (Table 2, entry 5), suggesting that an excess of the base may promote side reactions or decomposition pathways detrimental to the yield. As expected, water as a liquid additive did not facilitate significant conversion to the desired product (Table 2, entries 6 and 7). This outcome is likely due to the interference that water can cause in the solid‐state reaction dynamics, underscoring the importance of maintaining solvent‐free conditions for optimal reactivity. Furthermore, we examined the effect of vibrational acceleration by reducing it to 1 mm/G, which resulted in a remarkable decrease in the conversion rate (Table 2, entry 8). This observation highlights the critical role of vibrational energy input in driving the reaction forward; insufficient acceleration may not provide the necessary energy to overcome activation barriers.

**Figure 6:**
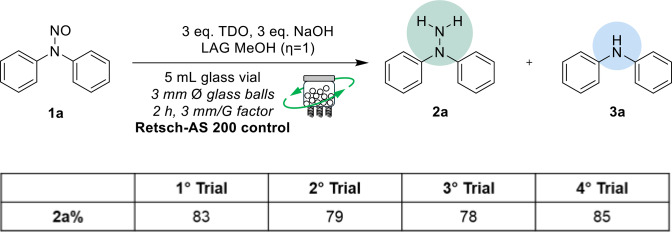
Consistency of the conversion to **2 a** with the AS200 control.

Throughout these trials, we observed a consistent trend in the ratio between the target product **2 a** and the amine by‐product **3 a**, as depicted in Figure 6. This stability suggests that the formation of the by‐product is inherent to the reaction mechanism, possibly due to partial over‐reduction or de‐nitrosation pathways that are not significantly affected by the experimental conditions tested.

These findings demonstrate that optimizing the reagent equivalents and acceleration parameters effectively allows the vibrational mixing technique to reduce *N*‐nitrosamines under solvent‐free conditions with inert glass equipment. Furthermore, the ability to conduct reactions in inert glass vials loaded with glass balls significantly advances solvent‐free methodologies. By eliminating the risk of metal leaching and enabling precise control over reaction parameters, this approach holds great promise for broader applications in organic synthesis, particularly for sensitive reactions where product purity and avoidance of metal catalysts are critical.

**Figure 7:**
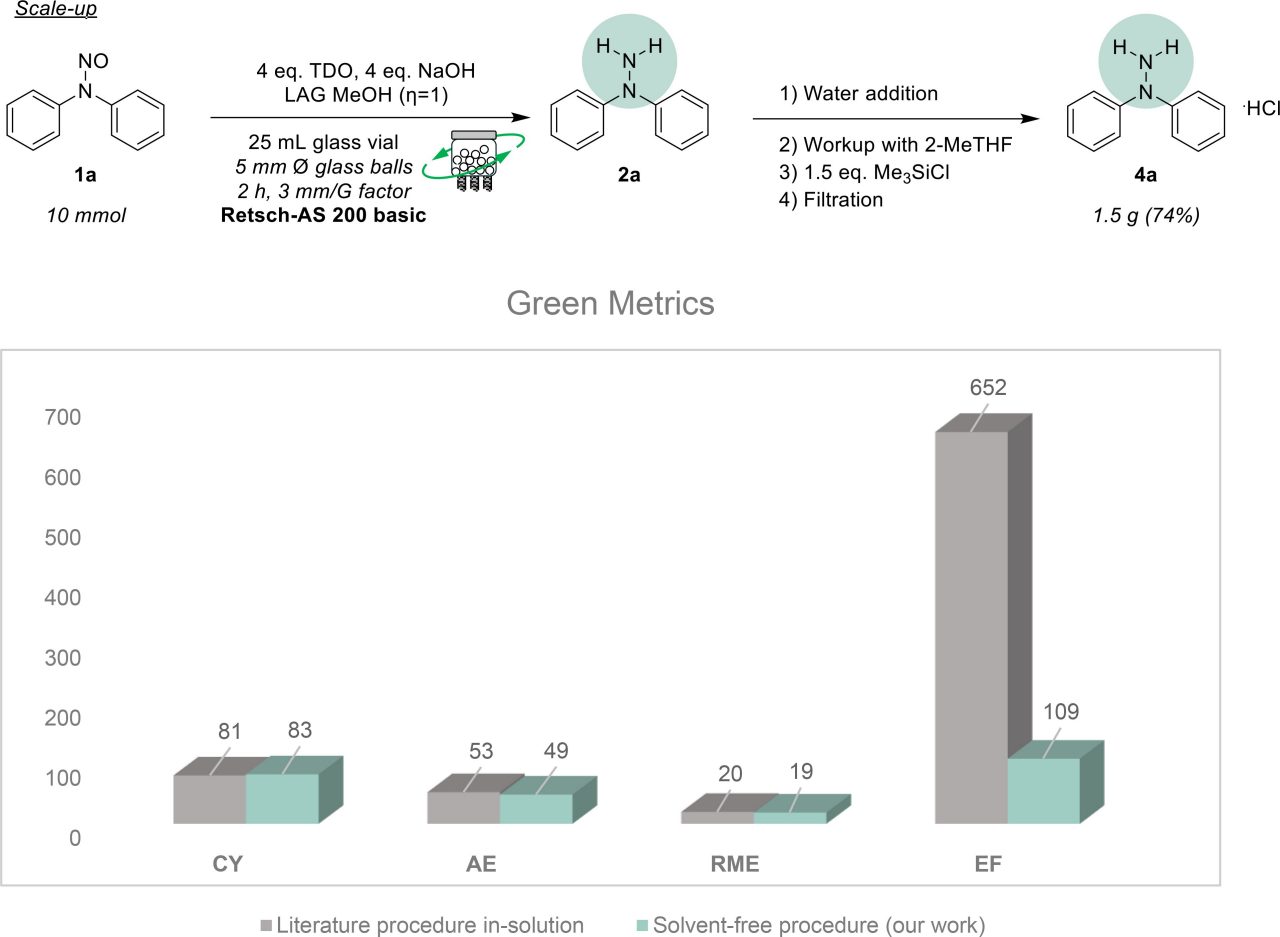
Scale‐up of the synthesis **2 a** on a 10 mmol scale (upper part). The scale‐up used a 40 mL glass vial equipped with glass balls (1 ball Ø=5 mm, ratio number of balls: weight of the whole mixture=1 glass ball: 141 mg of reacting mixture, Mass per glass ball=164 mg). Calculation and comparison of the green metrics between a reported procedure and the one described in this paper (bottom part).

To complete our assessment, we scaled up the synthesis of compound **2 a** to a 10 mmol scale, successfully recovering the pure material with a yield of 74 % (Figure 7, upper panel). This scale‐up demonstrates the practicality and potential for larger‐scale applications of our solvent‐free methodology.

We compared our entire process with a similar procedure reported in the literature to evaluate its efficacy and environmental benefits (Figure 7, lower panel). Various green chemistry metrics were analyzed, including chemical yield (CY), atom economy (AE), environmental factor (EF), and reaction mass efficiency (RME). Detailed calculations are provided in the Electronic Supplementary Information (ESI). Notably, our method achieved an exceptional EF value of 109, significantly lower than the literature value of 652. This substantial reduction highlights the greener nature of our solvent‐free procedure, indicating a decrease in waste generation and environmental impact. These findings underscore the advantages of our methodology in promoting sustainable organic synthesis practices, even on a large scale.

Building on the optimized conditions established with our new approach, we expanded the reaction scope to include various diaryl and arylalkyl nitrosamines. The proposed technique demonstrated considerable robustness with diaryl substrates, enabling the straightforward reduction of compounds **1 a–1 d** to their corresponding hydrazines in good yields. Notably, substrate **4 e**, which resembles the Diclofenac scaffold, was obtained as its hydrochloride salt with an excellent yield of 90 %. In contrast, the arylalkyl substrates **1 f–1 s** were generally isolated in yields slightly lower than those of the diaryl analogs. Among these, compound **2 i** is particularly significant, as it incorporates a portion of the antidiuretic scaffold found in Indapamide.

## Analysis of Metal Leaching

Our experimental findings revealed that the mechanochemical reduction of nitrosamines is notably affected by metal contaminants introduced into the reaction medium through abrasion from the milling jars and balls. This conclusion is supported by consistent results obtained when reactions were conducted using zirconia vessels and inert glass containers with the AS200 control vibrational mixer. Using these inert materials minimized metal leaching, resulting in more reproducible outcomes and higher purity of the target products. Accordingly, we examined how the metals released from jar surfaces and milling media influence the mechanochemical reduction process, the product distributions, and the potential trigger of side reactions.

**Figure 8:**
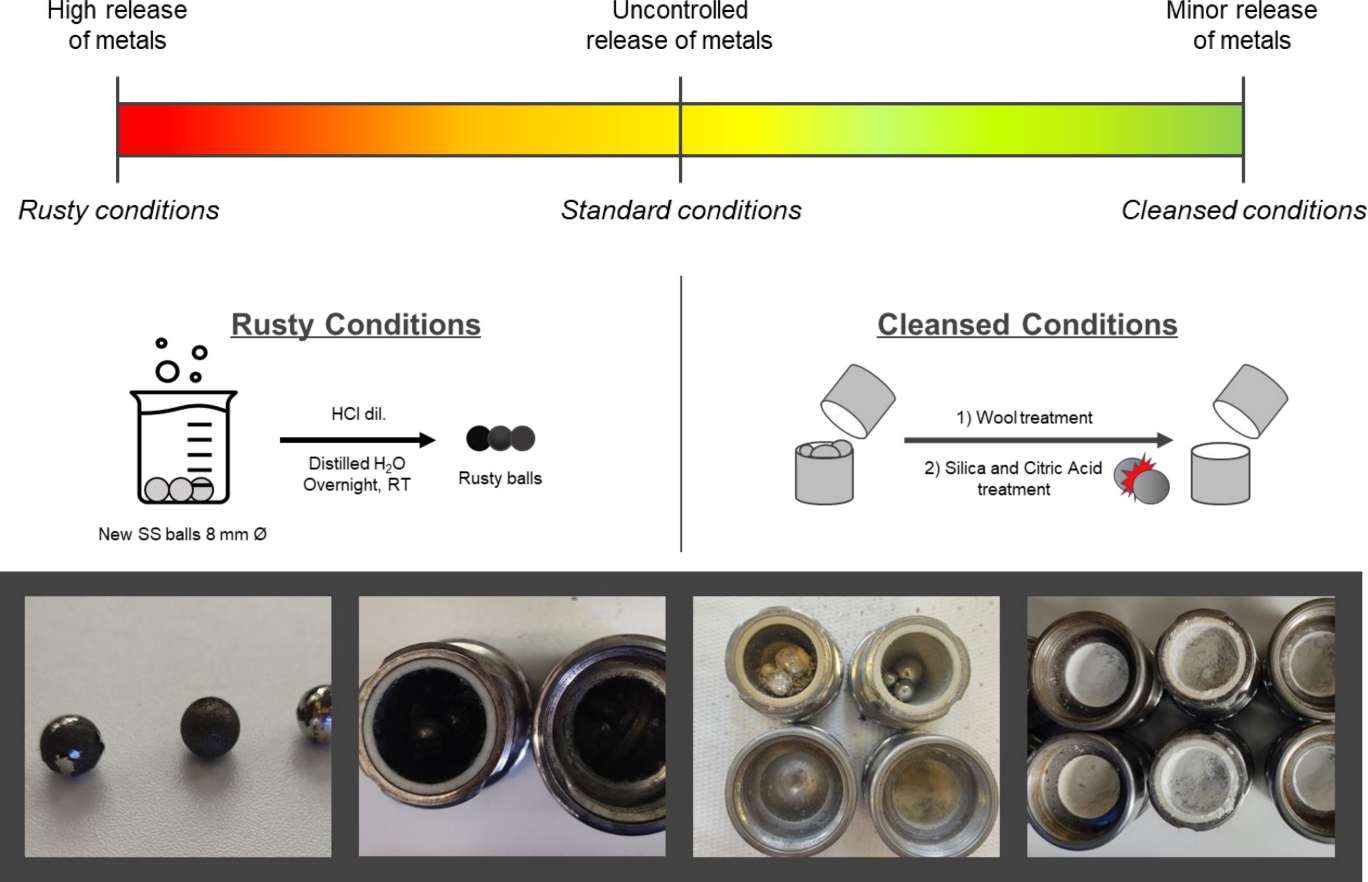
*Rusty* and *cleansed* conditions. The oxidized balls have been prepared by treating new SS balls (8 mm Ø, 2.0750 g per ball) with a diluted solution of HCl (on the left). First, the jars were cleaned with wool and then treated with citric acid and silica (on the right).

To assess the significance of metal leaching on our mechanochemical reactions, we designed two contrasting experimental conditions to standardize the data collected after the reactions (Figure 8). The first condition, termed “***rusty conditions***,” involved deliberately promoting metal release into the reaction medium using pre‐oxidized stainless‐steel balls. This intentional oxidation increased the likelihood of contaminating the reaction mixture with metal particles, simulating a worst‐case scenario for metal leaching. In contrast, the second condition aimed to minimize metal contamination and was called “**cleansed conditions**.” As demonstrated by the various experiments run, the reaction outcomes were deeply affected by the conditions of both the balls and the vessel used for running the reaction.

Therefore, we carefully polished the inner surfaces of the milling vessels to remove any oxidized metal layers and loaded them with new stainless‐steel balls to run the reaction. We cleaned the vessels by treating them with chelating agents such as citric acid and scrubbing them with wool fibres.^[61]^ Additionally, we smoothed the surfaces by applying an abrasive powder like silica, ensuring the removal of microscopic imperfections where oxidation could occur. These treatments significantly reduced the potential for metal particles to leach into the reaction medium.^[62]^

**Scheme 1:**
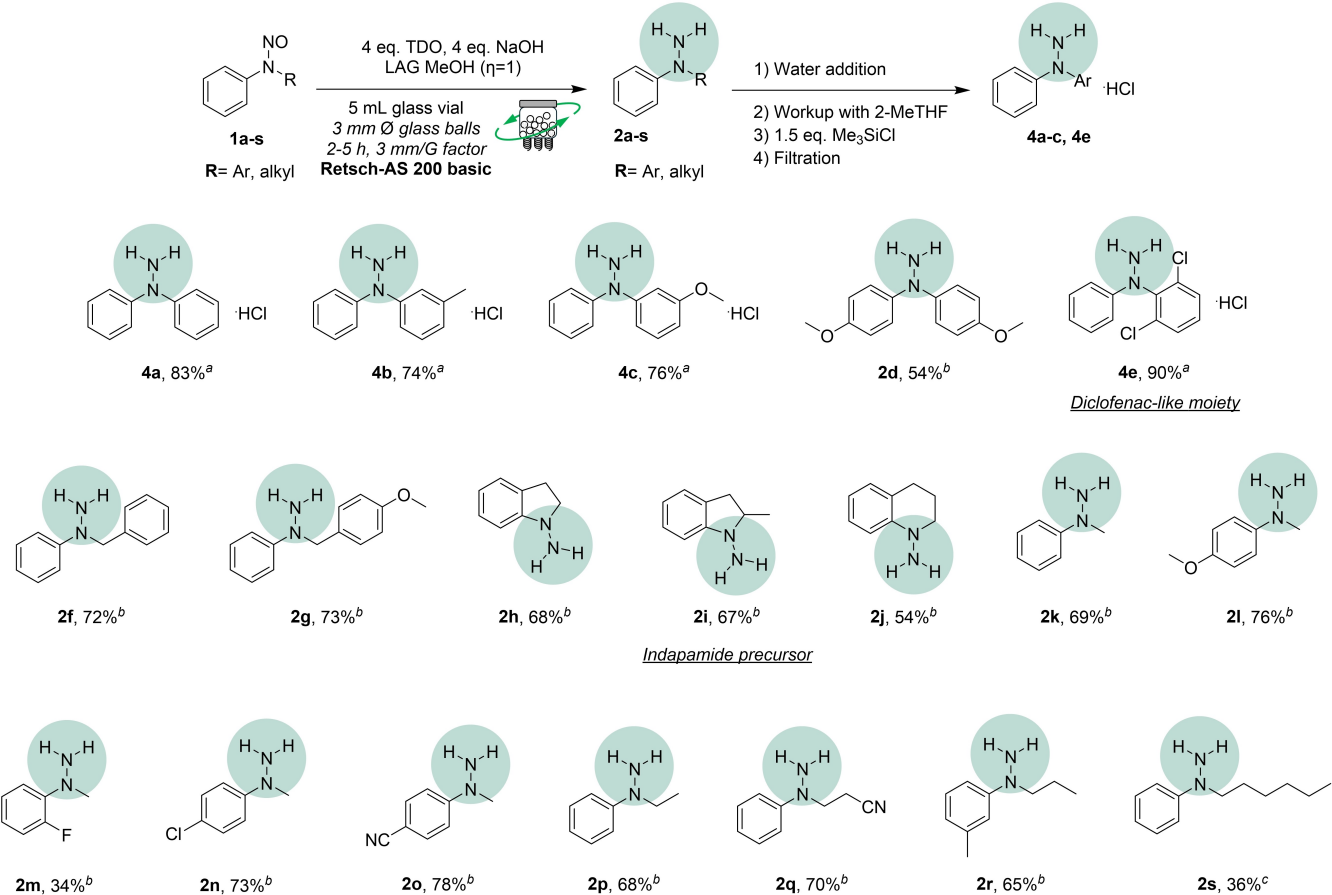
Reaction scope. Otherwise stated, all the reactions were run in a Retsch‐AS200 basic by using 1.00 mmol of **1 a–1 s** in a 5 mL glass vial equipped with 3 mm Ø glass balls (ratio number of balls: weight of the whole mixture=1 glass ball: 32 mg of reacting mixture, weight of 1 glass ball 3 mm Ø=35.6 mg). The value η (mL mg^−1^) expresses the solvent volume ratio to the weight of the reaction mixture. The yields were calculated on products isolated as themselves or as salts. (*a)* The reaction was run for 2 hours. (*b)* The reaction was run for 4 hours. (*c*) The reaction was run for 5 hours.

To simplify, the “**rusty conditions**” demonstrated the maximum impact of metal contamination, while the “**cleansed conditions**” set a standard for minimal metal interference. This comprehensive approach allowed us to isolate the variable of metal leaching and better understand its role in the mechanochemical reduction of nitrosamines (Scheme 1).

**Scheme 2:**
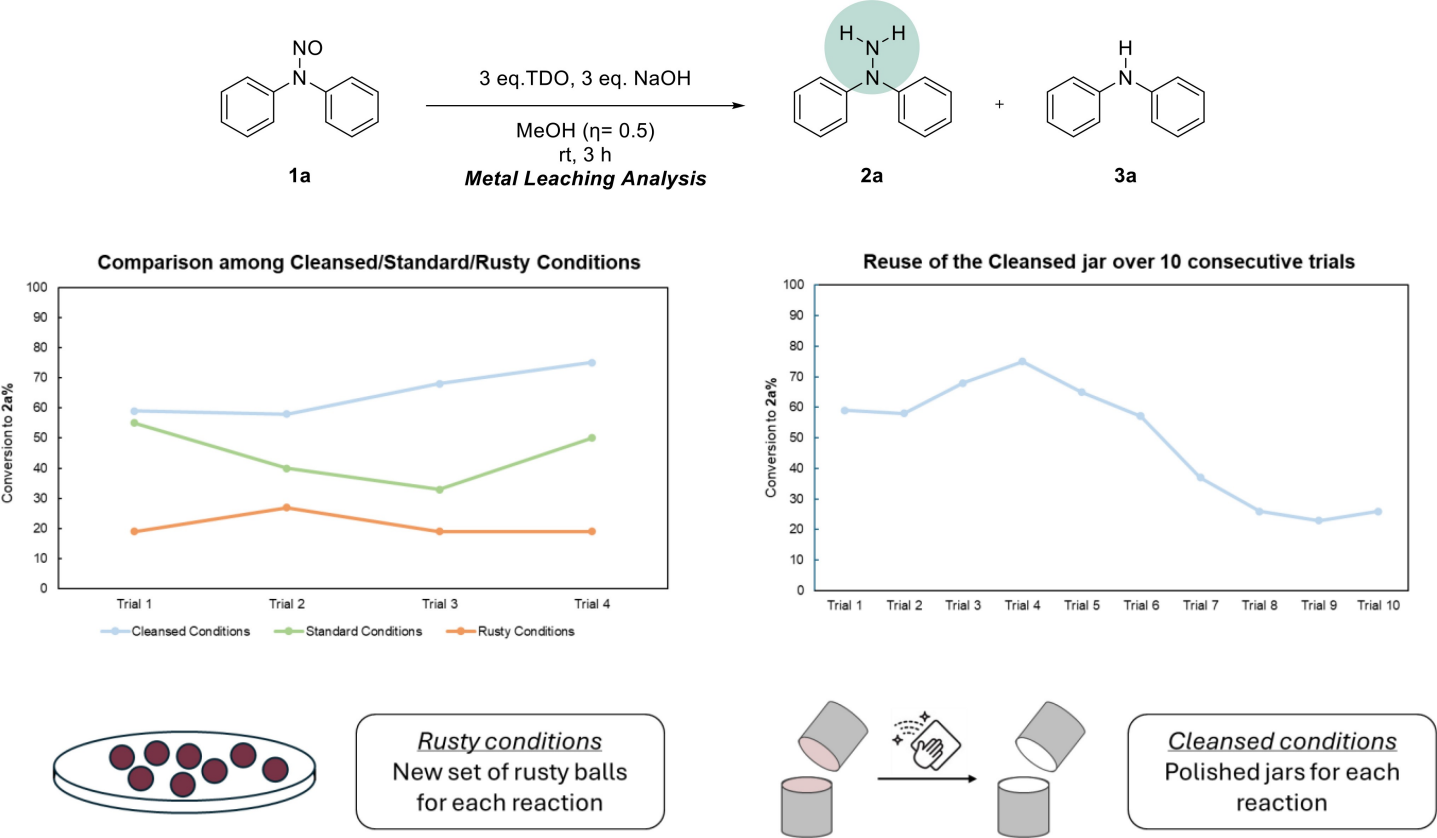
Analysis of the reaction outcomes under various conditions. Otherwise stated, all the reactions were run in a Retsch‐MM500 Vario ball mill by using 1.00 mmol of **1 a**, TDO, NaOH, and a solvent additive (*η*=0.5) in a 10 mL stainless steel jar equipped with 2 stainless‐steel balls either new or oxidised (8 mm Ø, 2.0750 g per ball) and shaken at a frequency of 30 Hz. The conversion rate was calculated through ^1^H‐NMR.

The impact of metal particles was assessed by comparing the data under standard, rusty, and cleansed milling conditions (Scheme 2, left chart). Across all experiments, a significant decrease in the formation of the desired product **2 a** was observed under rusty conditions, while obtaining the desired product was relevantly increased under cleansed conditions. Additionally, the conversion of the starting material was consistently complete in all cases.

This discovery highlights the importance of managing metal leaching to ensure reliable and accurate results in mechanochemical reductions. Adhering to the **“cleansed conditions”** protocols minimizes metal contamination, improving solvent‐free mechanochemistry′s reliability and efficiency while reducing side reactions and waste. The long‐term effects of the grinding process on the erosion of the vessel walls were also considered (Scheme 2, right chart). To assess this, we conducted the reduction of **1 a** in a jar that had been previously polished and reused it over 10 consecutive trials. Initially, we observed an increase in the formation of the desired hydrazine **2 a**; however, after the sixth trial, there was a gradual decline in the product yield, resulting in a 30 % decrease in the conversion rate by the tenth trial.^[63]^

With this data, we can finally describe a mechanochemical phenomenon, such as the leaching of metal particles from the vessel through an organic reaction. The results show that vessel erosion significantly contributes to metal contamination in the reaction environment, ultimately affecting the reaction rate and selectivity. This underscores the importance of carefully monitoring equipment wear in mechanochemical processes, as the gradual leaching of metals can profoundly impact reaction outcomes and reproducibility. Moreover, it highlights that the chemical and physical features of the grinding balls change over time during the trial run. By maintaining equipment or using alternative approaches, such as employing inert materials, we can mitigate these effects and ensure more consistent results.

**Scheme 3:**
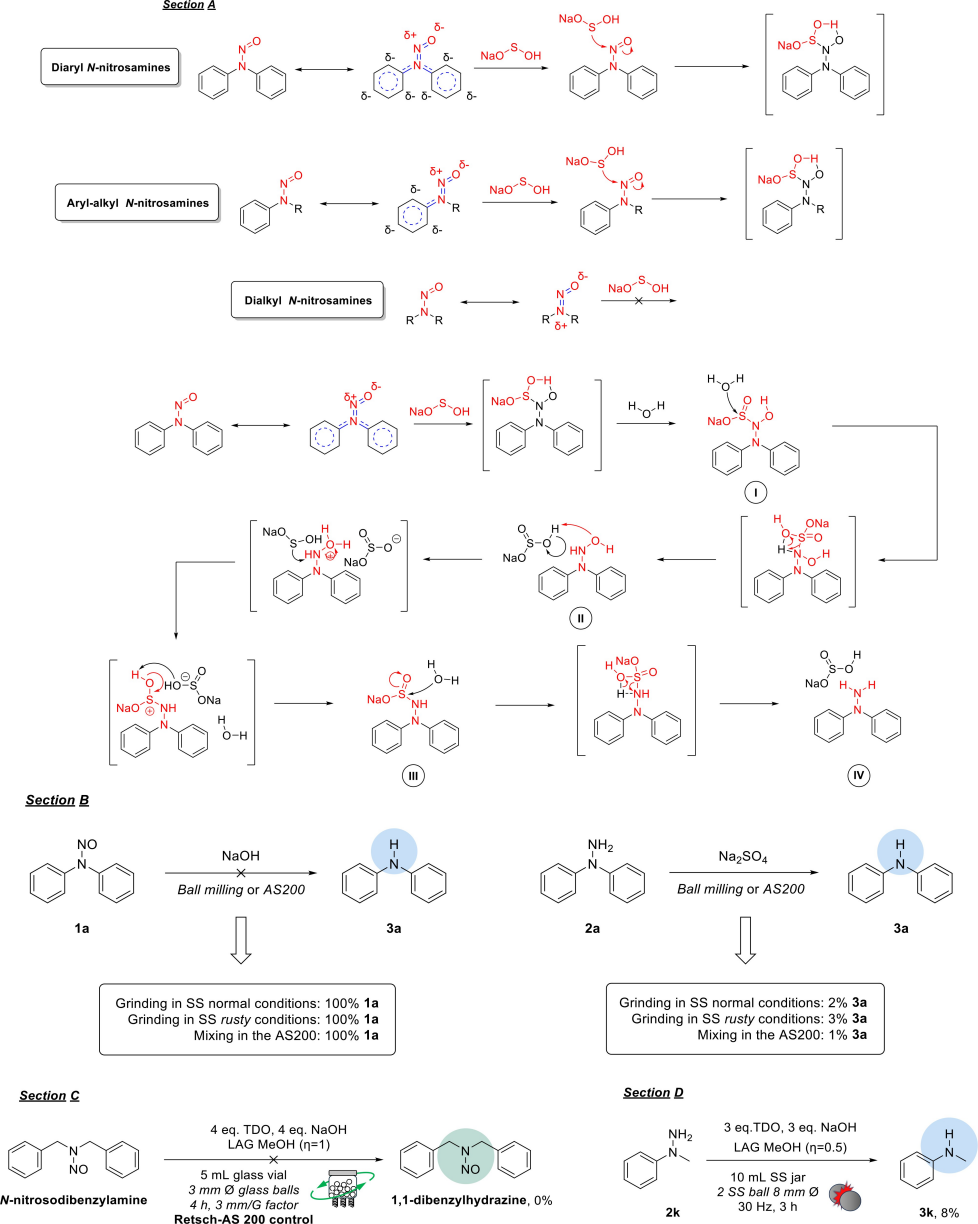
General description of the reaction mechanism with further supporting trials. (Section A) Reactivity of different *N*‐nitrosamines along with a general scheme of the reaction mechanism. (Section B) Otherwise stated, all the reactions were run in a Retsch‐MM500 Vario ball mill and a Retsch‐AS200 control using 1.00 mmol of **1 a**. (Section C) Unsuccessful reducing trial on a dialkyl substrate such as *N*‐nitrosodibenzylamine. (Section D) Lability of the reference substrate **2 k** in the presence of the reducing system TDO/NaOH.

Building on these results, we speculated about the possible role of metals in influencing the reaction outcome. First, we proposed an unreported reaction mechanism for reducing *N*‐nitrosamines with TDO (Scheme 3, section A). As expected, the driving force is the electron deficiency at the nitrogen atom of the nitroso (−NO) group. This electron deficiency is closely associated with the π‐retrodonation from the amino nitrogen. Consequently, diaryl substrates are more reactive due to the extensive delocalization of the amino nitrogen′s electrons, as demonstrated by their resonance structures, whereas aliphatic nitrosamines are less reactive. In other words, the greater the electron depletion on the functional group, the more efficiently the redox process (associated with releasing sodium hydrogen sulfoxylate from TDO) proceeds.[^4^, ^56^]

Second, we assessed the stability of nitrosamine and hydrazine under milling conditions (Scheme 3, section B). As evident from the results, products **2 a** and **3 a** appeared to be rather unsensitive under standard, rusty and AS200 conditions during simple milling with Na₂SO₄. To provide further proof of the proposed reaction mechanism, we also tested the reactivity of *N*‐nitrosodibenzylamine; however, it showed complete inertness as expected (Scheme 3, section C). Additionally, we investigated the stability of hydrazine **2 k** in the presence of TDO and NaOH to evaluate its susceptibility to the redox process (Scheme 3, section D). In this case, 8 % of the corresponding amine **3 k** was detected as a degradation product.

**Scheme 4:**
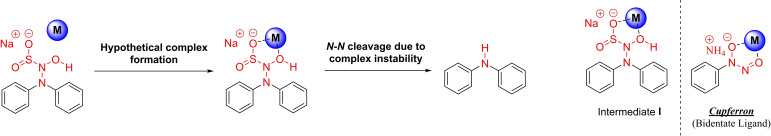
Plausible mechanism concerning the cleavage of the *N−N* bond caused by the metal particles.

Because they are less relevant to the starting and target materials, the metal particles released in the reaction medium can affect the various reaction intermediates (Scheme 4). Among the possible reasons, this kind of chemical interaction is attributed to **intermediate I** due to its resemblance to a chelating agent like *Cupferron*.[^56^, ^64^] The supposed ability of the **intermediate I** to interact with metals may result in the formation of an unstable complex that will then lead to the cleavage of the *N−N* bond.

**Scheme 5:**
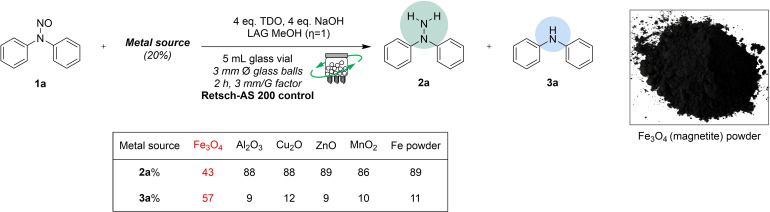
The reduction of the reference substrate **1 a** was assessed by adding various metal sources to the reaction mixture.

We attempted to identify which metals were necessary to influence the reaction outcome, demonstrating metals’ importance in this redox process. Initially, we conducted an ICP analysis of the inorganic components of the original crude mixture (detailed in the ESI file). We conducted our experiment by placing various metal derivatives into glass vials under the conditions specified in AS200 (Scheme 5). Most metals did not significantly impact the reaction outcome, except for Fe_3_O_4_ (magnetite), which showed a higher conversion rate of approximately 57 % to the aminic subproduct (see further details in the ESI file).

## Conclusions

To summarize, we have developed a protocol for synthesizing unsymmetrical hydrazines using a redox process based on thiourea dioxide. This new methodology demonstrates greater sustainability and milder reaction conditions for preparing unsymmetrical hydrazines and even allows for synthesizing the Indapamide precursor. Furthermore, we have studied this reactivity under different solvent‐free conditions, which has enabled us to assess its fundamental parameters and gain insight into the role of metal leaching in mechanochemical reactions. Our various experiments show that the significant release of metal particles leads to a complete alteration of the reaction outcome, possibly due to the reactivity of the reaction intermediates. Finally, it has been suggested that metal particles in the glass vials used under the specified conditions can cause a different conversion rate when magnetite is present in the reaction mixture.

## Conflict of Interests

The authors declare no conflict of interest.

1

## Supporting information

As a service to our authors and readers, this journal provides supporting information supplied by the authors. Such materials are peer reviewed and may be re‐organized for online delivery, but are not copy‐edited or typeset. Technical support issues arising from supporting information (other than missing files) should be addressed to the authors.

Supporting Information

## Data Availability

The data that support the findings of this study are available in the supplementary material of this article.
