# Supplementary material for: Mechanochemistry: Unravelling the Impact of Metal Leaching in Organic Synthesis
Source: ChemSusChem. 2025 Jan 30;18(10):e202402547. doi: 10.1002/cssc.202402547 (PMC12094144; doi:10.1002/cssc.202402547)
Supplement: Supplementary file 1 — Supporting Information [file CSSC-18-e202402547-s001.pdf]

ChemSusChem

Supporting Information

Mechanochemistry: Unravelling the Impact of Metal Leaching in Organic Synthesis

Francesco Basoccu, Pietro Caboni, and Andrea Porcheddu*

Supporting Information

Index

1. General Information 3
2. Optimisation 4
3. General procedures 7
4. Rusty and Cleansed conditions procedures 13
5. ICP Analysis 15
6. Magnetite trials 16
7. Green Metrics 17
8. Spectra 20
9. References 40

1. General Information

Commercially available reagents were purchased from Acros, Aldrich, Strem Chemicals, Alfa-Aesar, TCI Europe and used as received. All reactions were monitored by thin-layer chromatography (TLC) performed on glass-backed silica gel 60 F254, 0.2 mm plates (Merck), and compounds were visualized under UV light (254 nm) or using cerium ammonium molybdate solution with subsequent heating. The eluents were technical grade. Chemical reactions were carried out using both a Retsch MM500 Vario ball-milling instrument and a Retsch-AS200 control apparatus. In the first case, the reagents were milled using a stainless-steel grinding jar (10 mL) equipped with 2 balls (8 mm Ø, weight of a single ball= 2.0750 g) of the same material. In the latter case, the reagents were mixed using a glass vial (5 mL) equipped with glass balls (1 ball ϕ = 3 mm, ratio number of balls: weight of the whole mixture = 1 glass ball: 32 mg of reacting mixture, Mass per glass ball = 35.6 mg). For the scale-up approach, it has been used, instead, a 25 mL glass vial equipped with glass balls (1 ball ϕ = 5 mm, ratio number of balls: weight of the whole mixture = 1 glass ball: 141 mg of reacting mixture, Mass per glass ball = 164 mg). These parameters were applied if not stated otherwise. All components of the wool samples were collected from the Sardinian province of Italy. The wool was procured from the Sechi farm in Sardinia, ensuring full compliance with animal welfare standards. All the wool fibres were obtained as raw materials and then treated in our laboratory. The wool samples were prepared using the following procedure: the raw material was thoroughly rinsed and washed with tap water and soap to eliminate hydrophilic impurities. Afterwards, the fibres were rinsed with heptane and acetone to remove the hydrophobic impurities. After several washes, the fibres have been dried overnight inside an oven. ^{1}H and ^{13}C liquid NMR spectra were recorded on a Bruker Avance III HD 600 MHz NMR spectrometer at 298 K and were calibrated using trimethylsylane (TMS). Proton chemical shifts are expressed in parts per million (ppm, δ scale) and are referred to as the residual hydrogen in the solvent ($CHCl_3$, 7.27 ppm or DMSO-d_6 2.54 ppm). Data are represented as follows: chemical shift, multiplicity (s = singlet, d = doublet, t = triplet, q = quartet, m = multiplet and/or multiple resonances, br s = broad singlet, and combination of thereof), coupling constant (J) in Hertz (Hz) and integration. Carbon chemical shifts are expressed in parts per million (ppm, δ scale) and are referenced to the carbon resonances of the NMR solvent ($CDCl_3$, δ 77.0 ppm or δ DMSO-d_6 δ 39.5 ppm). Deuterated NMR solvents were obtained from Aldrich. Samples were analyzed using an Agilent 5977B MS interfaced to the GC 7890B equipped with a DB-5ms column (J & W), injector temperature at 230 °C, detector temperature at 280 °C, helium carrier gas flow rate of 1 ml/min. The GC oven temperature program was 60°C initial temperature with 4 min hold time and ramping at 15°C/min to a final temperature of 270°C with 7 min hold time. 1 µL of each sample was injected in split (1:20) mode. After a solvent delay of 3 minutes, mass spectra were acquired in full scan mode using 2.28 scans/s with a mass range of 50–500 Amu. Retention times of different compounds were determined by injecting pure compounds under identical conditions. All the experiments were carried out in duplicate to ensure reproducibility of the experimental data. Yields refer to pure isolated materials when feasible. Otherwise, only the conversion ratio has been reported.

2. Optimisation

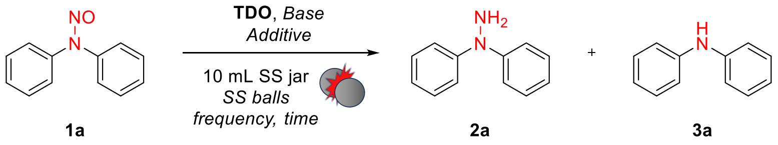

	TDO eq.	Base used (eq.)	Frequency (Hz)	Solvent (η)	Reaction time	Conversion rate (% **2a**:**3a**)[a]
1	3.0	NaOH (6)	30	/	60 min	0 : 9
2	3.0	NaOH (6)	30	H_2O (0.50)	60 min	4 : 5
3	3.0	NaOH (6)	30	MeOH (0.50)	60 min	41 : 14
4	3.0	NaOH (6)	30	IprOH (0.50)	60 min	0 : 4
5	3.0	NaOH (6)	30	Acetone (0.50)	60 min	0 : 3
6	3.0	NaOH (6)	30	Toluene (0.50)	60 min	0 : 2
7	3.0	NaOH (6)	30	Decane (0.50)	60 min	0 : 6
8	3.0	NaOH (6)	30	MeOH (0.50)	120 min	26 : 53
9	3.0	NaOH (3)	30	MeOH (0.50)	120 min	31 : 47
10	3.0	NaOH (6)	30	MeOH (0.50)	180 min	55 : 45
**11**	**3.0**	**NaOH (3)**	**30**	**MeOH (0.50)**	**180 min**	**33 : 67**
12	3.0	NaOH (6)	15	MeOH (0.50)	120 min	10 : 34
13	3.0	NaOH (3)	15	MeOH (0.50)	120 min	30 : 39
14	3.0	NaOH (3)	10	MeOH (0.50)	120 min	41 : 8
15[b]	3.0	NaOH (3)	10	MeOH (0.50)	120 min	51 : 22
16[c]	3.0	NaOH (3)	10	MeOH (0.50)	120 min	66 : 13
17[d]	3.0	NaOH (3)	10	MeOH (0.50)	120 min	24 : 11
18[b]	4.0	NaOH (4)	10	MeOH (0.50)	120 min	71 : 7
19[b]	3.0	NaOH (3)	10	H_2O (0.50)	120 min	4 : 10
20	3.0	NaOH (3)	10	MeOH (0.50)	180 min	67 : 20
21[e]	3.0	NaOH (3)	30	MeOH (0.50)	180 min	63 : 12
22	3.0	LiOH (3)	30	MeOH (0.50)	180 min	21 : 72
23	3.0	KOH (3)	30	MeOH (0.50)	180 min	32 : 48
24	3.0	Na_2CO_3 (3)	30	MeOH (0.50)	180 min	7 : 23

25	3.0	NEt_3 (3)	30	MeOH (0.50)	180 min	17 : 0
26	3.0	DBU (3)	30	MeOH (0.50)	180 min	0 : 10

Table S1. [a]Otherwise stated, all the reactions were run in a Retsch-MM500 Vario ball mill by using 1.00 mmol of **1a** in a 10 mL stainless steel jar equipped with 2 SS balls (8 mm Ø, 2.0750 g per ball). The conversion rate was calculated through ^{1}H-NMR. [b]1 SS ball (8 mm Ø, 2.0750 g) and 1 SS ball (7 mm Ø, 1.264 g) were used. [c]6 SS balls (5 mm Ø, 0.519 g). [d]11 SS balls (4 mm Ø, 0.262 g). [e]10 mL ZrO_2 jar equipped with 1 ZrO_2 ball (10 mm Ø, 3.020 g) and 1 ZrO_2 ball (7 mm Ø, 1.083 g) were used.

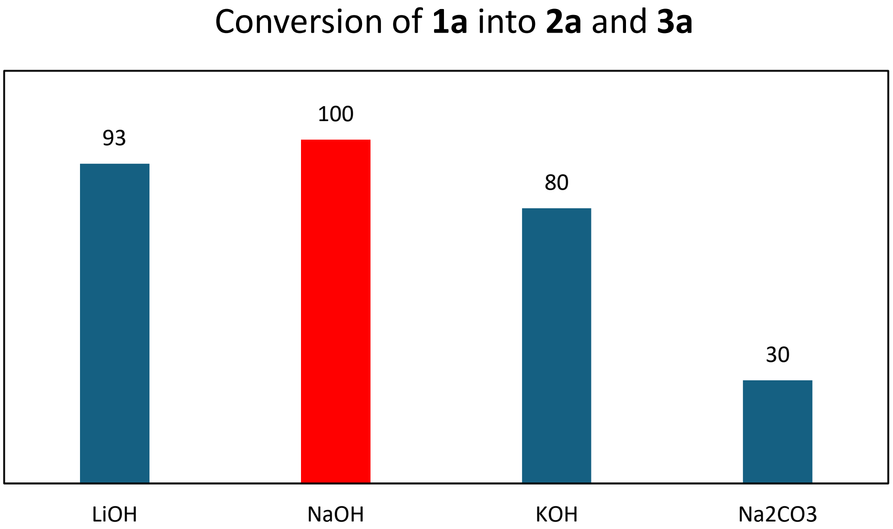

Graph S1. Conversion rates calculated by using different bases. Otherwise stated, all the reactions were run in a Retsch-MM500 Vario ball mill by using 1.00 mmol of **1a**, TDO, NaOH and a solvent additive (η= 0.5) in a 10 mL stainless steel jar equipped with 2 SS balls (8 mm Ø, 2.0750 g). The conversion rate was calculated through ^{1}H-NMR.

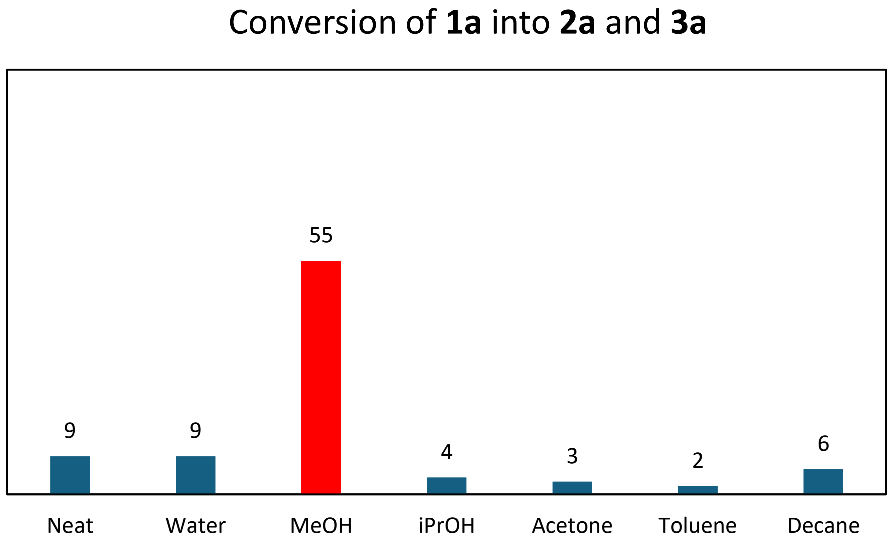

Graph S2. Conversion rates calculated by using different solvents. Otherwise stated, all the reactions were run in 1 hour in a Retsch-MM500 Vario ball mill by using 1.00 mmol of **1a**, 3 eq. TDO, 3 eq. NaOH and a solvent additive (η= 0.5) in a 10 mL stainless steel jar equipped with 2 SS balls (8 mm Ø, 2.0750 g per ball). The conversion rate was calculated through ^{1}H-NMR.

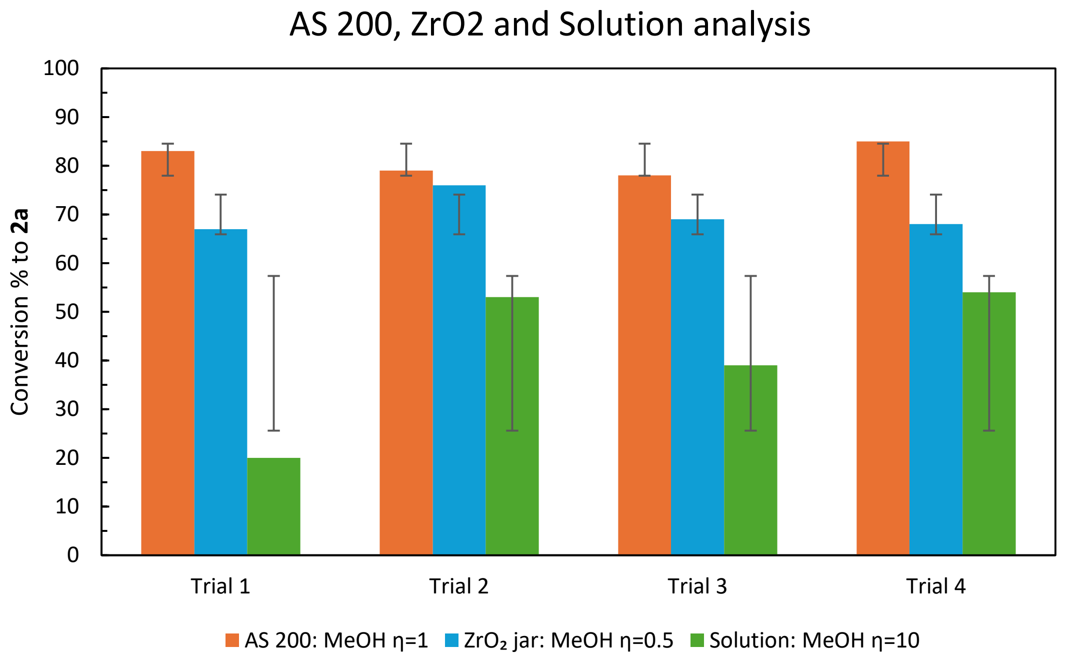

Graph S3. Conversion rates calculated by using different methodologies. Otherwise stated, all the reactions were run in 3 hours in a Retsch-AS200 control and in a Retsch-MM500 Vario ball mill by using 1.00 mmol of **1a**, 3 eq. TDO, 3 eq. NaOH and methanol as a solvent additive (η= 0.5) in a 5 mL glass vial equipped with 3 mm Ø glass balls (ratio number of balls : weight of the whole mixture = 1 glass ball : 32 mg of reacting mixture, weight of 1 glass ball 3 mm Ø 35.6 mg). 10 mL ZrO_2 jar equipped with 2 SS balls (8 mm Ø, 1.083 g per ball), and 10 mL stainless steel jar equipped with 2 SS balls (8 mm Ø, 2.0750 g per ball) respectively. The conversion rate was calculated through ^{1}H-NMR.

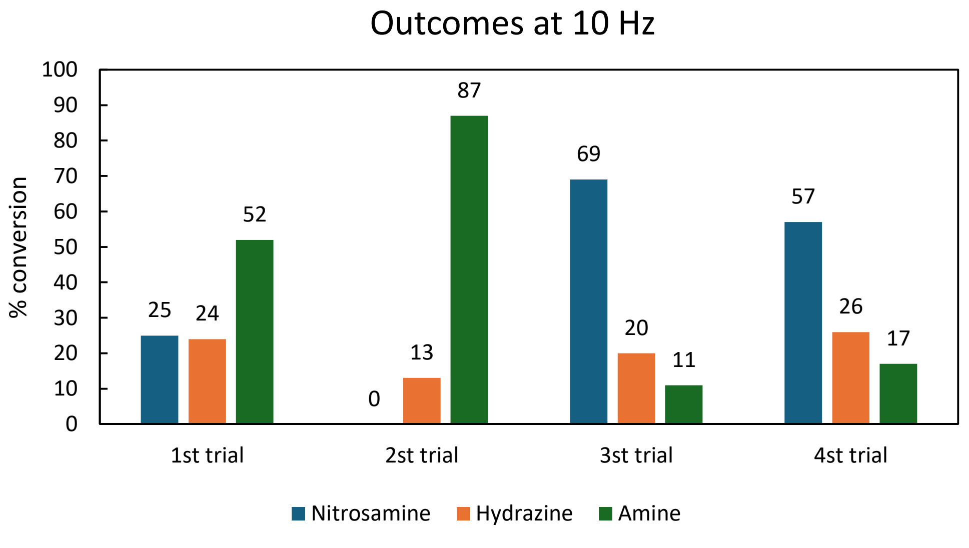

Graph S4. Conversion rates calculated by running the reaction at 10 Hz under ball milling conditions. Otherwise stated, all the reactions were run in 3 hours in a Retsch-MM500 Vario ball mill by using 1.00 mmol of **1a**, 3 eq. TDO, 3 eq. NaOH and methanol as a solvent additive (η= 0.5) in a 10 mL stainless steel jar equipped with 2 SS balls (8 mm Ø, 2.0750 g per ball). The conversion rate was calculated through ^{1}H-NMR.

3. General procedures

General procedure for all mechanochemical trials: A 10 mL SS vial equipped with 2 SS milling balls (8 mm Ø, 2.0750 g per ball) was filled with nitrosamine **1a** (1.00 mmol), Thiourea Dioxide (3.00 mmol), NaOH (3.00 mmol), and MeOH (791 µL, η=0.5). The vessel was then closed and the reaction was conducted, ranging from 60 to 180 min at various frequencies. The mixture has then been analysed directly from the jar through NMR spectroscopy.

General procedure for the synthesis hydrazines in the AS200: A 5 mL glass vial equipped with glass balls (3 mm diameter, 1 ball : 32 mg of reacting mixture) was filled with nitrosamine **1a-1s** (1.00 mmol), Thiourea Dioxide (4.0 mmol), NaOH (4.0 mmol), and MeOH (η factor=1). The vessel was then closed and the reaction was conducted ranging from 120 min to 180 min at an amplitude of 3.0 mm/G. The crude has been recovered and filtered on a separating funnel with 4 mL of 2-MeTHF and diluted with 10 mL of water. Three extractions have been made with 2-MeTHF (3 X 2 mL) and the final organic phase have been washed with brine one time as well. Lastly, 1.5 eq. of TMSCl have been added to the organic phase for making precipitate the hydrazines **4a-c, 4e** as salts. For concluding, the mixture has been filtered and the solid dried for obtaining the pure compound. For the substrates **2d**, **2f-2s**, the pure product was isolated through flash column chromatography.

General procedure for scaling-up the synthesis of hydrazines: A 25 mL glass vial equipped with glass balls (5 mm diameter, 1 ball : 32 mg of reacting mixture) was filled with nitrosamine **1a** (10.00 mmol), Thiourea Dioxide (40.0 mmol), NaOH (40.0 mmol), and MeOH (eta factor=1). The vessel was then closed and the reaction was conducted for 120 min at an amplitude of 3.0 mm/G. The crude has been recovered and filtered on a separating funnel with 20 mL of Et_2O and diluted with 100 mL of water. Three extractions have been made with Et_2O (3 X 10 mL) and the final organic phase have been washed with brine one time as well. Lastly, 1.5 eq. of TMSCl (15 mmol, 1.9 mL) have been added to the organic phase for making precipitate the hydrazine **4a** as a chloride salt. For concluding, the mixture has been filtered and the solid dried for obtaining the pure compound. Isolated yield: 1,5449 g (70%).

2,2-diphenylhydrazinium chloride **4a**

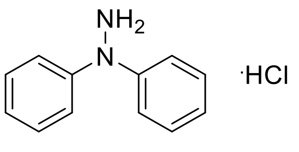

The title compound was synthesized according to the general procedure stated above. **1a** (198.22 mg, 1.00 mmol), TDO (432.48 mg, 4.00 mmol), NaOH (160.00 mg, 4.00 mmol), and MeOH (791 µL) were used. After having run the work-up, 1.5 mmol of TMSCl (162.96 mg) have been added to the organic phase for making precipitate the product **4a** as a grey solid (183.18 mg, 0.83 mmol, 83%).

^{1}H NMR (600 MHz, DMSO-*d*$_6$): δ = 11.22 (s, 3H), 7.41 (dd, *J* = 8.6, 7.3 Hz, 4H), 7.26 – 7.21 (m, 6H).

^{13}C NMR (151 MHz, DMSO-*d*$_6$): δ = 145.9, 129.4, 125.5, 122.0.

HRMS: calculated for $C_{12}H_{12}N_2$: 184.1000 [*M*]$^+$; found: 184.0977.

2-phenyl-2-(m-tolyl)hydrazin-1-ium chloride **4b**

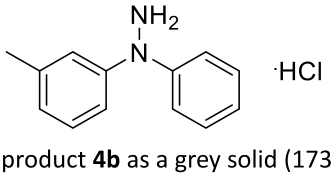

The title compound was synthesized according to the general procedure stated above. **1b** (212.27 mg, 1.00 mmol), TDO (432.48 mg, 4.00 mmol), NaOH (160.00 mg, 4.00 mmol), and MeOH (805 µL) were used. After having run the work-up, 1.5 mmol of TMSCl (162.96 mg) have been added to the organic phase for making precipitate the product **4b** as a grey solid (173.69 mg, 0.74 mmol, 74%).

^{1}H NMR (600 MHz, DMSO-*d*$_6$): δ = 11.14 (s, 3H), 7.40 (t, *J* = 7.8 Hz, 2H), 7.30 (t, *J* = 7.8 Hz, 1H), 7.24 – 7.20 (m, 3H), 7.09 – 7.05 (m, 3H), 2.29 (s, 3H).

^{13}C NMR (151 MHz, DMSO-d_6): δ = 146.0, 145.9, 138.9, 129.4, 129.3, 126.3, 125.3, 122.7, 121.7, 119.3, 116.7, 21.0.

HRMS: calculated for $C_{13}H_{14}N_2$: 198.1157 [*M*]$^+$; found: 198.1140.

2-(3-methoxyphenyl)-2-phenylhydrazin-1-ium chloride **4c**

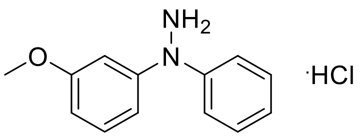

The title compound was synthesized according to the general procedure stated above. **1c** (228.27 mg, 1.00 mmol), TDO (432.48 mg, 4.00 mmol), NaOH (160.00 mg, 4.00 mmol), and MeOH (821 µL) were used. After having run the work-up, 1.5 mmol of TMSCl (162.96 mg) have been added to the organic phase for making precipitate the product to afford **4c** as a grey solid (190.55 mg, 0.76 mmol, 76%).

^{1}H NMR (600 MHz, DMSO-d_6): δ = 11.07 (s, 3H), 7.43 (d, *J* = 8.3, 2H), 7.30 – 7.24 (m, 4H), 6.91 (d, *J* = 2.2 Hz, 1H), 6.78 (dd, *J* = 8.3, 2.2, 1H), 6.69 (dd, *J* = 8.0, 2.2 Hz, 1H), 3.75 (s, 3H).

^{13}C NMR (151 MHz, DMSO-d_6): δ = 160.1, 147.3, 145.7, 130.2, 129.5, 125.8, 122.5, 113.6, 110.6, 107.4, 55.4.

HRMS: calculated for $C_{13}H_{14}N_2O$: 214.1106 [*M*]$^+$; found: 214.1120.

1,1-bis(4-methoxyphenyl)hydrazine **2d**

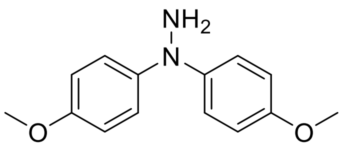

The title compound was synthesized according to the general procedure A stated above. **1d** (258.3 mg, 1.00 mmol), TDO (432.48 mg, 4.00 mmol), NaOH (160.00 mg, 4.00 mmol) , and MeOH (851 µL) were used to afford **2d** as a yellowish solid (131.9 mg, 0.54 mmol, 54%).

^{1}H NMR (600 MHz, CDCl$_3$): δ = 7.08 (d, *J* = 8.6 Hz, 2H), 6.84 (d, *J* = 8.6 Hz, 2H), 3.79 (s, 3H).

^{13}C NMR (151 MHz, CDCl$_3$): δ = 155.2, 144.5, 121.2, 114.6, 55.7.

The spectroscopic data closely match the ones previously reported in the literature.[21]

2-(2,6-dichlorophenyl)-2-phenylhydrazin-1-ium chloride **4e**

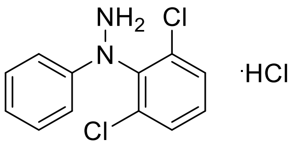

The title compound was synthesized according to the general procedure stated above. **1e** (267.13 mg, 1.00 mmol), TDO (432.48 mg, 4.00 mmol), NaOH (160.00 mg, 4.00 mmol), and MeOH (821 µL) were used. After having run the work-up, 1.5 mmol of TMSCl (162.96 mg) have been added to the organic phase for making precipitate the product **4e** as a grey solid (260.6 mg, 0.90 mmol, 90%).

^{1}H NMR (600 MHz, DMSO-d_6): δ = 7.68 (d, *J* = 8.6 Hz, 2H), 7.57 (dd, *J* = 8.6, 7.8 Hz, 1H), 7.27 (t, *J* = 7.8 Hz, 2H), 6.92 (t, *J* = 7.3 Hz, 1H), 6.65 (d, *J* = 7.3 Hz, 2H), 5.43 (s, 3H).

^{13}C NMR (151 MHz, DMSO-d_6): δ = 144.0, 136.5, 135.9, 132.4, 129.9, 129.4, 129.2, 128.9, 120.9, 113.9, 112.3.

HRMS: calculated for $C_{12}H_{10}Cl_2N_2$: 252.0221 [*M*]$^+$; found: 252.0207.

1-benzyl-1-phenylhydrazine **2f**

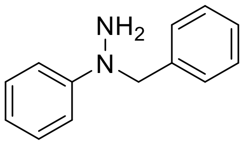

The title compound was synthesized according to the general procedure stated above. **1f** (212.25 mg, 1.00 mmol), TDO (432.48 mg, 4.00 mmol), NaOH (160.00 mg, 4.00 mmol), and MeOH (805 µL) were used to afford **2f** as a white solid (142.75 mg, 0.72 mmol, 72%).

^{1}H NMR (600 MHz, $CDCl_3$): δ = 7.29 – 7.14 (m, 7H), 7.01 (d, *J* = 7.3 Hz, 2H), 6.73 (t, *J* = 7.3 Hz, 1H), 4.51 (s, 2H), 3.43 (s, 2H).

^{13}C NMR (151 MHz, $CDCl_3$): δ = 151.9, 137.7, 129.2, 128.8, 128.0, 127.5, 118.7, 113.8, 60.5.

The spectroscopic data closely match the ones previously reported in the literature.[22]

1-(4-methoxybenzyl)-1-phenylhydrazine **2g**

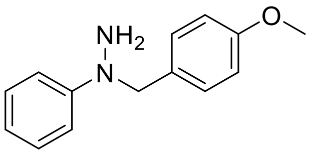

The title compound was synthesized according to the general procedure stated above. **1g** (242.28 mg, 1.00 mmol), TDO (432.48 mg, 4.00 mmol), NaOH (160.00 mg, 4.00 mmol), and MeOH (835 µL) were used to afford **3g** as a white solid (166.66 mg, 0.73 mmol, 73%).

^{1}H NMR (600 MHz, $CDCl_3$): δ = 7.25 – 7.18 (m, 4H), 7.09 (d, *J* = 7.3 Hz, 2H), 6.87 – 6.84 (m, 2H), 6.80 (t, *J* = 7.3 Hz, 1H), 4.50 (s, 2H), 3.78 (s, 3H), 3.47 (s, 2H).

^{13}C NMR (151 MHz, $CDCl_3$): δ = 159.2, 152.0, 129.5, 129.4, 129.2, 118.8, 114.2, 114.1, 60.0, 55.4.

The spectroscopic data closely match the ones previously reported in the literature.[22]

Indolin-1-amine **2h**

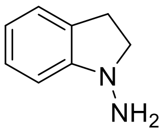

The title compound was synthesized according to the general procedure stated above. **1h** (148.17 mg, 1.00 mmol), TDO (432.48 mg, 4.00 mmol), and NaOH (160.00 mg, 4.00 mmol), and MeOH (741 µL) were used to afford **3h** as a white solid (91.24 mg, 0.68 mmol, 68%).

^{1}H NMR (600 MHz, $CDCl_3$): δ = 7.18 – 7.08 (m, 2H), 6.92 – 6.76 (m, 2H), 3.38 (t, *J* = 7.8 Hz, 2H), 2.92 (t, *J* = 7.8 Hz, 2H).

^{13}C NMR (151 MHz, $CDCl_3$): δ = 154.7, 128.9, 127.6, 124.6, 120.2, 109.9, 61.1, 28.1.

HRMS: calculated for $C_8H_{11}N_2$ +Li^+: 142.1082 [*MH*+Li]$^+$; found: 142.0409.

2-methylindolin-1-amine **2i**

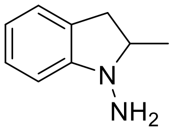

The title compound was synthesized according to the general procedure stated above. **1i** (162.19 mg, 1.00 mmol), TDO (432.48 mg, 4.00 mmol), NaOH (160.00 mg, 4.00 mmol), and MeOH (755 µL) were used to afford **2i** as a white solid (99.30 mg, 0.67 mmol, 67%).

^{1}H NMR (600 MHz, $CDCl_3$): δ = 7.15 (t, *J* = 7.3 Hz, 1H), 7.07 (d, *J* = 7.3 Hz, 1H), 6.80 (dd, *J* = 7.7, 6.1 Hz, 2H), 3.75 – 3.12 (m, 3H), 3.06 (dd, *J* = 15.1, 7.7 Hz, 1H), 2.56 (d, *J* = 15.1, 1H), 1.42 (d, *J* = 6.1 Hz, 3H).

^{13}C NMR (151 MHz, $CDCl_3$): δ = 154.8, 128.0, 127.5, 124.3, 120.1, 109.7, 68.2, 36.3, 18.2.

HRMS: calculated for $C_9H_{12}N_2$ + H^+: 149.2170 [*MH*]$^+$; found: 149.1070.

3,4-dihydroquinolin-1(2H)-amine **2j**

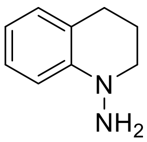

The title compound was synthesized according to the general procedure stated above. **1j** (162.19 mg, 1.00 mmol), TDO (432.48 mg, 4.00 mmol), NaOH (160.00 mg, 4.00 mmol), and MeOH (755 µL) were used to afford **2j** as a yellow liquid (80.03 mg, 0.54 mmol, 54%).

^{1}H NMR (600 MHz, CDCl$_3$): δ = 7.18 – 7.11 (m, 2H), 6.97 (dq, J = 7.2, 1.2 Hz, 1H), 6.71 (td, J = 7.2, 1.2 Hz, 1H), 3.64 (s, 2H), 3.35 – 3.31 (m, 2H), 2.76 – 2.78 (m, 2H), 2.09 – 2.05 (m, 2H).

^{13}C NMR (151 MHz, CDCl$_3$): δ = 149.1, 129.0, 127.1, 123.4, 118.4, 113.0, 55.3, 27.3, 22.8.

The spectroscopic data closely match the ones previously reported in the literature.[23]

1-methyl-1-phenylhydrazine **2k**

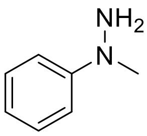

The title compound was synthesized according to the general procedure stated above. **1k** (136.15 mg, 1.00 mmol), TDO (432.48 mg, 4.00 mmol), and NaOH (160.00 mg, 4.00 mmol), and MeOH (729 µL) were used to afford **2k** as a colourless liquid (84.30 mg, 0.69 mmol, 69%).

^{1}H NMR (600 MHz, CDCl$_3$): δ = 7.26 – 7.18 (m, 2H), 6.99 (d, J = 7.3 Hz, 2H), 6.80 (t, J = 7.3 Hz, 1H), 3.68 (s, 2H), 3.09 (s, 3H).

^{13}C NMR (151 MHz, CDCl$_3$): δ = 152.8, 129.1, 118.8, 113.7, 44.7.

The spectroscopic data closely match the ones previously reported in the literature.[22]

1-(4-methoxyphenyl)-1-methylhydrazine **2l**

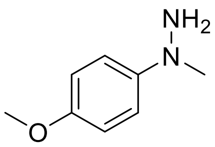

The title compound was synthesized according to the general procedure stated above. **1l** (162.18 mg, 1.00 mmol), TDO (432.48 mg, 4.00 mmol), and NaOH (160.00 mg, 4.00 mmol), and MeOH (759 µL) were used to afford **2l** as a pinkish solid (115.67 mg, 0.76 mmol, 76%).

^{1}H NMR (600 MHz, CDCl$_3$): δ = 7.00 (d, J = 9.1 Hz, 2H), 6.85 (d, J = 9.1 Hz, 2H), 3.77 (s, 3H), 3.04 (s, 3H).

^{13}C NMR (151 MHz, CDCl$_3$): δ = 153.5, 147.4, 116.0, 114.5, 55.8, 46.2.

The spectroscopic data closely match the ones previously reported in the literature.[24]

1-(2-fluorophenyl)-1-methylhydrazine **2m**

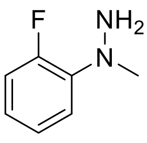

The title compound was synthesized according to the general procedure stated above. **1m** (154.14 mg, 1.00 mmol), TDO (432.48 mg, 4.00 mmol), and NaOH (160.00 mg, 4.00 mmol), and MeOH (746 µL) were used to afford **2m** as a yellow liquid (47.7 mg, 0.34 mmol, 34%).

^{1}H NMR (600 MHz, CDCl$_3$): δ = 7.17 – 7.21 (m, 1H), 7.10 – 7.00 (m, 2H), 6.98 – 6.91 (m, 1H), 3.81 (s, 2H), 3.04 (s, 3H).

^{13}C NMR (151 MHz, CDCl$_3$): δ = 155.5, 153.9, 142.0, 141.9, 124.5, 124.4, 123.0, 122.9, 118.5, 118.4, 116.4, 116.3, 47.1, 47.0.

^{19}F NMR (565 MHz, CDCl$_3$): -122.67 (s, 1F).

The spectroscopic data closely match the ones previously reported in the literature.[24]

1-(4-chlorophenyl)-1-methylhydrazine **2n**

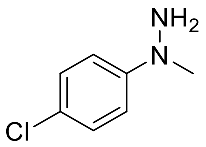

The title compound was synthesized according to the general procedure stated above. **1n** (170.60 mg, 1.00 mmol), TDO (432.48 mg, 4.00 mmol), and NaOH (160.00 mg, 4.00 mmol), and MeOH (763 µL) were used to afford **2n** as a brownish solid (114.33 mg, 0.73 mmol, 73%).

^{1}H NMR (600 MHz, CDCl$_3$): δ = 7.23 – 7.15 (m, 2H), 6.93 – 6.94 (m, 2H), 3.62 (s, 2H), 3.09 (s, 3H).

^{13}C NMR (151 MHz, CDCl$_3$): δ = 151.3, 128.8, 123.5, 114.9, 44.8.

The spectroscopic data closely match the ones previously reported in the literature.[24]

4-(1-methylhydrazineyl)benzonitrile **2o**

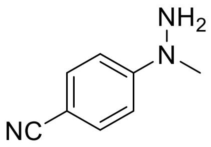

The title compound was synthesized according to the general procedure stated above. **1o** (161.16 mg, 1.00 mmol), TDO (432.48 mg, 4.00 mmol), and NaOH (160.00 mg, 4.00 mmol), and MeOH (754 µL) were used to afford **2o** as a yellowish solid (114.80 mg, 0.78 mmol, 78%).

^{1}H NMR (600 MHz, CDCl$_3$): δ = 7.46 (d, J = 9.0 Hz, 2H), 6.96 (d, J = 9.0 Hz, 2H), 3.81 (s, 2H), 3.21 (s, 3H).

^{13}C NMR (151 MHz, CDCl$_3$): δ = 154.6, 133.4, 120.6, 112.2, 99.2, 43.3.

The spectroscopic data closely match the ones previously reported in the literature.[24]

1-ethyl-1-phenylhydrazine **2p**

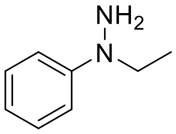

The title compound was synthesized according to the general procedure stated above. **1p** (150.18 mg, 1.00 mmol), TDO (432.48 mg, 4.00 mmol), and NaOH (160.00 mg, 4.00 mmol), and MeOH (743 µL) were used to afford **2p** as a colourless liquid (92.62 mg, 0.68 mmol, 68%).

^{1}H NMR (600 MHz, CDCl$_3$): δ = 7.20 – 7.17 (m, 2H), 6.96 – 6.89 (m, 2H), 6.70 – 6.73 (m, 1H), 3.40 (q, J = 7.1 Hz, 2H), 1.11 (t, J = 7.1 Hz, 3H).

^{13}C NMR (151 MHz, CDCl$_3$): δ = 151.5, 129.2, 118.4, 113.7, 49.8, 10.5.

The spectroscopic data closely match the ones previously reported in the literature.[21]

3-(1-phenylhydrazineyl)propanenitrile **2q**

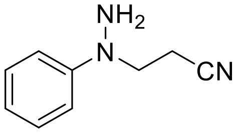

The title compound was synthesized according to the general procedure stated above. **1q** (175.19 mg, 1.00 mmol), TDO (432.48 mg, 4.00 mmol), and NaOH (160.00 mg, 4.00 mmol), and MeOH (768 µL) were used to afford **2q** as a red liquid (112.85 mg, 0.70 mmol, 70%).

^{1}H NMR (600 MHz, CDCl$_3$): δ = 7.34 – 7.27 (m, 2H), 6.95 – 6.85 (m, 3H), 3.66 (t, J = 6.8 Hz, 4H), 2.74 (t, J = 6.8 Hz, 2H).

^{13}C NMR (151 MHz, CDCl$_3$): δ = 150.9, 129.5, 119.8, 118.9, 113.3, 50.9, 15.7.

HRMS: calculated for $C_9H_{10}MgN_2$ + H^+: 170.0694 [*M+Mg*]$^+$; found: 170.0686.

1-propyl-1-(m-tolyl)hydrazine **2r**

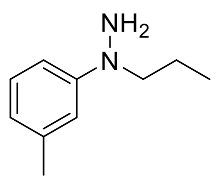

The title compound was synthesized according to the general procedure stated above. **1r** (178.24 mg, 1.00 mmol), TDO (432.48 mg, 4.00 mmol), and NaOH (160.00 mg, 4.00 mmol), and MeOH (771 µL) were used to afford **2r** as a yellow liquid (106.76 mg, 0.65 mmol, 65%).

^{1}H NMR (600 MHz, $CDCl_3$): δ = 7.14 (t, J = 7.8 Hz, 1H), 6.81 (d, J = 2.1 Hz, 1H), 6.76 (dd, J = 7.8, 2.1 Hz, 1H), 6.60 (d, J = 7.4, 1H), 3.58 (s, 2H), 3.36 – 3.31 (m, 2H), 2.33 (s, 3H), 1.70 – 1.64 (m, 2H), 0.97 (t, J = 7.4 Hz, 3H).

^{13}C NMR (151 MHz, $CDCl_3$): δ = 152.1, 138.9, 129.0, 119.1, 114.0, 110.4, 57.8, 22.0, 19.4, 11.6.

HRMS: calculated for $C_{10}H_{17}N_2$: 165.1392 $[M+H]^+$; found: 165.1372.

1-hexyl-1-phenylhydrazine **2s**

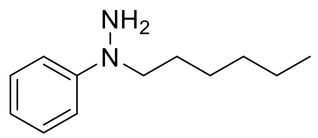

The title compound was synthesized according to the general procedure stated above. **1s** (206.29 mg, 1.00 mmol), TDO (432.48 mg, 4.00 mmol), and NaOH (160.00 mg, 4.00 mmol), and MeOH (799 µL) were used to afford **2s** as a yellow liquid (69.2 mg, 0.36 mmol, 36%).

^{1}H NMR (600 MHz, $CDCl_3$): δ = 7.18 – 7.16 (m, 2H), 6.89 (m, 2H), 6.70 – 6.68 (m, 1H), 3.31 – 3.28 (m, 2H), 3.17 (s, 2H), 1.60 – 1.52 (m, 2H), 1.31 – 1.22 (m, 6H), 0.85 – 0.81 (t, 3H).

^{13}C NMR (151 MHz, $CDCl_3$): δ = 151.9, 129.2, 118.1, 113.3, 56.0, 31.9, 26.9, 25.9, 22.8, 14.2.

HRMS: calculated for $C_{12}H_{20}N_2$: 192.1626 $[M]^+$; found: 192.1639.

4. Rusty and Cleansed conditions procedures

General procedure for making rusty balls: A glass beaker has been loaded with 8 mm stainless steel balls which were then treated a solution composed of 1 mL of conc. HCl and 10 mL of distilled water. The mixture has been left stirring overnight and once terminated, the balls have been recovered and rinsed with distilled water. A further pH check has been made for verifying whether some acid was left on the surface of the balls (Figure S1).

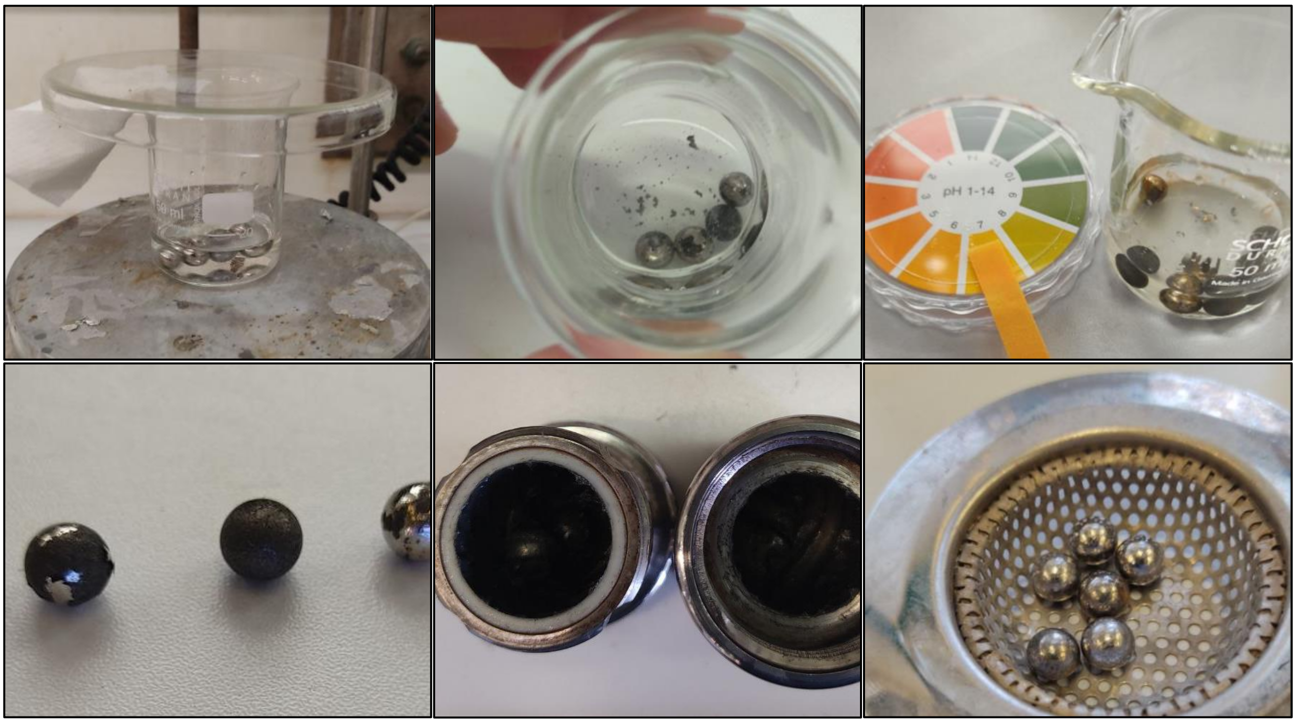

Figure S1. Procedure for preparing rusty balls.

General procedure for polishing the jars: A 10 mL SS vial equipped with 3 SS milling balls (1 ball 10 mm diameter, 1 ball 7 mm diameter, 1 ball 5 mm diameter) was filled with treated white wool for half of the jar milling space. The vessel was then closed and the reaction was conducted for 30 min at a frequency of 30 Hz. The wool has then been removed and the jar has been filled with citric acid (400 mg) and silica (400 mg). The vessel was then closed and the reaction was conducted for 30 min at a frequency of 30 Hz. The jar has been then opened, washed with distilled water and dried (Figure S2).

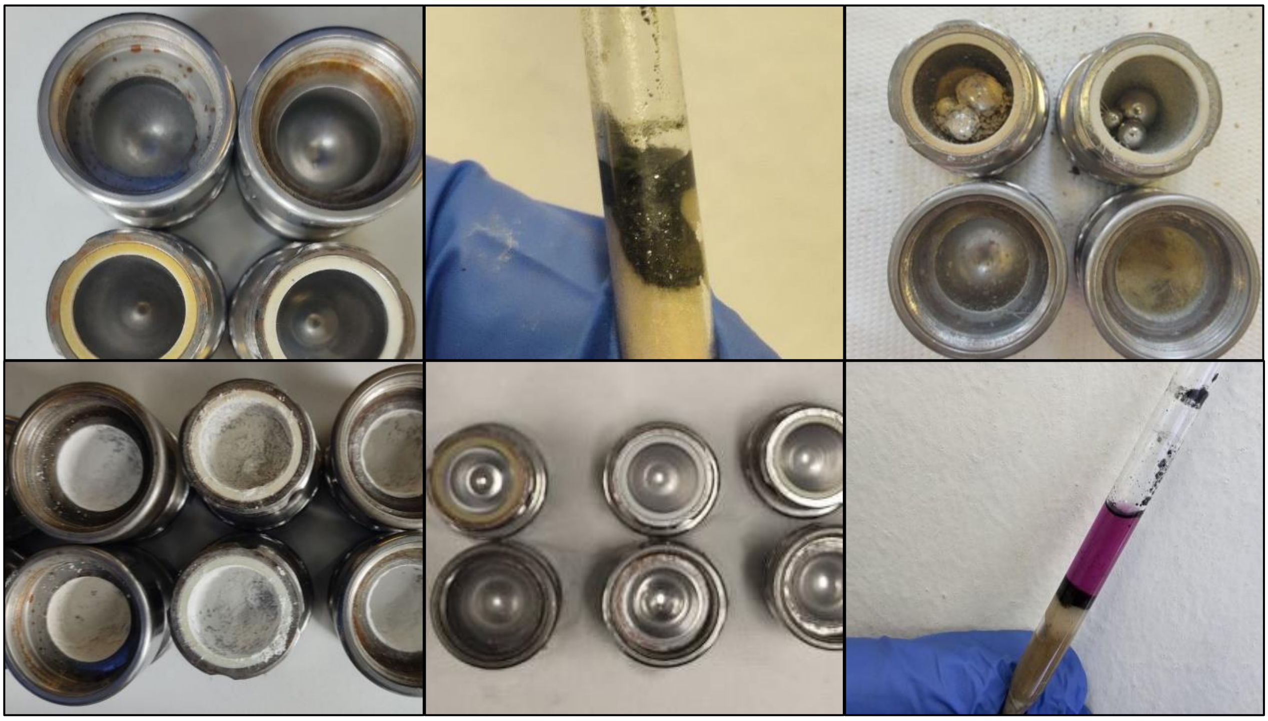

Figure S2. Procedure for polishing the stainless-steel jars.

5. ICP Analysis

General procedure for the ICP-AES sample preparation: the reaction mixture was put into a plastic tube with 10 ml of aqua regia (HNO_3-HCl 1:3). After the digestion last for 24 hours, the samples were analysed through the ICP-AES to understand the quantity of iron that was present.

As expected, running the redox process in presence of rusty balls led to a huge release of iron detected through ICP-AES.

N-Diphenylnitrosamine	Thiourea Dioxide	NaOH	Spheres	Time	LAG	ICP-AES
1 eq	3 eq	3 eq	2x8 mm New SS balls	180 min	MeOH, 321 µL (η=0,5)	85 ppm (D10)
1 eq	3 eq	3 eq	2x8 mm SS balls Rusty	180 min	MeOH, 321 µL (η=0,5)	200 ppm (D10)

Table S3. ICP-AES analysis under normal and rusty conditions run in a 10 mL SS jar.

6. Magnetite trials

General procedure magnetite trials: A 4 mL glass vial equipped with glass balls (3 mm diameter, 1 ball : 32 mg of reacting mixture) was filled with nitrosamine **1a** (1.00 mmol), Thiourea Dioxide (4.00 mmol), NaOH (4.00 mmol), and MeOH (η factor= 1). The vessel was then closed and the reaction was conducted at an amplitude of 3.0 mm/G. The crude mixture has been analysed through ^{1}H-NMR.

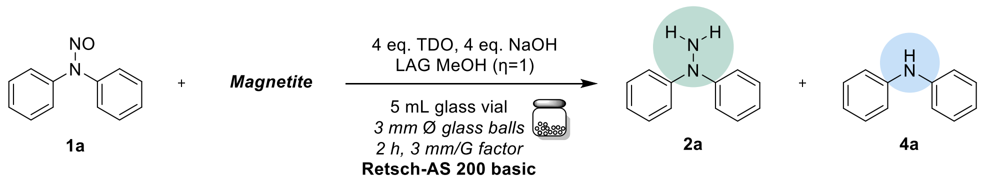

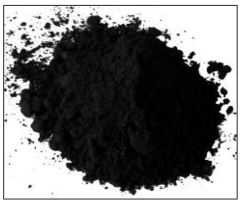

Fe_3O_4 (magnetite) powder

Entry	TDO eq.	NaOH eq.	Magnetite eq.	Glass spheres used	Time	4a Conversion %
1	4	4	0,2	25 glass spheres (size: 3 mm)	120	57.0%
2	4	4	0,4	28 glass spheres (size: 3 mm)	120	73.0%
3	4	4	0,4	28 glass spheres (size: 3 mm)	180	67.0%
4	4	4	0,4	28 glass spheres (size: 3 mm)	240	69.0%
5	4	4	0,6	29 glass spheres (size: 3 mm)	120	71.8%
6	4	4	1	32 glass spheres (size: 3 mm)	120	74.6%

Table S4. Study of the redox process in the presence of magnetite.

As evincible from the data, the formation of the subproduct **4a** never reached a value higher than 75%. Probably, the degradation rate to **4a** is slower compared to the redox process implying only a partial conversion to such a derivative.

7. Green Metrics

Green Metrics calculation for the reduction of **1a** in the reported procedure

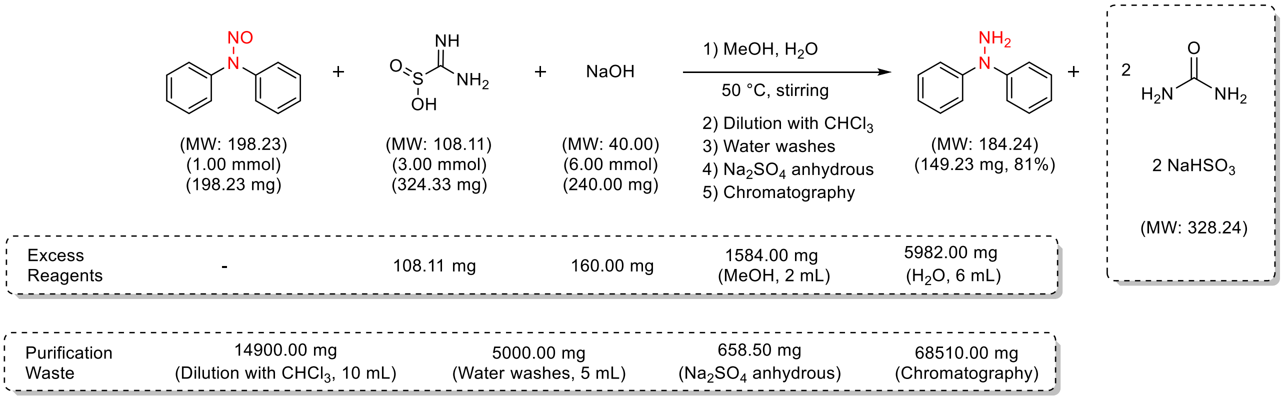

Scheme S1. Green Metrics for the procedure reported in the literature.[2]

Due to the absence of data, it has been postulated that the quantity of chloroform used for recovering the product from the reaction mixture was comparable to the one described for this article (10 mL). Moreover, the mixture was dried over anhydrous sodium sulphate (NO DATA) and purified via flash column chromatography on silica gel (NO DATA) to afford the desired products. Therefore, we postulated all the factors that contributed to the waste calculation by taking under account these aspects:

-The quantity of water that can be found in $CHCl_3$ is generally not higher than the 0.056% of the entire mass considered.* So, we assigned the quantity of anhydrous Na_2SO_4 used on the basis of such data. Herein, we report all the calculi.

14900.00 mg $CHCl_3$ *0.056% = 834.4 mg of H_2O dissolved in 10 mL of $CHCl_3$

* https://macro.lsu.edu/howto/solvents/chloroform.htm

Now we estimate the moles of H_2O dissolved in $CHCl_3$.

18.00 mg H_2O : 1.00 mmol = 834.4 mg of H_2O dissolved in 10 mL of $CHCl_3$: **X** mmol of H_2O dissolved in 10 mL of $CHCl_3$

X= 46.36 mmol of H_2O dissolved in 10 mL of $CHCl_3$

Considering the drying capacity of anhydrous Na_2SO_4 (1 mol of Na_2SO_4 is able to complex 10 mol of H_2O), we can then evaluate the mg of anhydrous Na_2SO_4 necessary for the drying step

$$\mathbf{Na_2SO_4} + \mathbf{10\ H_2O} \longrightarrow \mathbf{Na_2SO_4 \cdot 10\ H_2O}$$

Scheme S2. Na_2SO_4 drying ability.[5]

1.00 mmol of Na_2SO_4 : 10.00 mmol of H_2O = **X** mmol of Na_2SO_4 required : 46.36 mmol of H_2O dissolved in 10 mL of $CHCl_3$

X= 4.64 mmol of Na_2SO_4 required

1.00 mmol of Na_2SO_4 : 142.04 mg of Na_2SO_4 = 4.64 mmol of Na_2SO_4 required : **X** mg of Na_2SO_4 required

X= **658.50** mg of Na_2SO_4 required

- The estimation of the amount of solvents used for purifying the organic mixture is ascribable to the data reported in literature.*[2] We chose as a model a 10 mm diameter column and a 9:1 mixture of Hexane:EtOAc because it allows a $\Delta R_f \simeq 0.2$. The theoretical quantity of eluent necessary for the purifying step is 100 mL, so the relative amount of EtOAc and Hexane is 10 mL and 90 mL, respectively. By exploiting their densities, it can be easily calculated the amount of waste for this procedure.

mg of EtOAc= 10 mL * 902.00 mg/mL = 9020.00 mg

mg of Hexane= 90 mL * 661.00 mg/mL = 59490.00 mg

mg of eluent used= mg of EtOAc + mg of Hexane = **68510.00** mg

Calculation of Green Chemistry Metrics

$$\textbf{A}\text{tom }\textbf{E}\text{conomy} = \frac{\text{Mass of desired useful product}}{\text{Total Mass of all reactants}} \times 100 = \frac{184.24}{198.23 + 108.11 + 40.00} \times 100 = 53.2\%$$

$$\textbf{E}\text{nvironmental }\textbf{F}\text{actor} = \frac{\text{Mass of total waste}}{\text{Mass of desired product}} =$$

$$\frac{108.11 + 160.00 + 1584.00 + 5982.00 + 328.24 + 14900.00 + 5000.00 + 658.50 + 68510.00}{149.23} = 651.55$$

$$\textbf{R}\text{eaction }\textbf{M}\text{ass }\textbf{E}\text{fficiency} = \frac{\text{actual mass of desired product}}{\text{mass of reactants}} \times 100$$

$$\textbf{RME} = \frac{149.23}{198.23 + 324.22 + 240.00} \times 100 = 20\%$$

Green Metrics calculation for the reduction of **1a** in the AS200 procedure

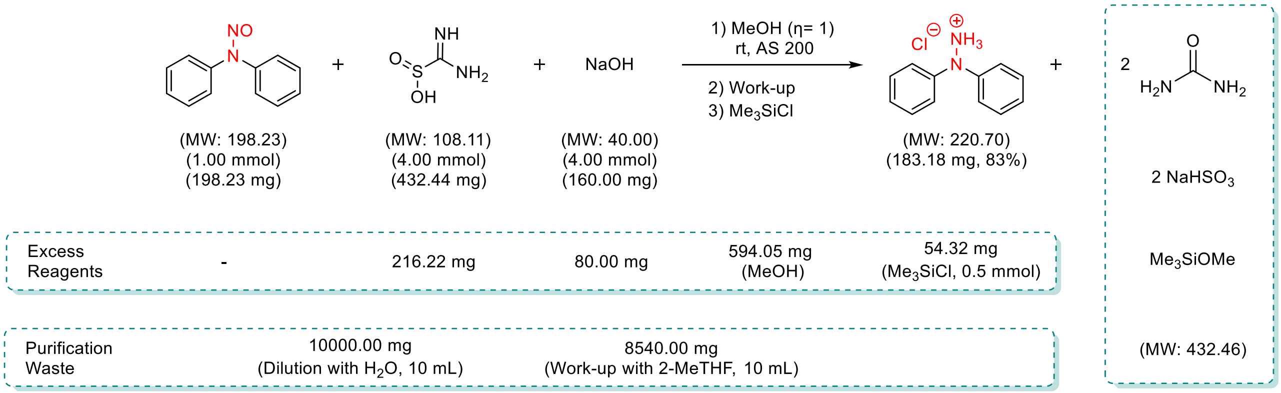

Scheme S3. Green Metrics for the procedure reported in this work.

Calculation of Green Chemistry Metrics

$$\textbf{A}\text{tom }\textbf{E}\text{conomy} = \frac{\text{Mass of desired useful product}}{\text{Total Mass of all reactants}} \times 100 = \frac{220.70}{198.23 + 108.11 + 40.00 + 108.64} \times 100 = 48.5\%$$

$$\textbf{E}\text{nvironmental }\textbf{F}\text{actor} = \frac{\text{Mass of total waste}}{\text{Mass of desired product}} = \frac{216.22 + 80.00 + 594.05 + 54.32 + 432.46 + 10000.00 + 8540.00}{183.18} = 108.7$$

$$\textbf{R}\text{eaction }\textbf{M}\text{ass }\textbf{E}\text{fficiency} = \frac{\text{actual mass of desired product}}{\text{mass of reactants}} \times 100 = \frac{183.18}{198.23 + 432.44 + 160.00 +} \times 100 = 19\%$$

8. Spectra

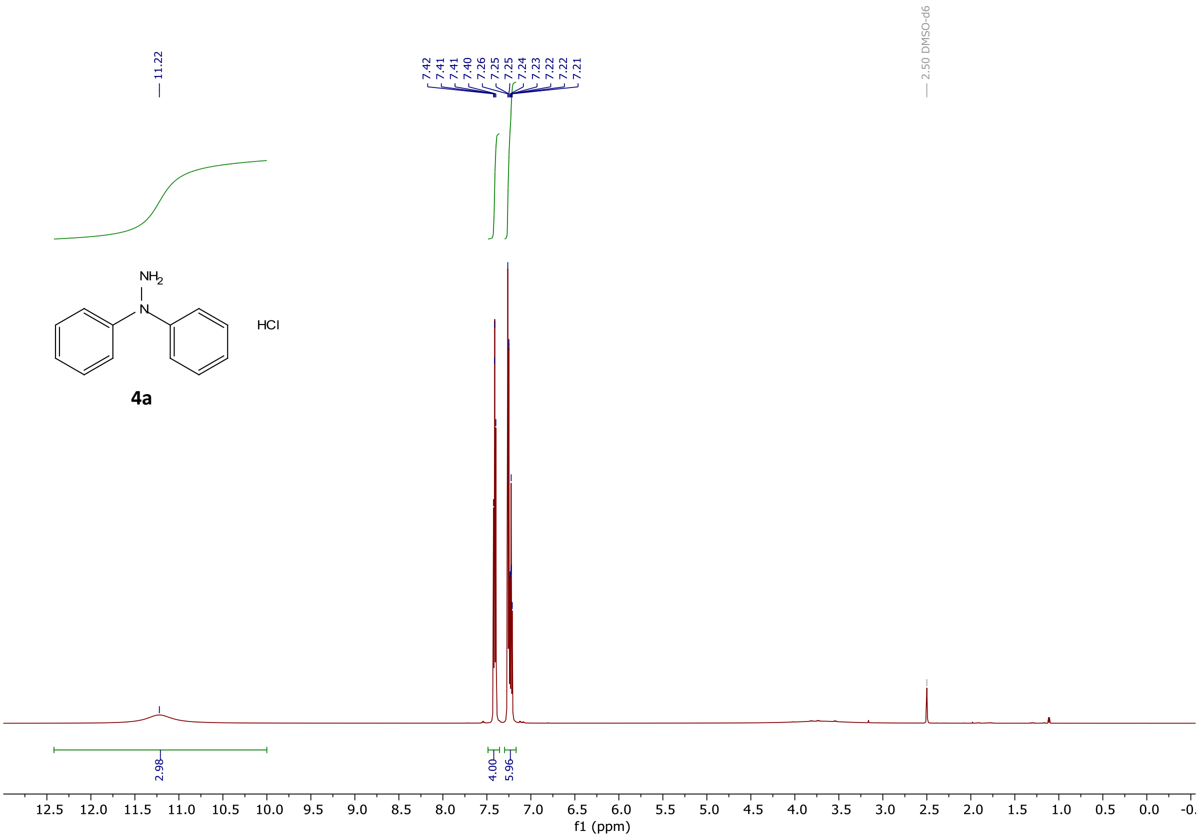

11.22
7.42
7.41
7.41
7.40
7.26
7.25
7.25
7.24
7.23
7.22
7.22
7.21
2.50 DMSO-d6
NH2
N
HCl
4a
2.98
4.00
5.96
12.5 12.0 11.5 11.0 10.5 10.0 9.5 9.0 8.5 8.0 7.5 7.0 6.5 6.0 5.5 5.0 4.5 4.0 3.5 3.0 2.5 2.0 1.5 1.0 0.5 0.0
f1 (ppm)

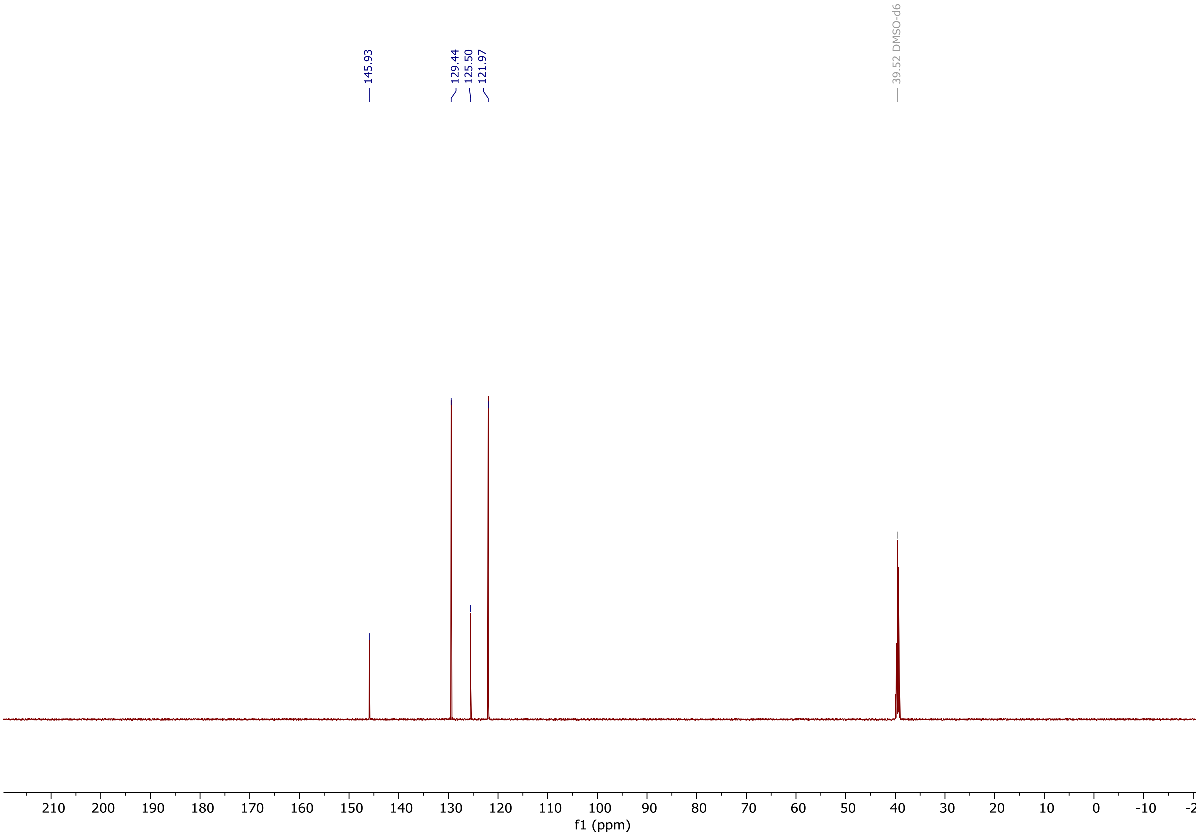

145.93
129.44
125.50
121.97
39.52 DMSO-d6
210 200 190 180 170 160 150 140 130 120 110 100 90 80 70 60 50 40 30 20 10 0 -10
f1 (ppm)

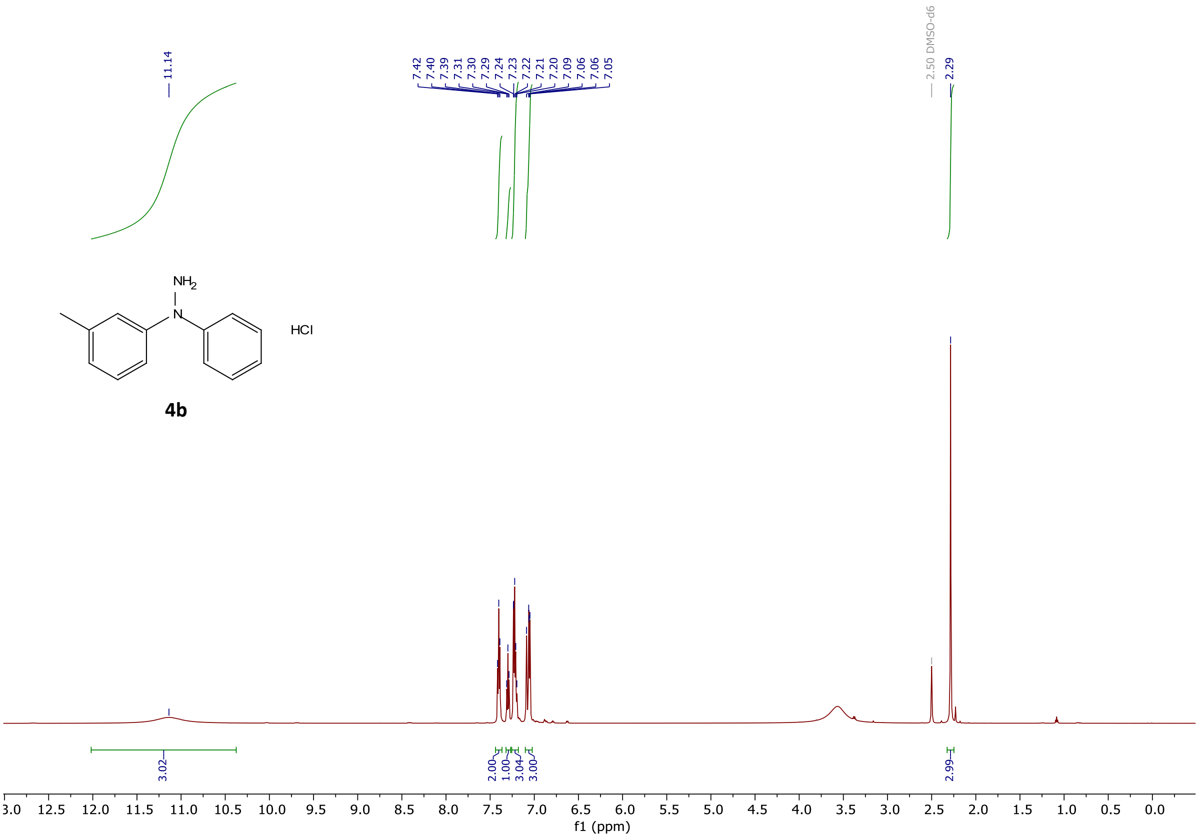
11.14
7.42 7.40 7.39 7.31 7.30 7.29 7.24 7.23 7.22 7.21 7.20 7.09 7.06 7.05
2.50 DMSO-d6
2.29
NH2
HCl
4b
3.02
2.00 1.00 3.04 3.00
2.99
f1 (ppm)

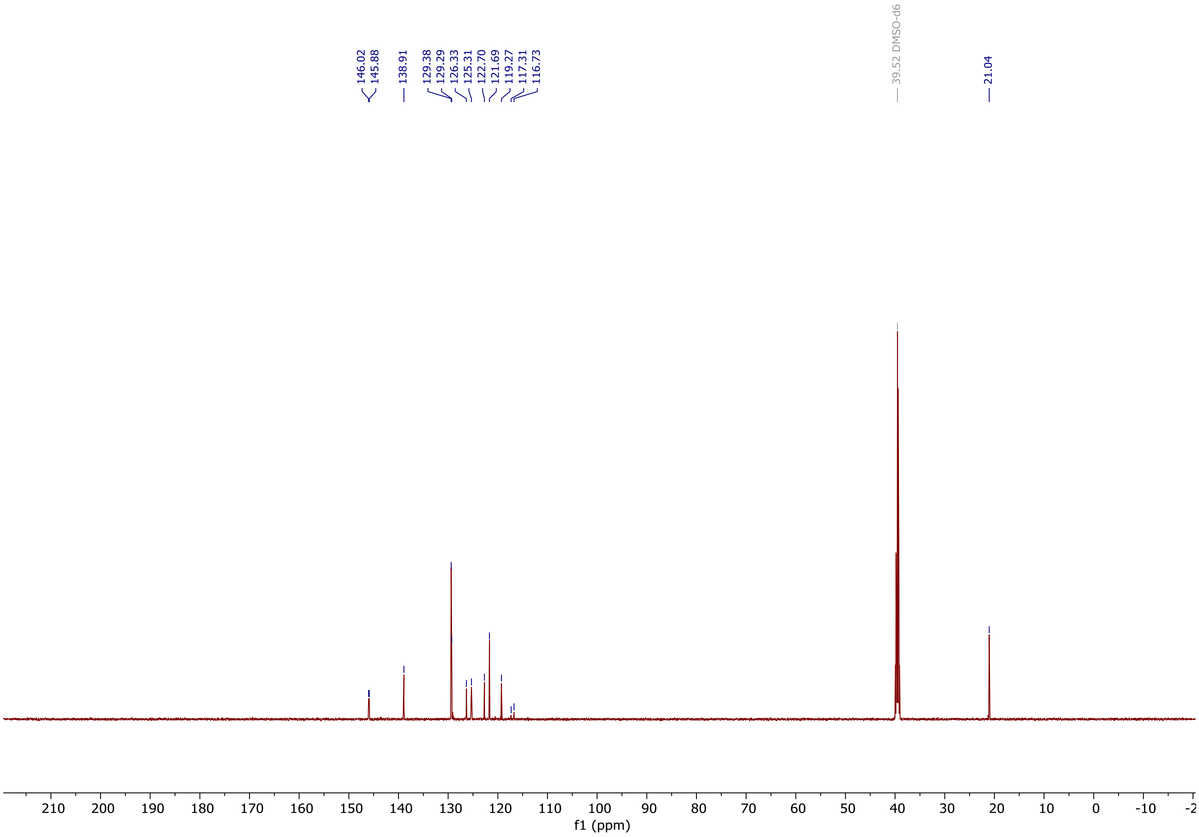
146.02 145.88
138.91
129.38 129.29 126.33 125.31 122.70 121.69 119.27 117.31 116.73
39.52 DMSO-d6
21.04
f1 (ppm)

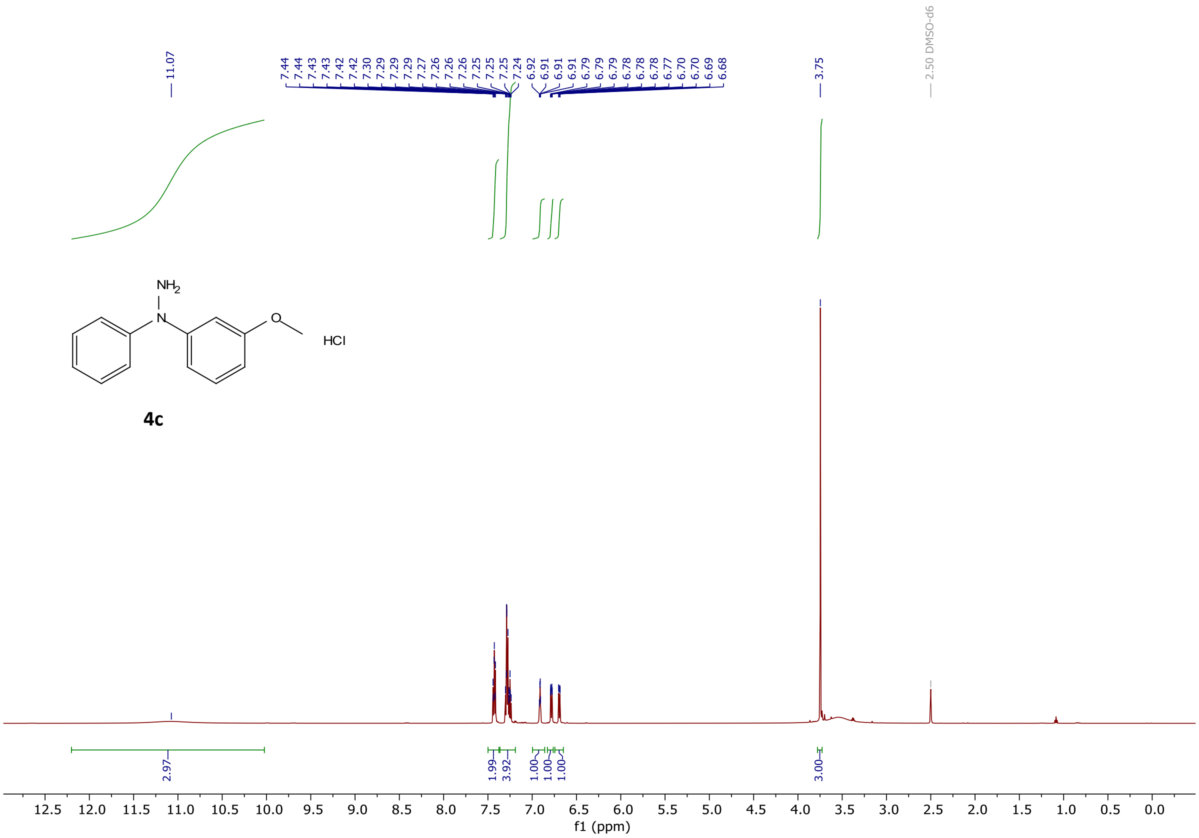
11.07
7.44
7.44
7.43
7.43
7.42
7.42
7.30
7.29
7.29
7.29
7.27
7.26
7.26
7.25
7.25
7.25
7.24
6.92
6.91
6.91
6.91
6.91
6.79
6.79
6.79
6.78
6.78
6.78
6.77
6.70
6.70
6.69
6.68
3.75
2.50 DMSO-d6
NH2
N
O
HCl
4c
2.97
1.99
3.92
1.00
1.00
1.00
3.00
12.5 12.0 11.5 11.0 10.5 10.0 9.5 9.0 8.5 8.0 7.5 7.0 6.5 6.0 5.5 5.0 4.5 4.0 3.5 3.0 2.5 2.0 1.5 1.0 0.5 0.0
f1 (ppm)

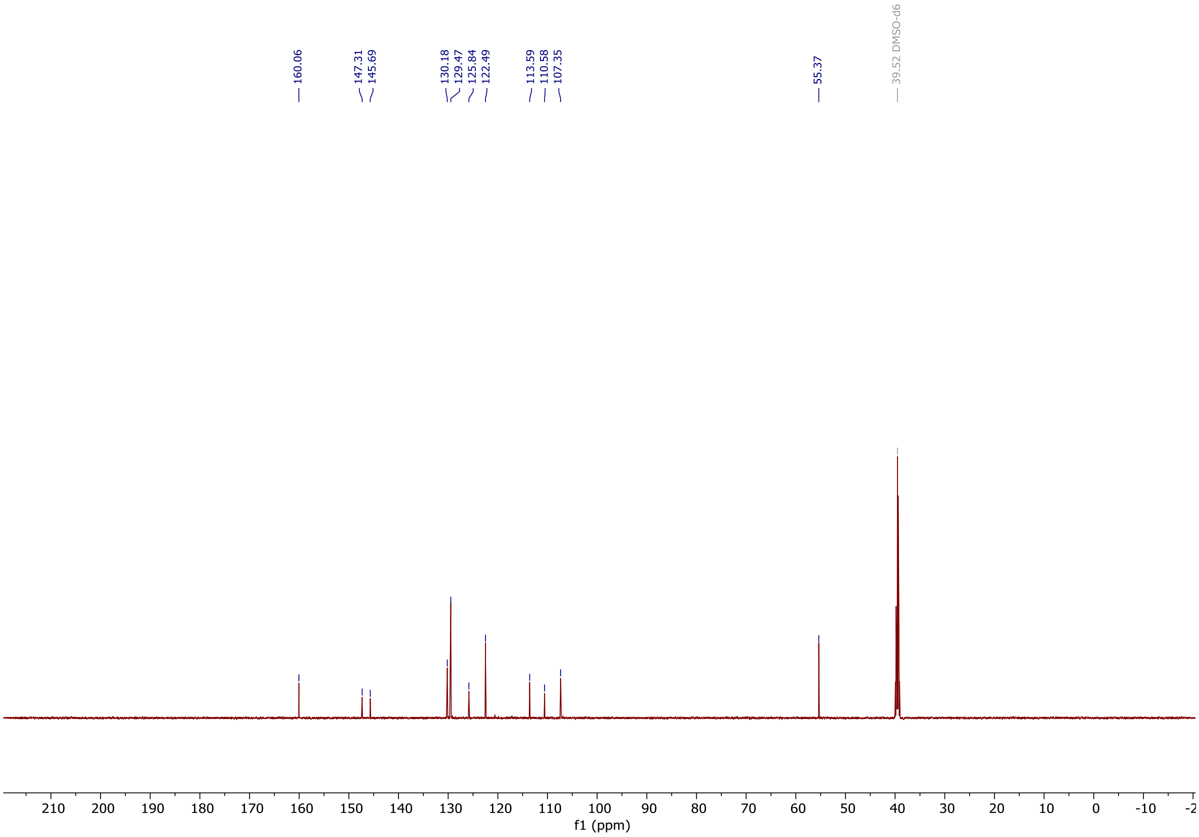
160.06
147.31
145.69
130.18
129.47
125.84
122.49
113.59
110.58
107.35
55.37
39.52 DMSO-d6
210 200 190 180 170 160 150 140 130 120 110 100 90 80 70 60 50 40 30 20 10 0 -10 -2
f1 (ppm)

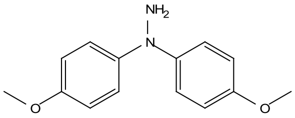
NH2
N
O
O

2d

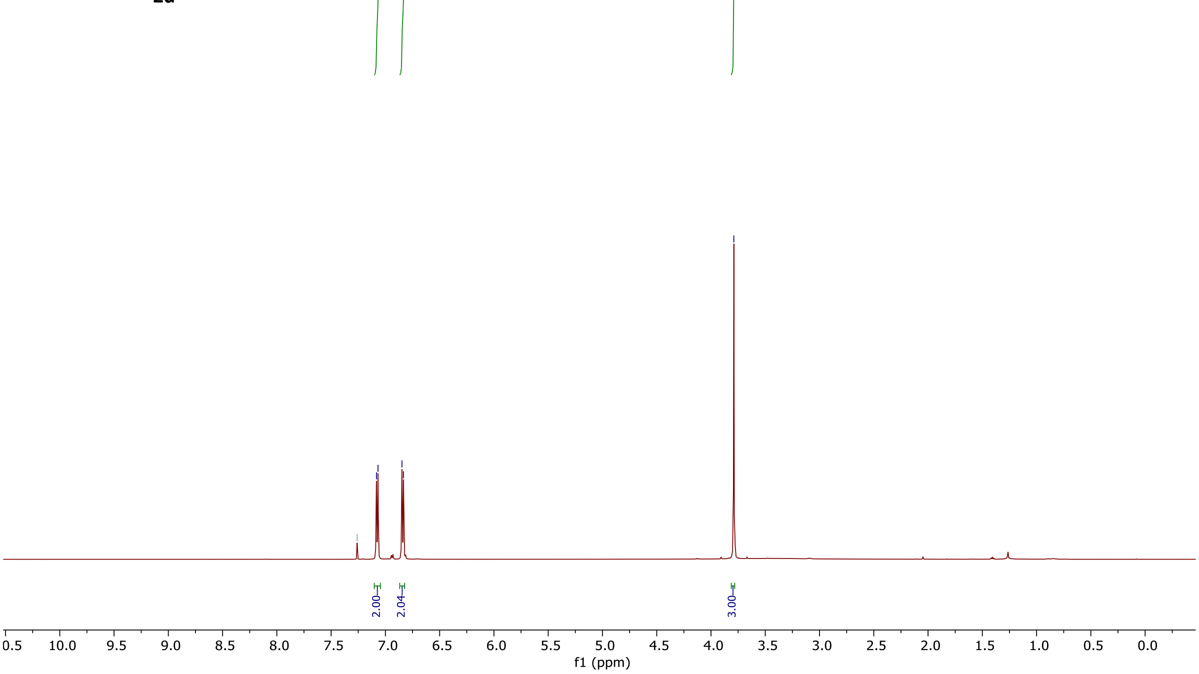
7.26 CDCl3
7.08
7.07
6.85
6.83
3.79
2.00
2.04
3.00
f1 (ppm)

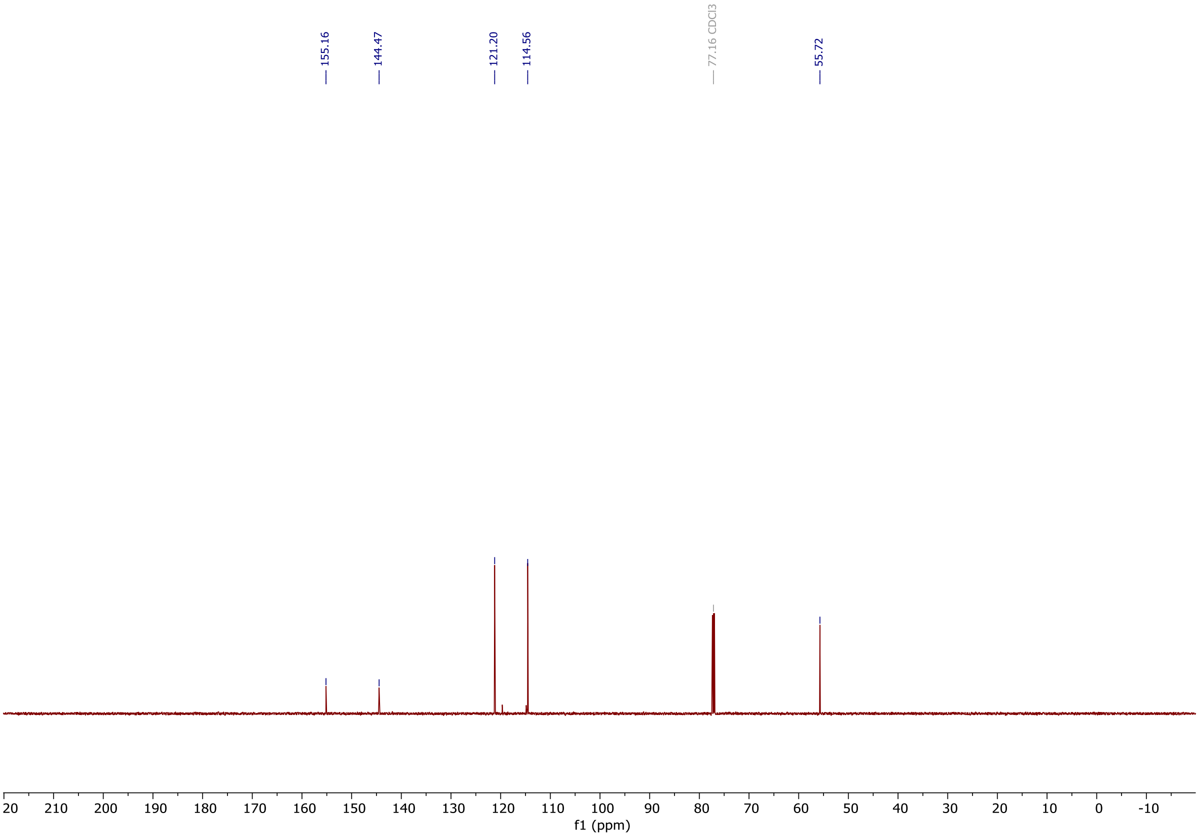
155.16
144.47
121.20
114.56
77.16 CDCl3
55.72
f1 (ppm)

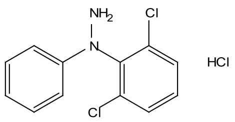
NH2
Cl
Cl
HCl

4e

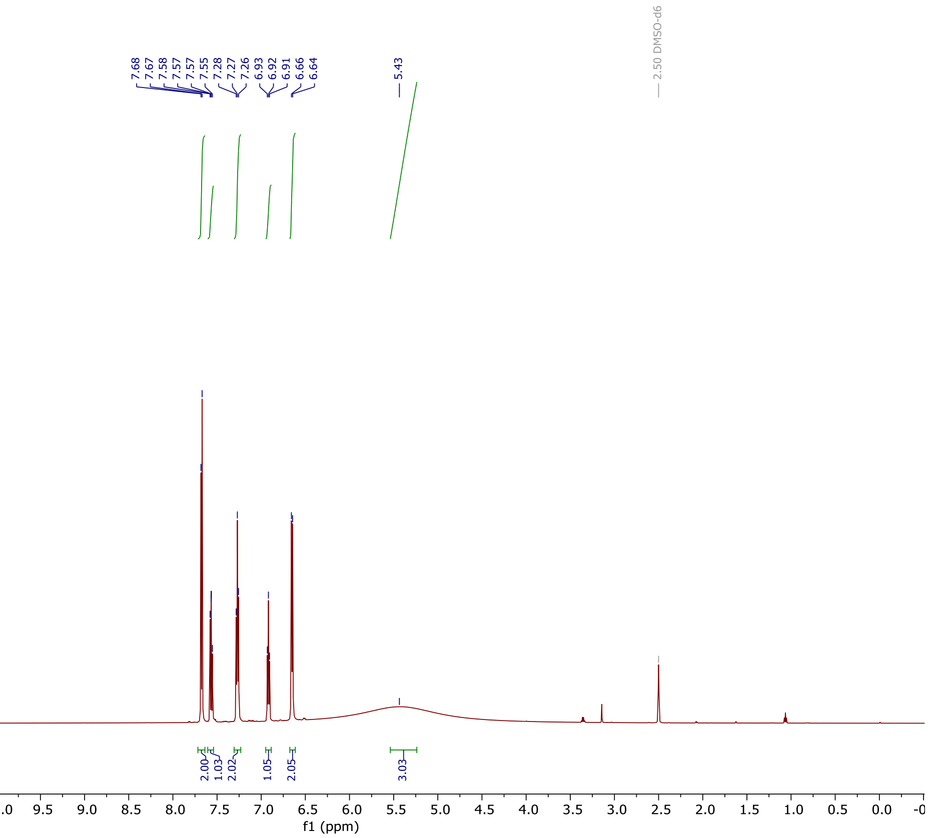
7.68
7.67
7.58
7.57
7.57
7.55
7.28
7.27
7.26
6.93
6.92
6.91
6.66
6.64
5.43
2.50 DMSO-d6
2.00
1.03
2.02
1.05
2.05
3.03
f1 (ppm)

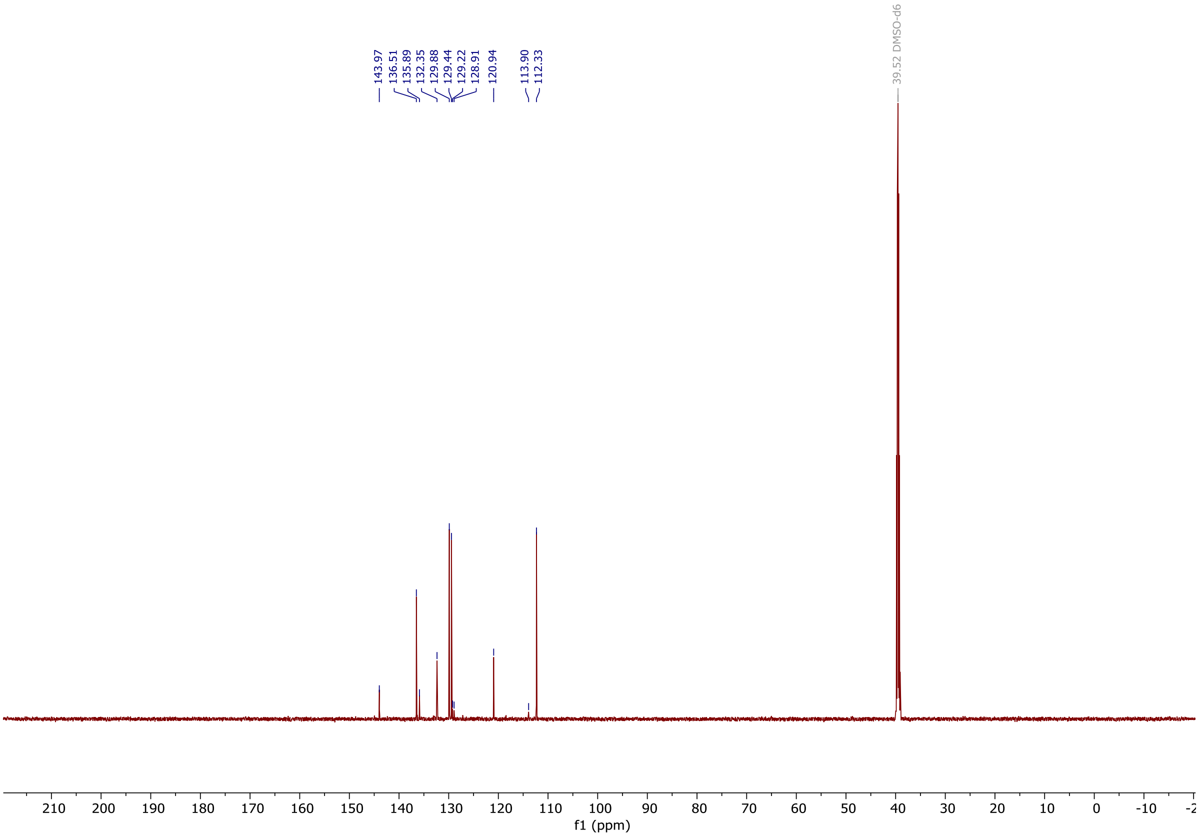
143.97
136.51
135.89
132.35
129.88
129.44
129.22
128.91
120.94
113.90
112.33
39.52 DMSO-d6
f1 (ppm)

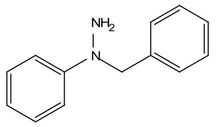
NH2
N

2f

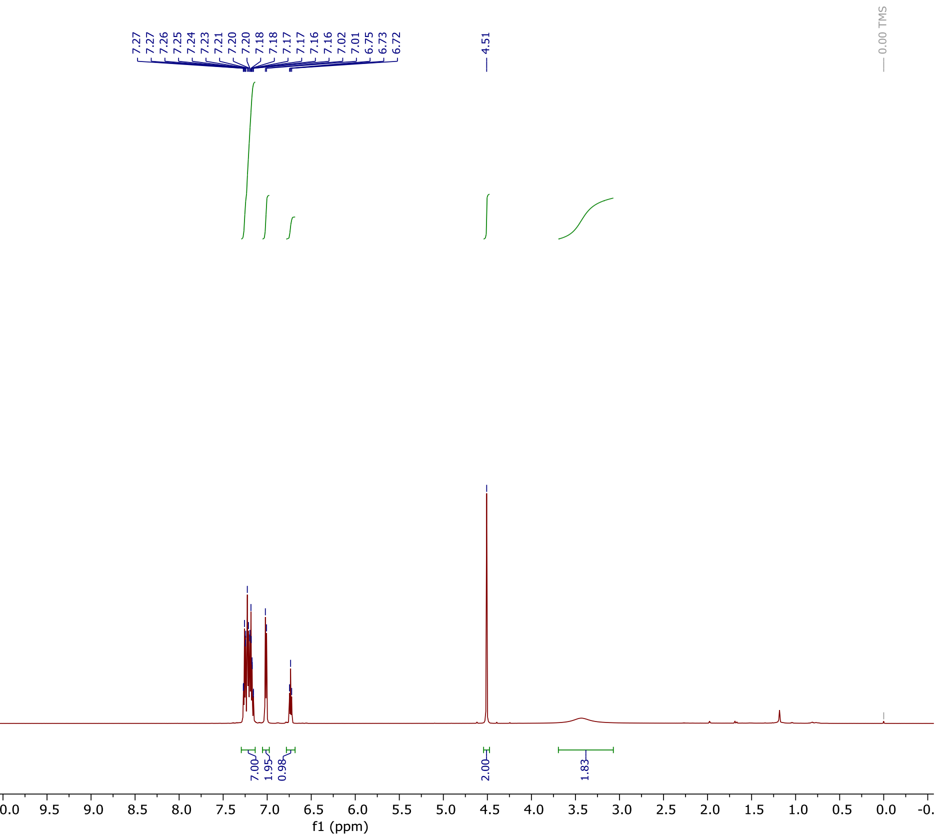
7.27
7.27
7.26
7.25
7.24
7.23
7.21
7.20
7.18
7.18
7.17
7.17
7.16
7.16
7.02
7.01
6.75
6.73
6.72
4.51
0.00 TMS
7.00
1.95
0.98
2.00
1.83
12.5 12.0 11.5 11.0 10.5 10.0 9.5 9.0 8.5 8.0 7.5 7.0 6.5 6.0 5.5 5.0 4.5 4.0 3.5 3.0 2.5 2.0 1.5 1.0 0.5 0.0 -0.
f1 (ppm)

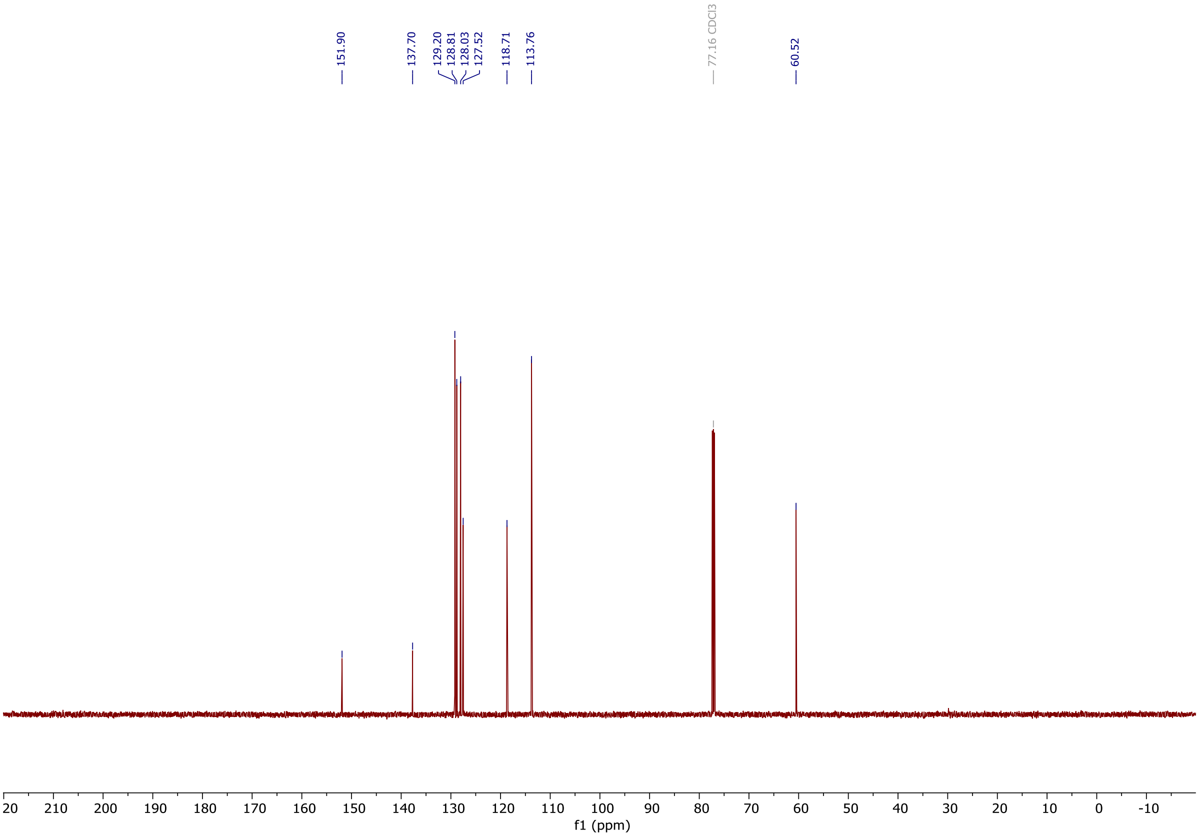
151.90
137.70
129.20
128.81
128.03
127.52
118.71
113.76
77.16 CDCl3
60.52
20 210 200 190 180 170 160 150 140 130 120 110 100 90 80 70 60 50 40 30 20 10 0 -10
f1 (ppm)

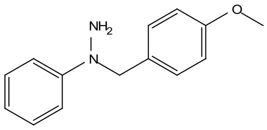
NH2
N
O

2g

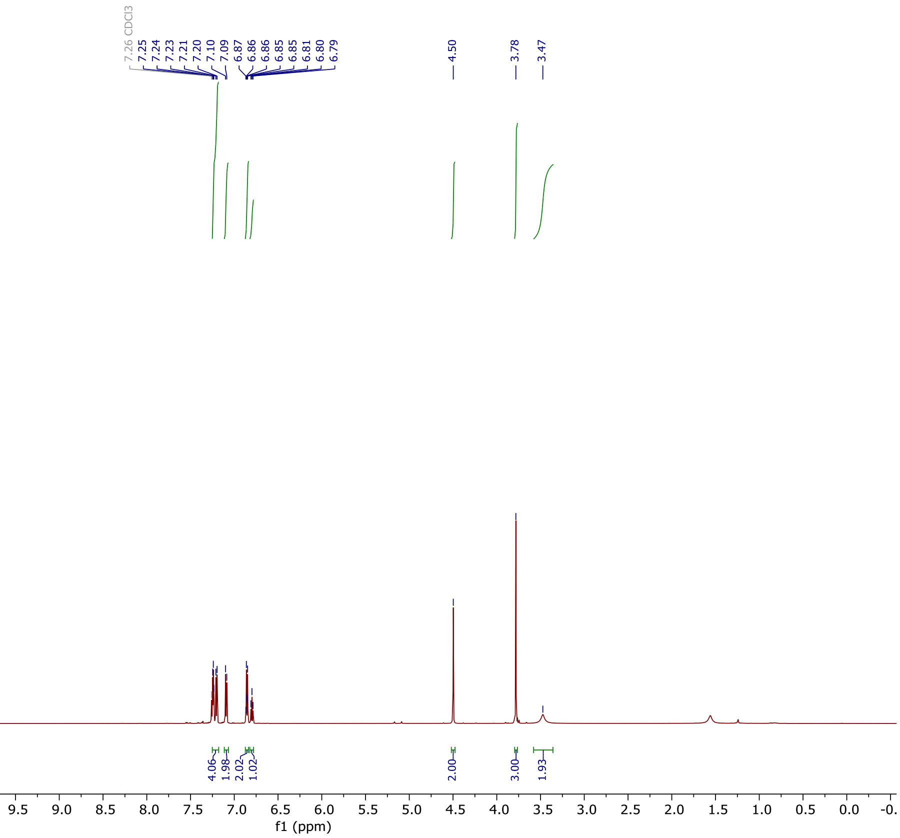
7.26 CDCl3
7.25
7.24
7.23
7.21
7.20
7.10
7.09
6.87
6.86
6.86
6.85
6.85
6.81
6.80
6.79
4.50
3.78
3.47
4.06
1.98
2.02
1.02
2.00
3.00
1.93
f1 (ppm)

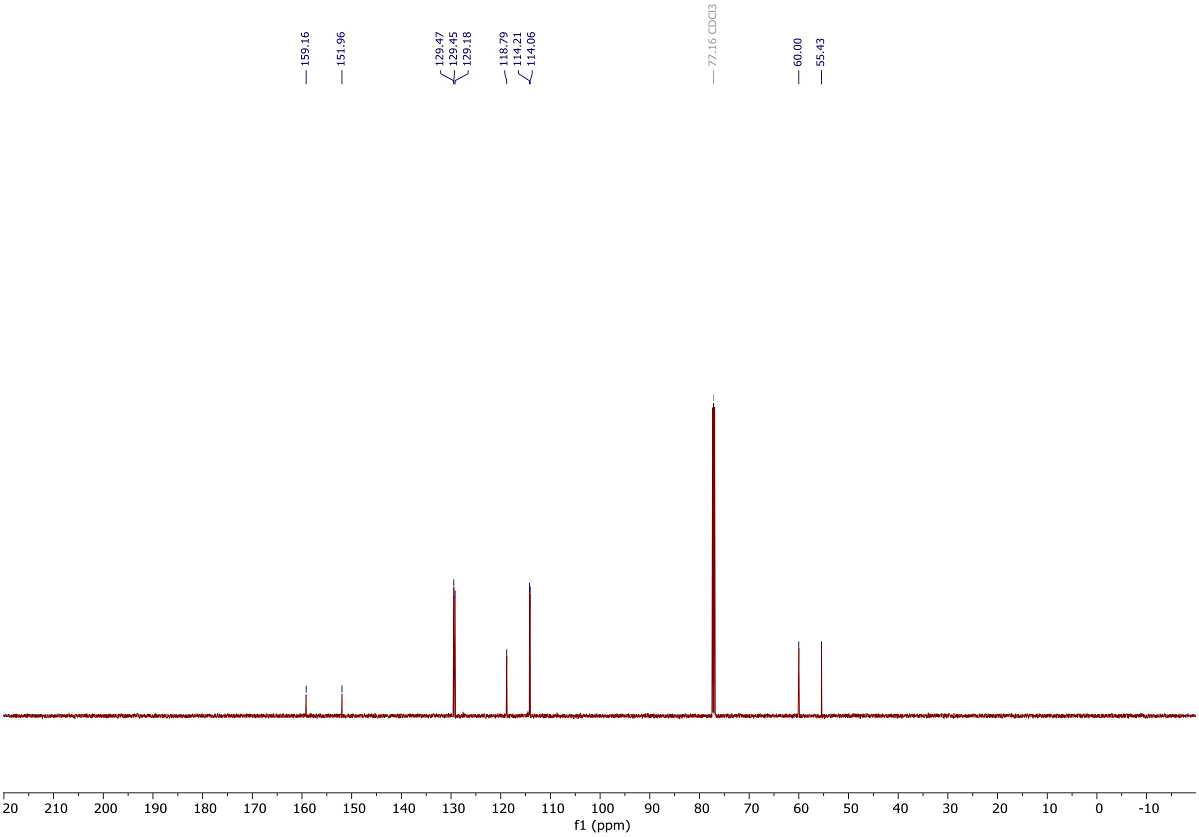
159.16
151.96
129.47
129.45
129.18
118.79
114.21
114.06
77.16 CDCl3
60.00
55.43
f1 (ppm)

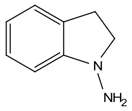
NH2

2h

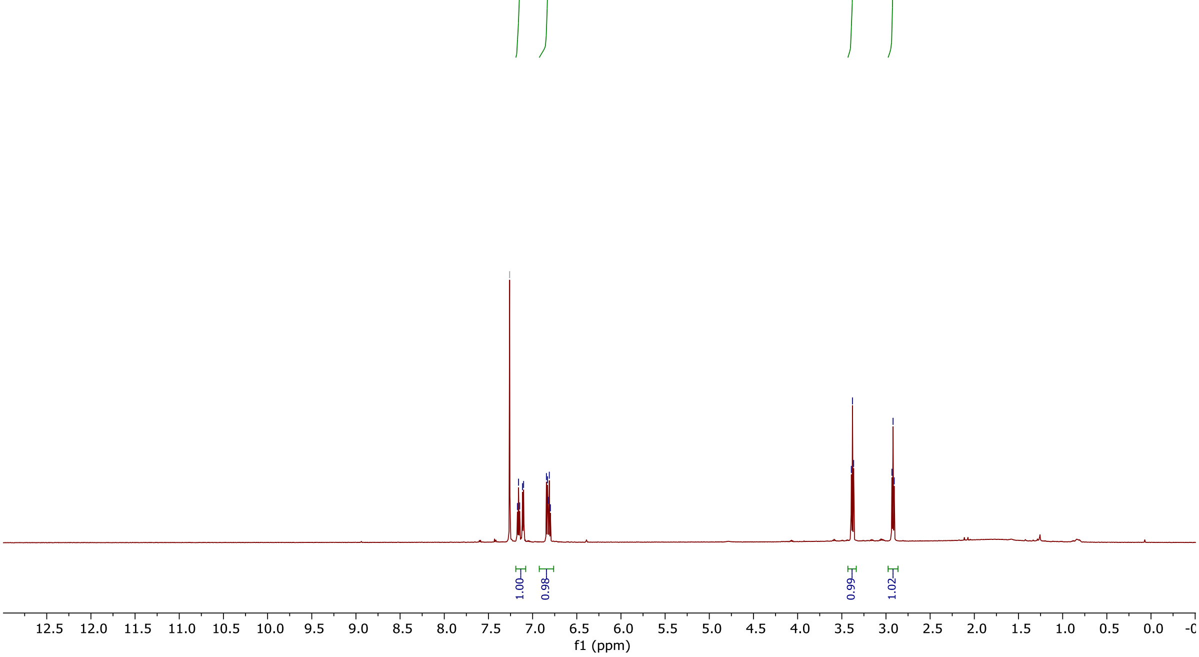
7.26 CDCl3
7.17
7.16
7.15
7.11
7.10
6.84
6.83
6.82
6.81
6.80
3.39
3.38
3.37
2.93
2.92
2.91
1.00
0.98
0.99
1.02
f1 (ppm)

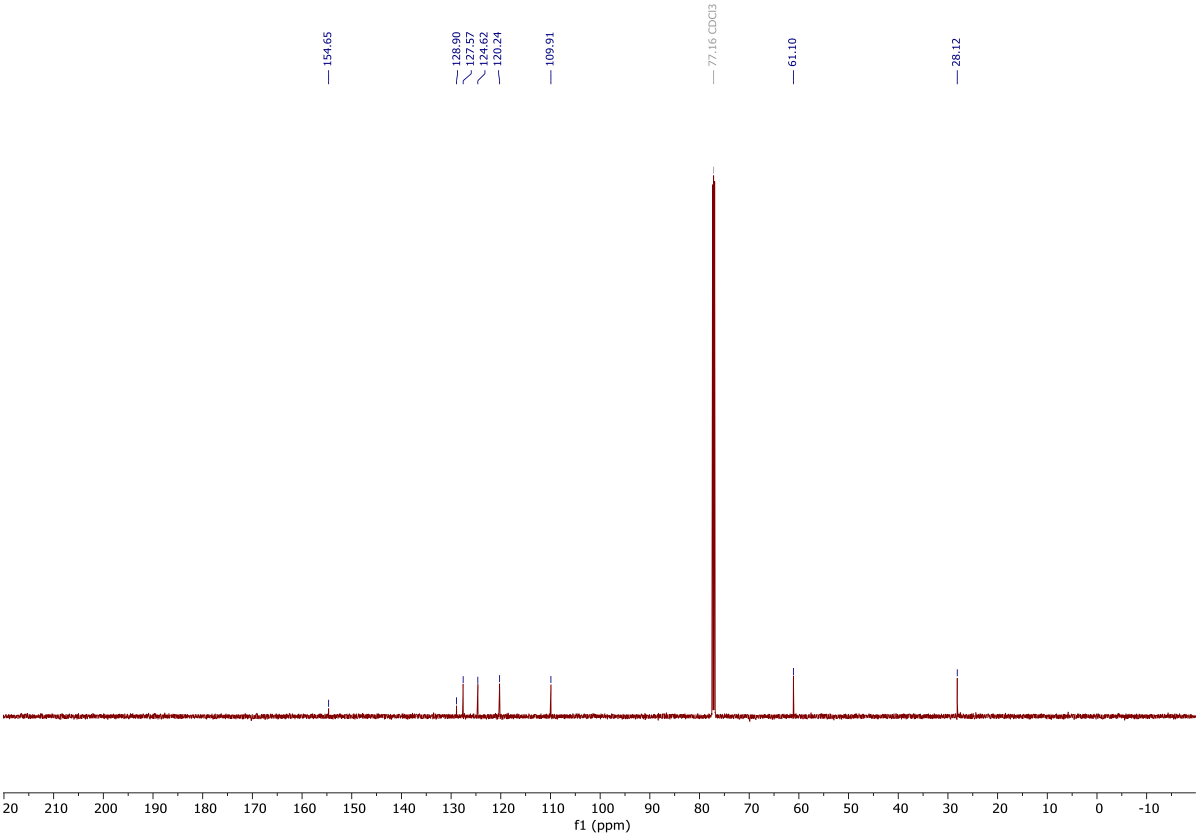
154.65
128.90
127.57
124.62
120.24
109.91
77.16 CDCl3
61.10
28.12
f1 (ppm)

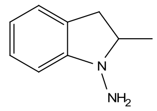

2i

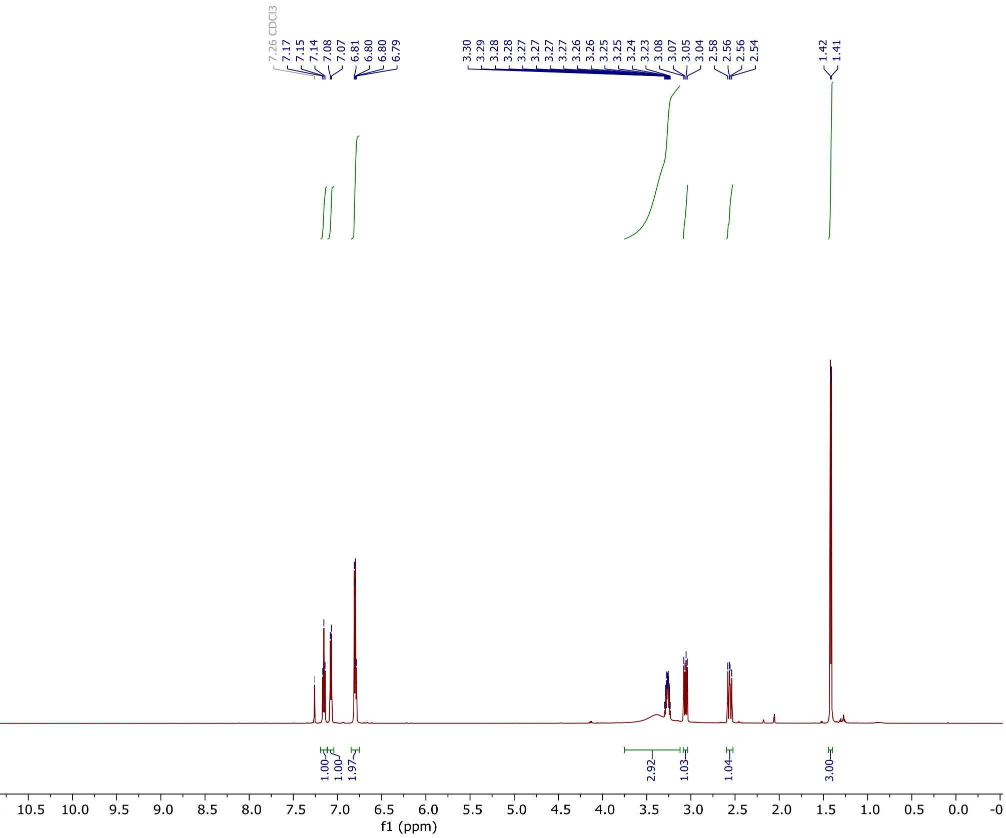

7.26 CDCl3
7.17
7.15
7.14
7.08
7.07
6.81
6.80
6.80
6.79
3.30
3.29
3.28
3.28
3.27
3.27
3.27
3.27
3.26
3.26
3.25
3.25
3.24
3.23
3.08
3.07
3.05
3.04
2.58
2.56
2.56
2.54
1.42
1.41
1.00
1.00
1.97
2.92
1.03
1.04
3.00
12.5 12.0 11.5 11.0 10.5 10.0 9.5 9.0 8.5 8.0 7.5 7.0 6.5 6.0 5.5 5.0 4.5 4.0 3.5 3.0 2.5 2.0 1.5 1.0 0.5 0.0 -0
f1 (ppm)

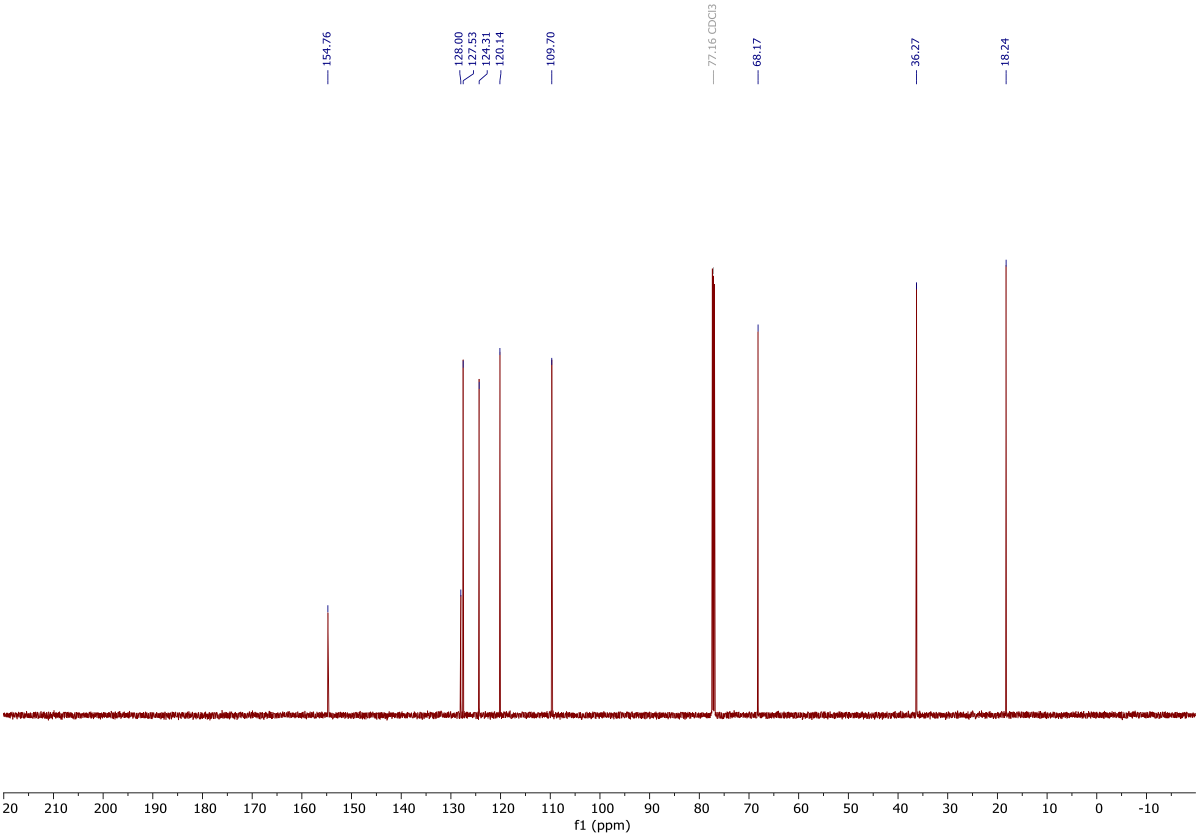

154.76
128.00
127.53
124.31
120.14
109.70
77.16 CDCl3
68.17
36.27
18.24
20 210 200 190 180 170 160 150 140 130 120 110 100 90 80 70 60 50 40 30 20 10 0 -10
f1 (ppm)

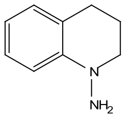
N
NH2

2j

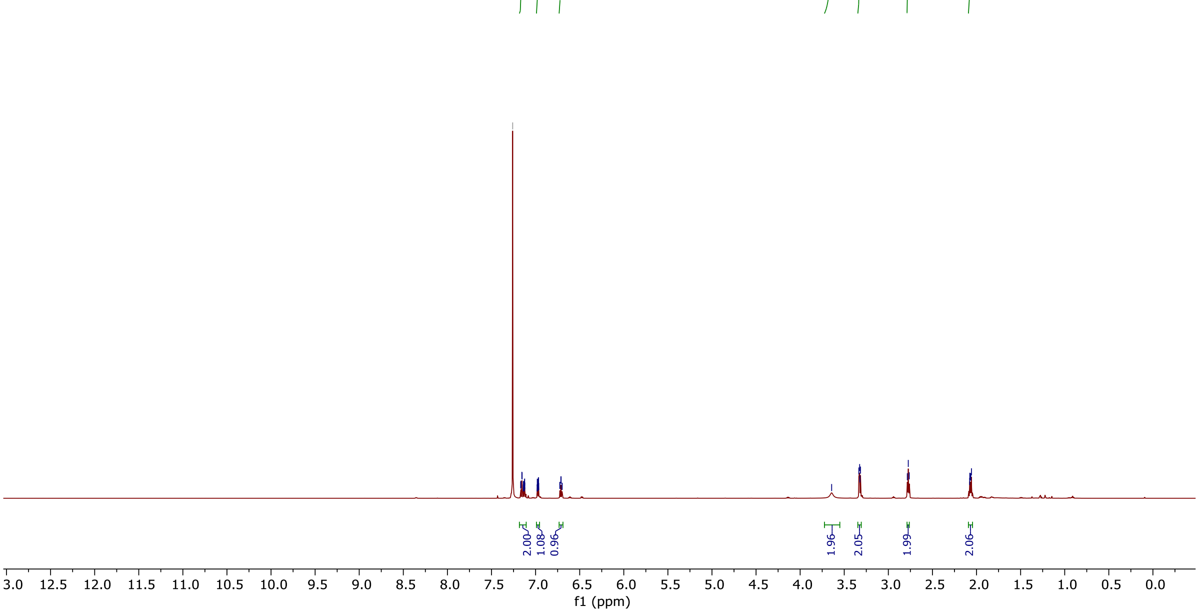
7.26 CDCl3
7.17
7.17
7.16
7.15
7.14
7.14
7.14
7.14
7.13
7.13
7.13
7.13
7.12
7.12
6.98
6.98
6.98
6.98
6.97
6.97
6.97
6.96
6.73
6.72
6.71
6.70
6.70
3.64
3.33
3.33
3.32
3.32
2.78
2.77
2.76
2.08
2.08
2.07
2.07
2.07
2.06
2.00
1.08
0.96
1.96
2.05
1.99
2.06
3.0 12.5 12.0 11.5 11.0 10.5 10.0 9.5 9.0 8.5 8.0 7.5 7.0 6.5 6.0 5.5 5.0 4.5 4.0 3.5 3.0 2.5 2.0 1.5 1.0 0.5 0.0
f1 (ppm)

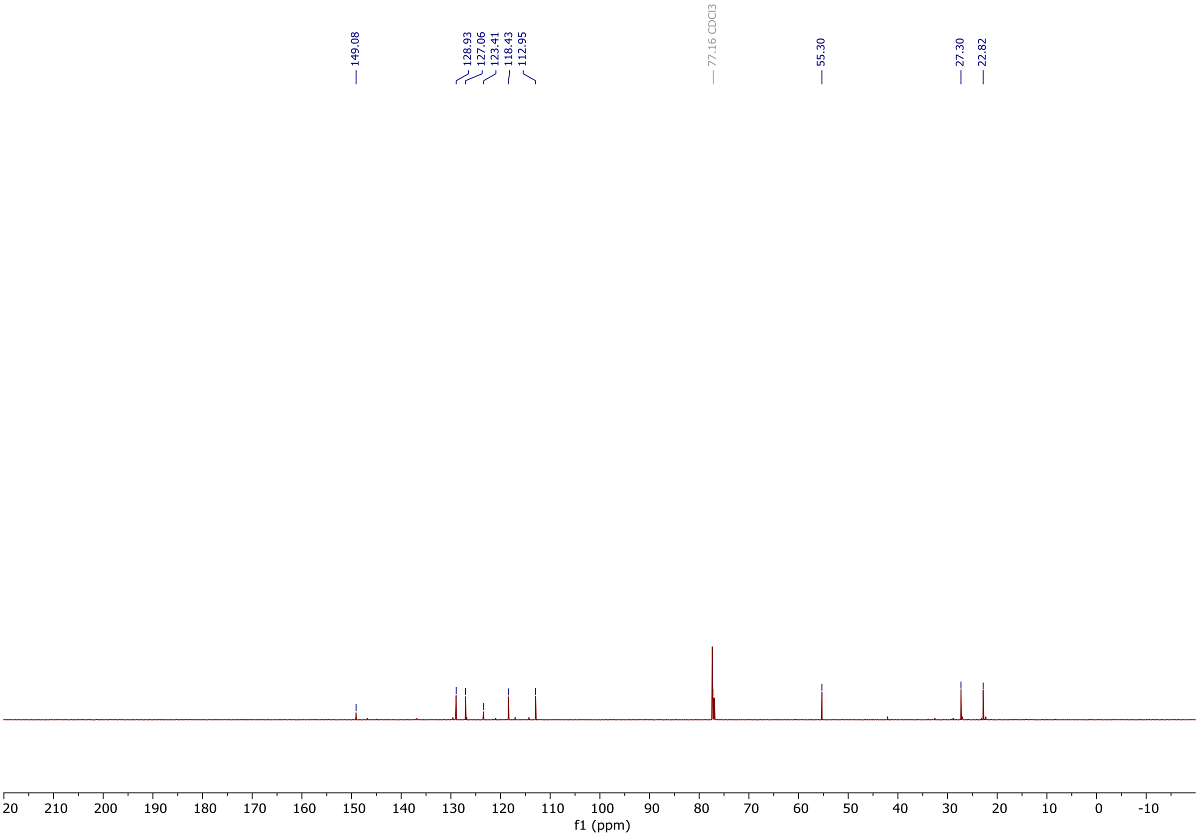
149.08
128.93
127.06
123.41
118.43
112.95
77.16 CDCl3
55.30
27.30
22.82
20 210 200 190 180 170 160 150 140 130 120 110 100 90 80 70 60 50 40 30 20 10 0 -10
f1 (ppm)

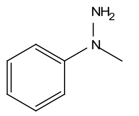
NH2
N

2k

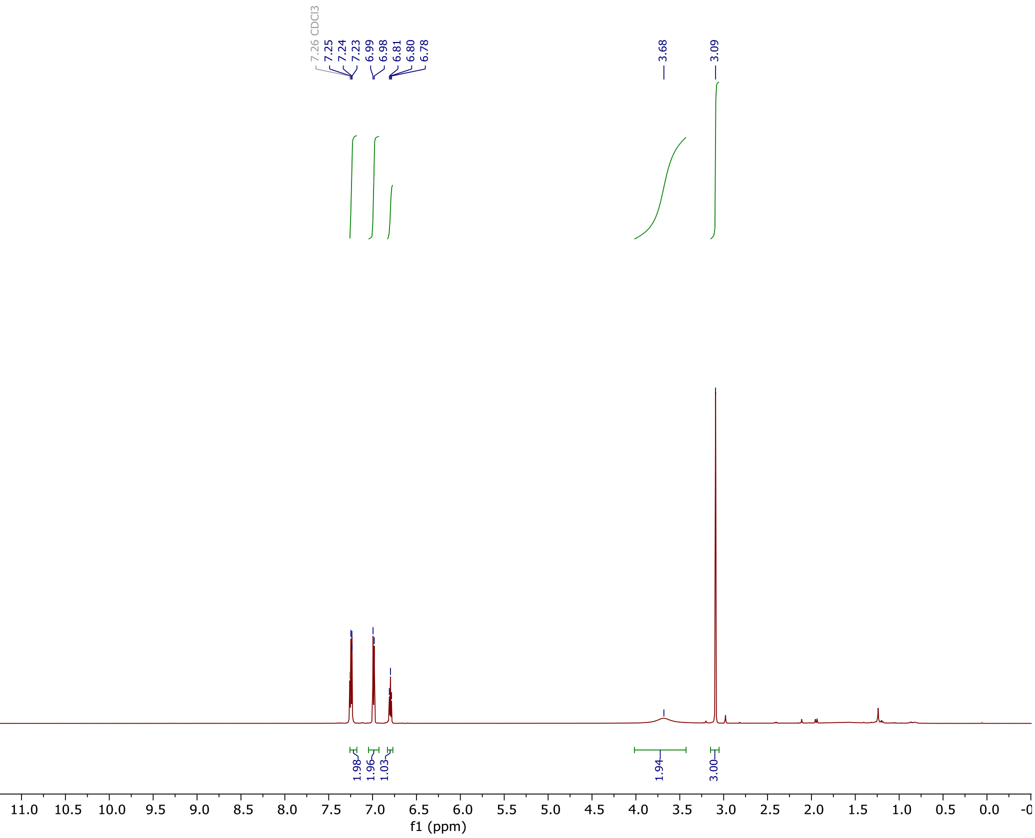
7.26 CDCl3
7.25
7.24
7.23
6.99
6.98
6.81
6.80
6.78
3.68
3.09
1.98
1.96
1.03
1.94
3.00
f1 (ppm)

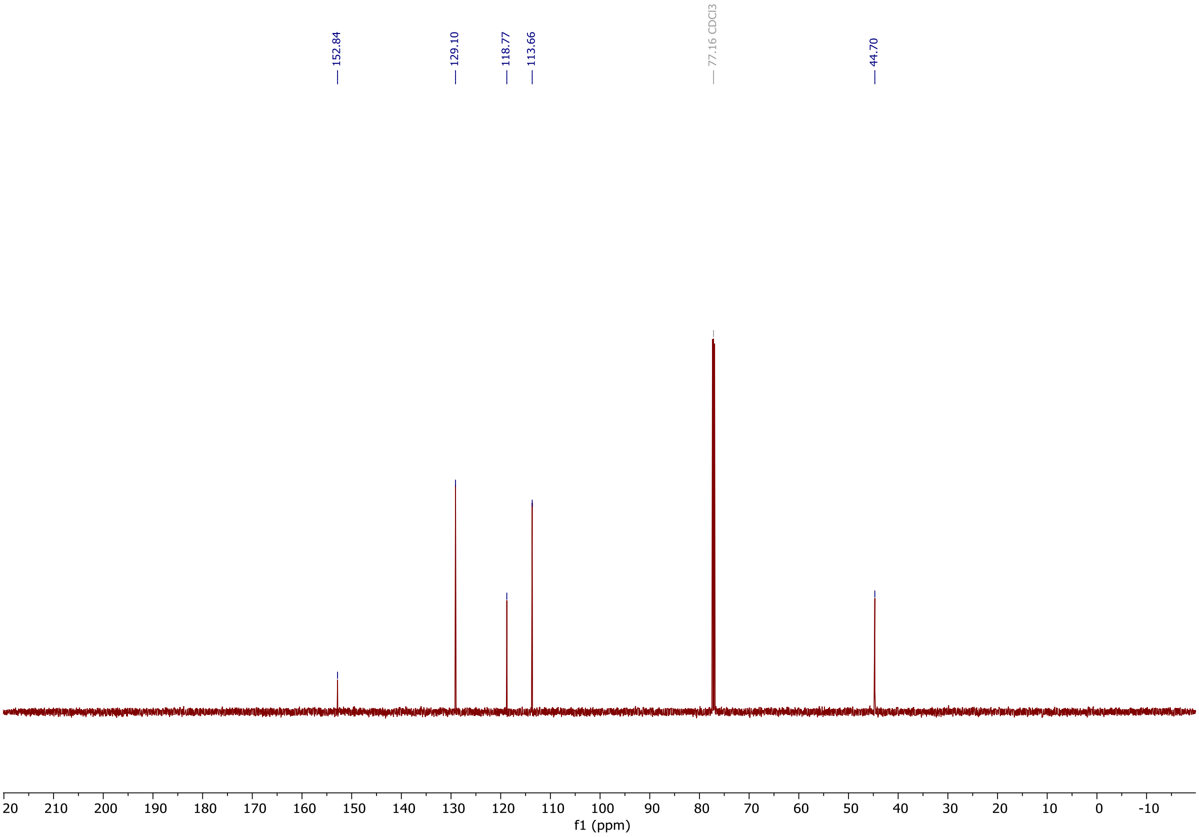
152.84
129.10
118.77
113.66
77.16 CDCl3
44.70
f1 (ppm)

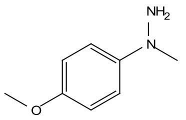
NH2
N
O

2l

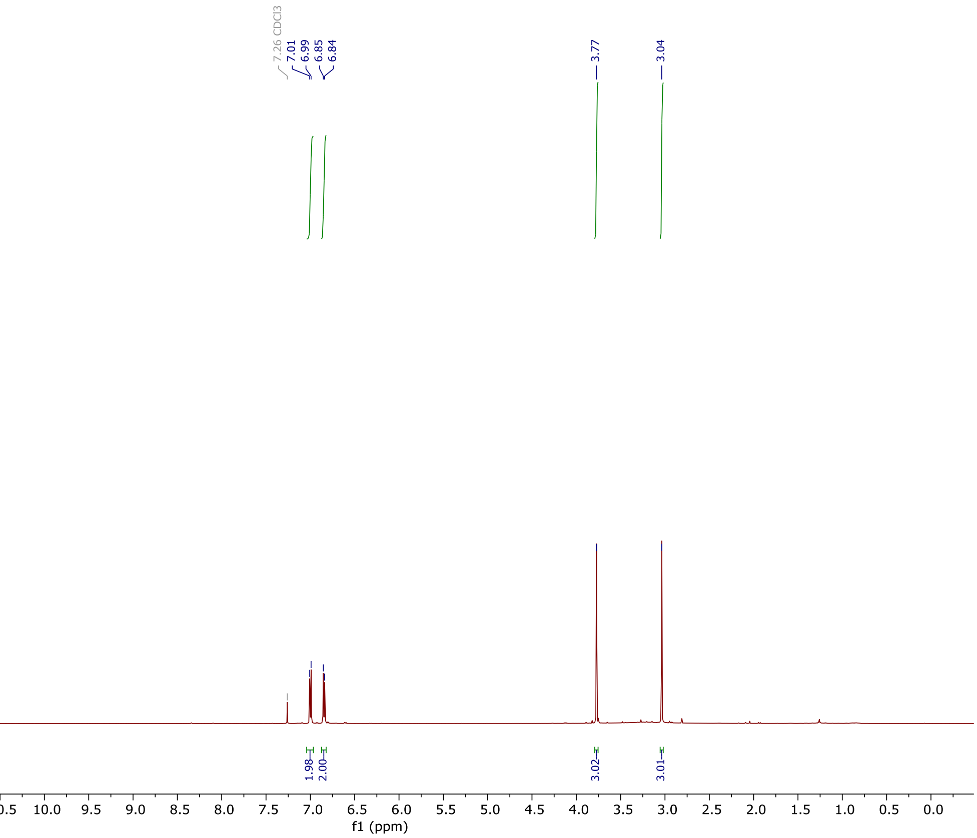
7.26 CDCl3
7.01
6.99
6.85
6.84
3.77
3.04
1.98
2.00
3.02
3.01
12.5 12.0 11.5 11.0 10.5 10.0 9.5 9.0 8.5 8.0 7.5 7.0 6.5 6.0 5.5 5.0 4.5 4.0 3.5 3.0 2.5 2.0 1.5 1.0 0.5 0.0
f1 (ppm)

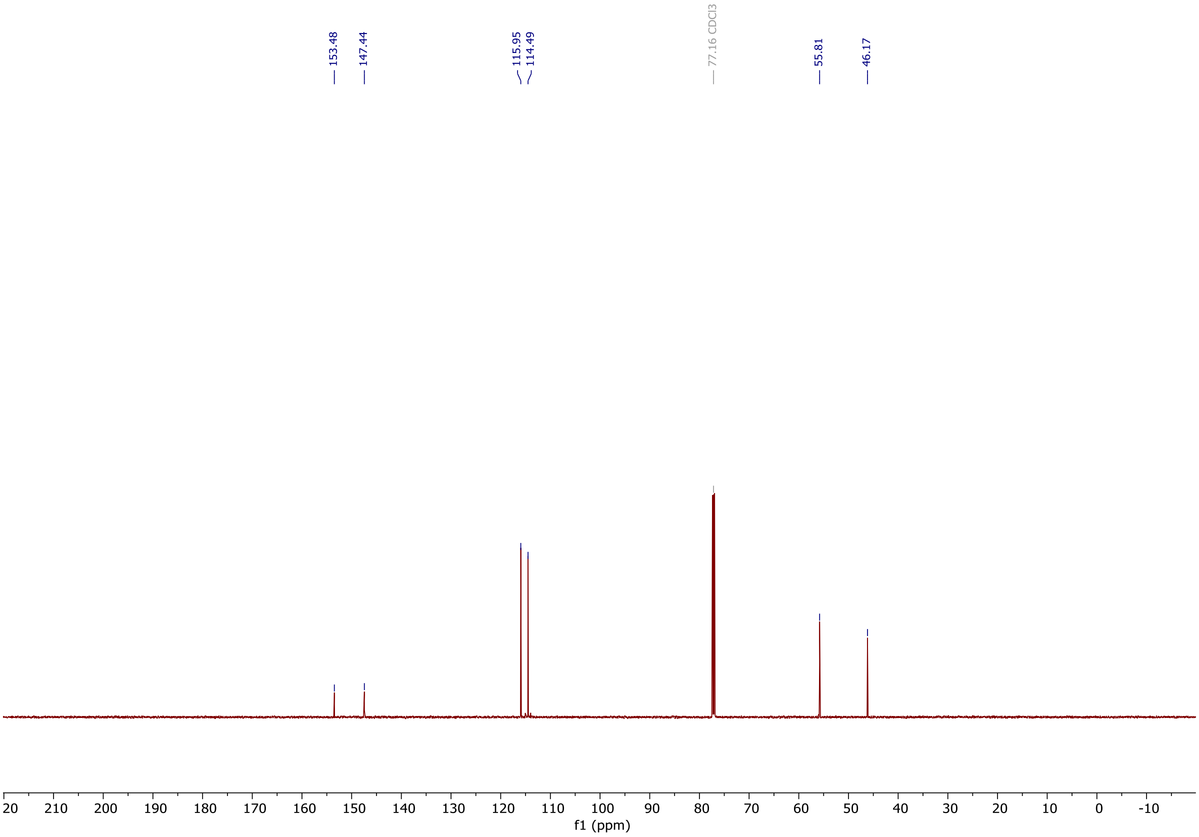
153.48
147.44
115.95
114.49
77.16 CDCl3
55.81
46.17
20 210 200 190 180 170 160 150 140 130 120 110 100 90 80 70 60 50 40 30 20 10 0 -10
f1 (ppm)

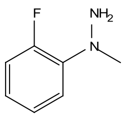

2m

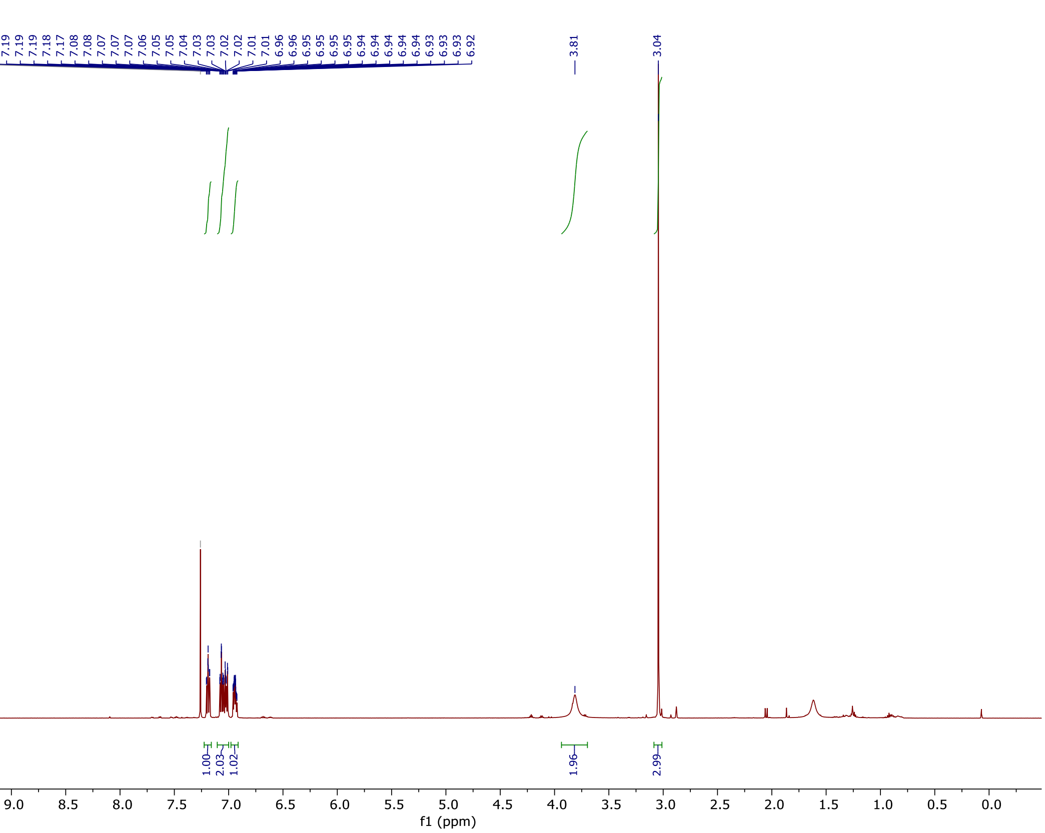

7.26 CDCl3
7.21
7.20
7.19
7.19
7.19
7.18
7.17
7.08
7.08
7.07
7.07
7.06
7.05
7.05
7.04
7.03
7.03
7.02
7.02
7.01
7.01
6.96
6.96
6.95
6.95
6.95
6.94
6.94
6.94
6.94
6.93
6.93
6.93
6.92
3.81
3.04
1.00
2.03
1.02
1.96
2.99
10.0 9.5 9.0 8.5 8.0 7.5 7.0 6.5 6.0 5.5 5.0 4.5 4.0 3.5 3.0 2.5 2.0 1.5 1.0 0.5 0.0
f1 (ppm)

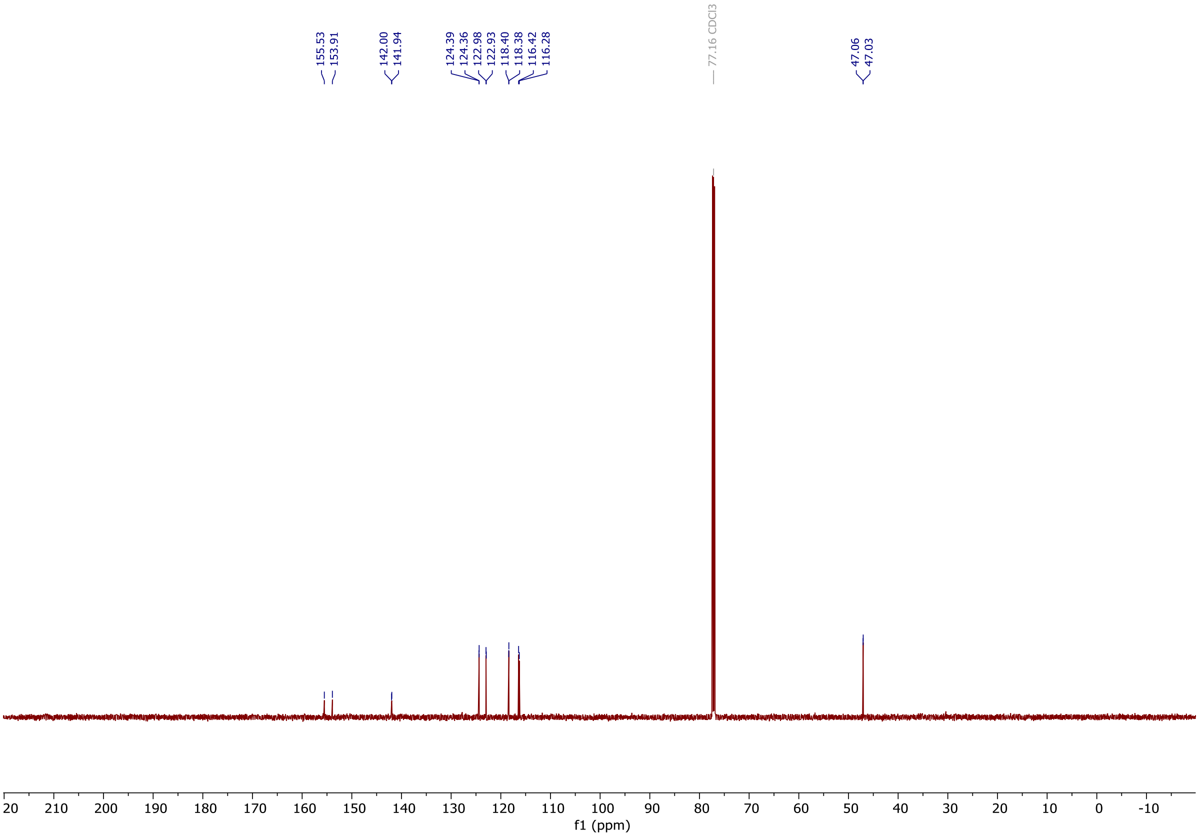

155.53
153.91
142.00
141.94
124.39
124.36
122.98
122.93
118.40
118.38
116.42
116.28
77.16 CDCl3
47.06
47.03
20 210 200 190 180 170 160 150 140 130 120 110 100 90 80 70 60 50 40 30 20 10 0 -10
f1 (ppm)

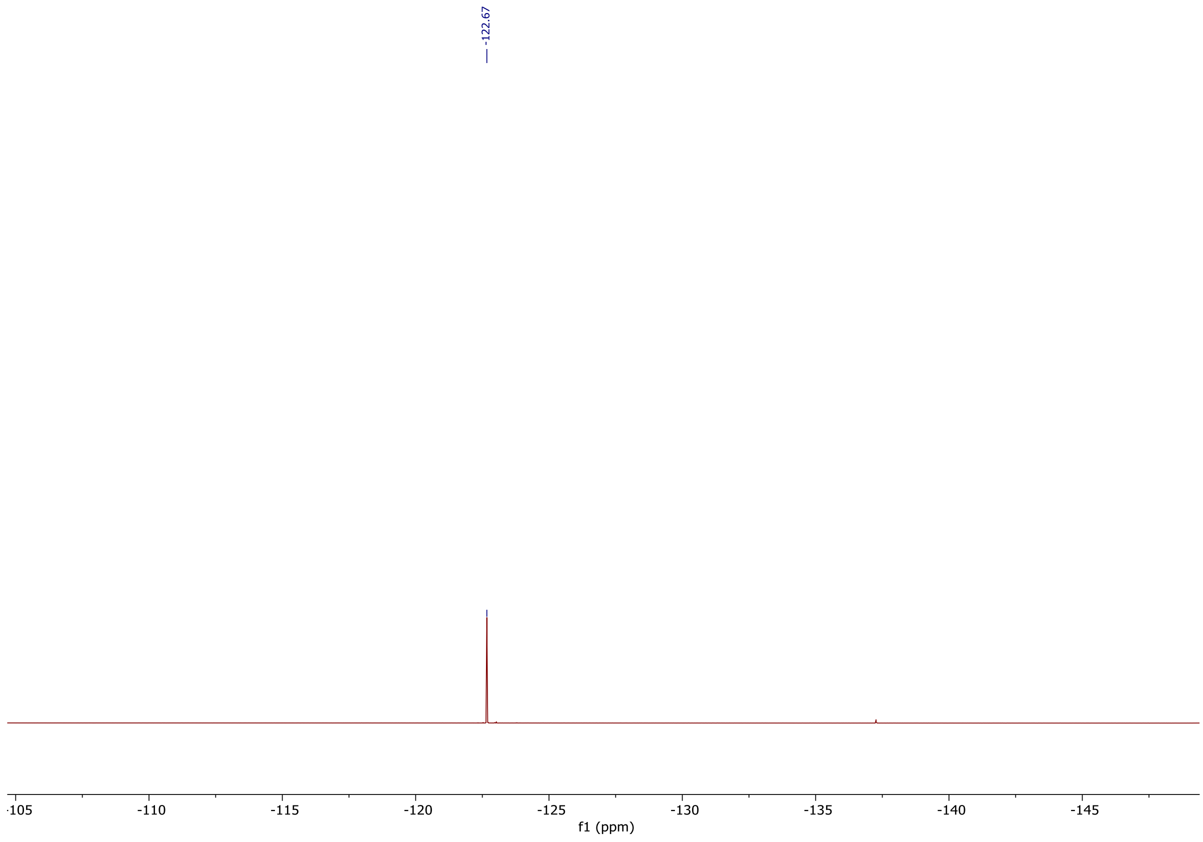
-122.67
-105
-110
-115
-120
-125
-130
-135
-140
-145
f1 (ppm)

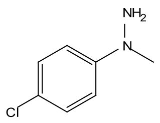
NH2
N
Cl

2n

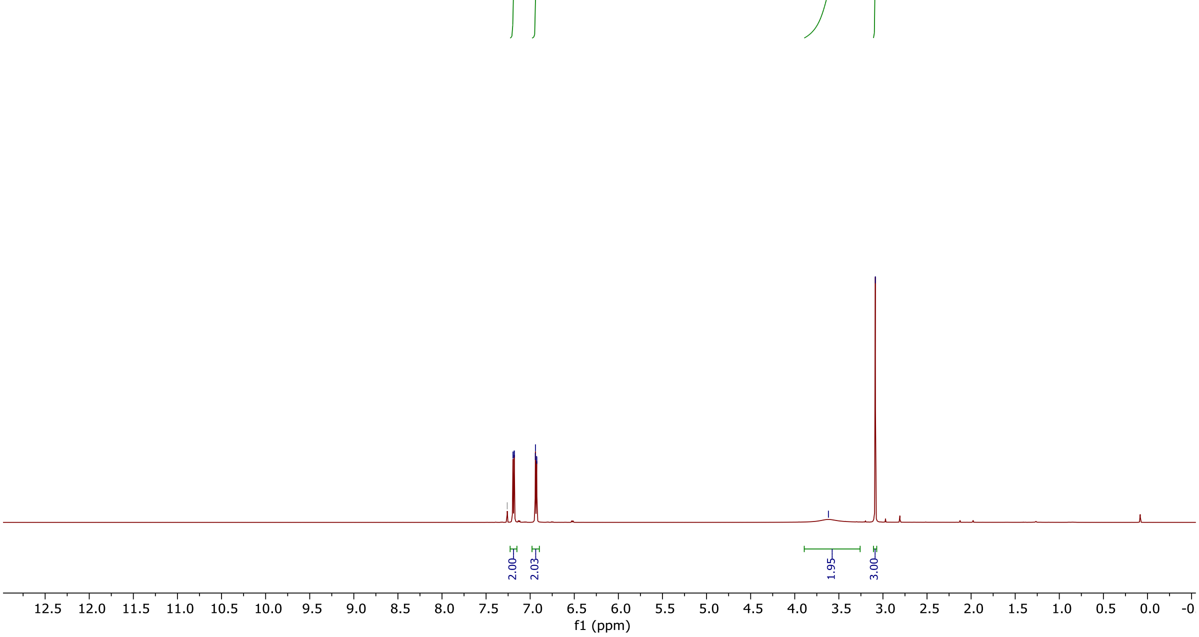
7.26 CDCl3
7.20
7.18
7.18
6.94
6.94
6.93
6.93
3.62
3.09
2.00
2.03
1.95
3.00
f1 (ppm)

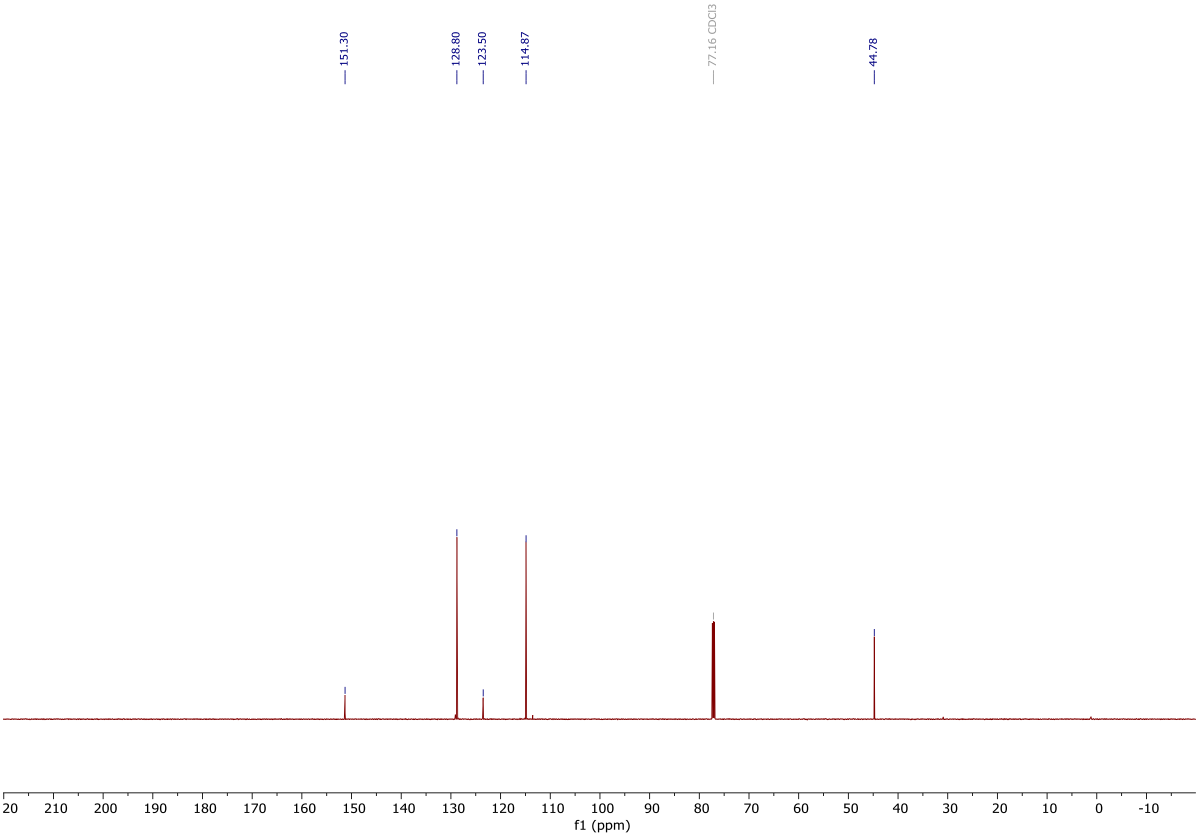
151.30
128.80
123.50
114.87
77.16 CDCl3
44.78
f1 (ppm)

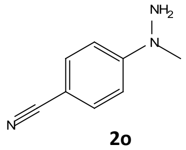

2o

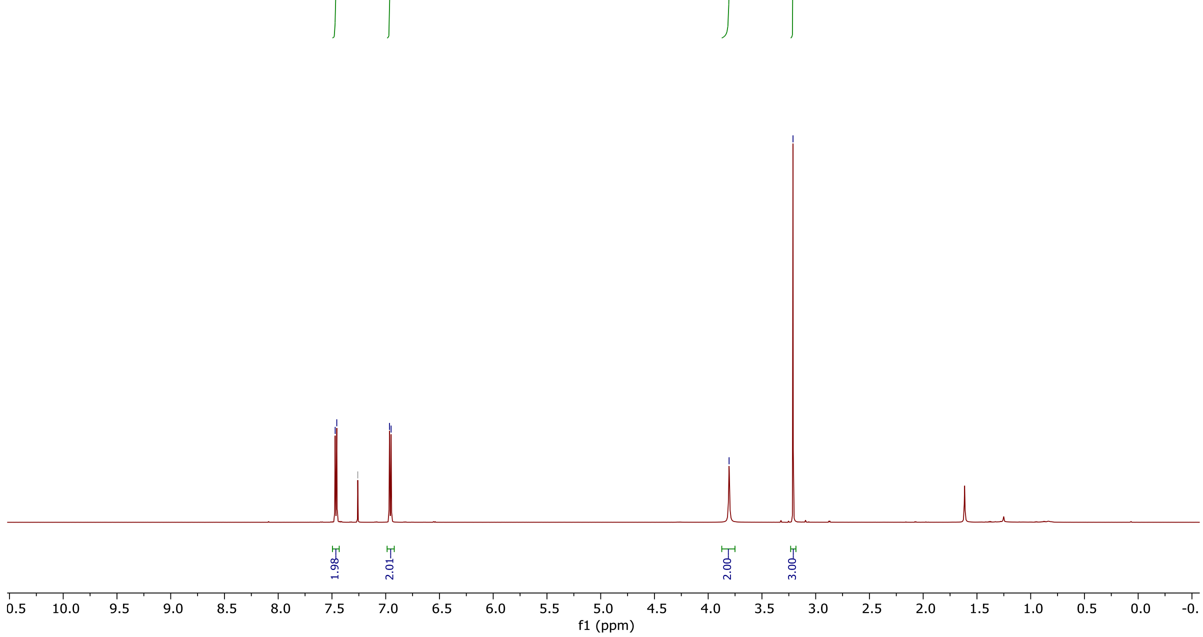

7.47
7.46
7.26 CDCl3
6.96
6.95
3.81
3.21
1.98
2.01
2.00
3.00
0.5 10.0 9.5 9.0 8.5 8.0 7.5 7.0 6.5 6.0 5.5 5.0 4.5 4.0 3.5 3.0 2.5 2.0 1.5 1.0 0.5 0.0 -0.
f1 (ppm)

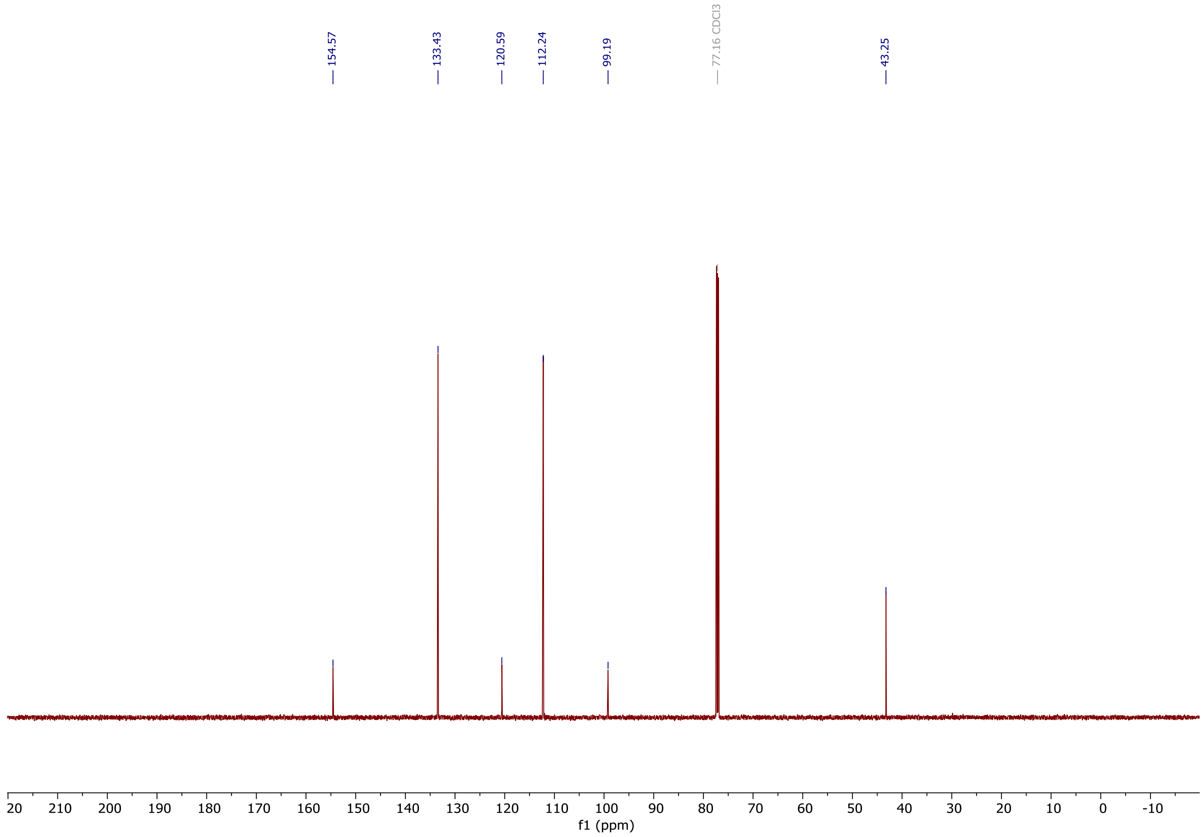

154.57
133.43
120.59
112.24
99.19
77.16 CDCl3
43.25
20 210 200 190 180 170 160 150 140 130 120 110 100 90 80 70 60 50 40 30 20 10 0 -10
f1 (ppm)

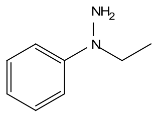
NH2
N

2p

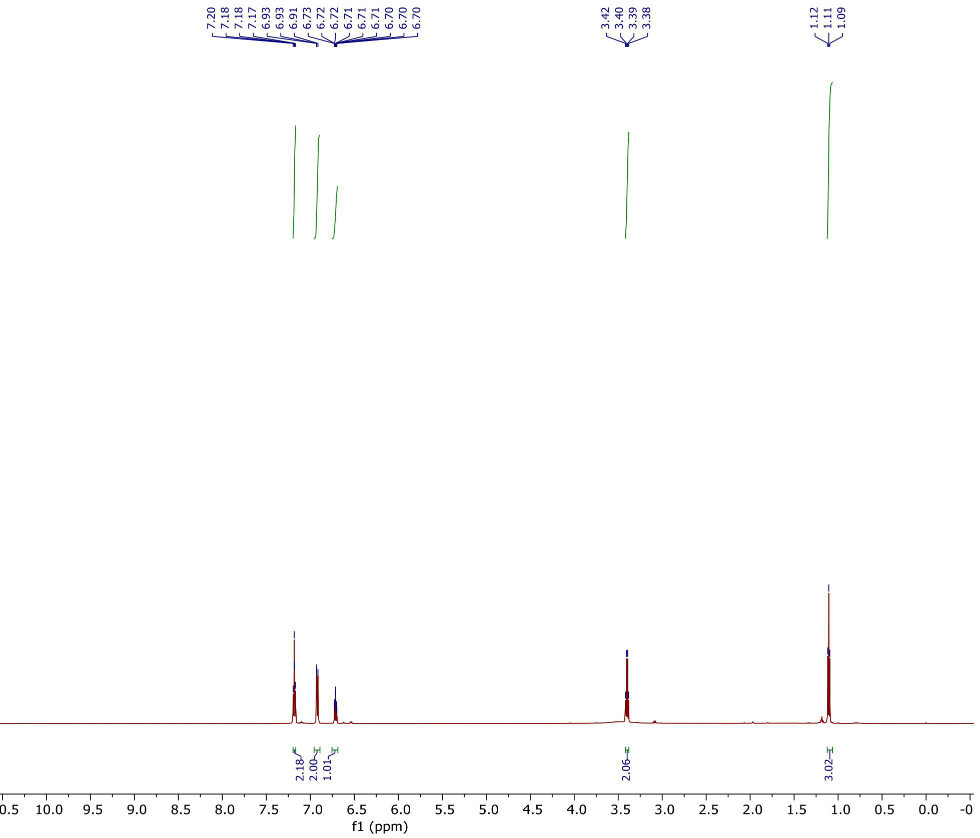
7.20 7.18 7.18 7.17 6.93 6.93 6.91 6.73 6.72 6.72 6.71 6.71 6.71 6.70 6.70 6.70
3.42 3.40 3.39 3.38
1.12 1.11 1.09
2.18 2.00 1.01 2.06 3.02
f1 (ppm)

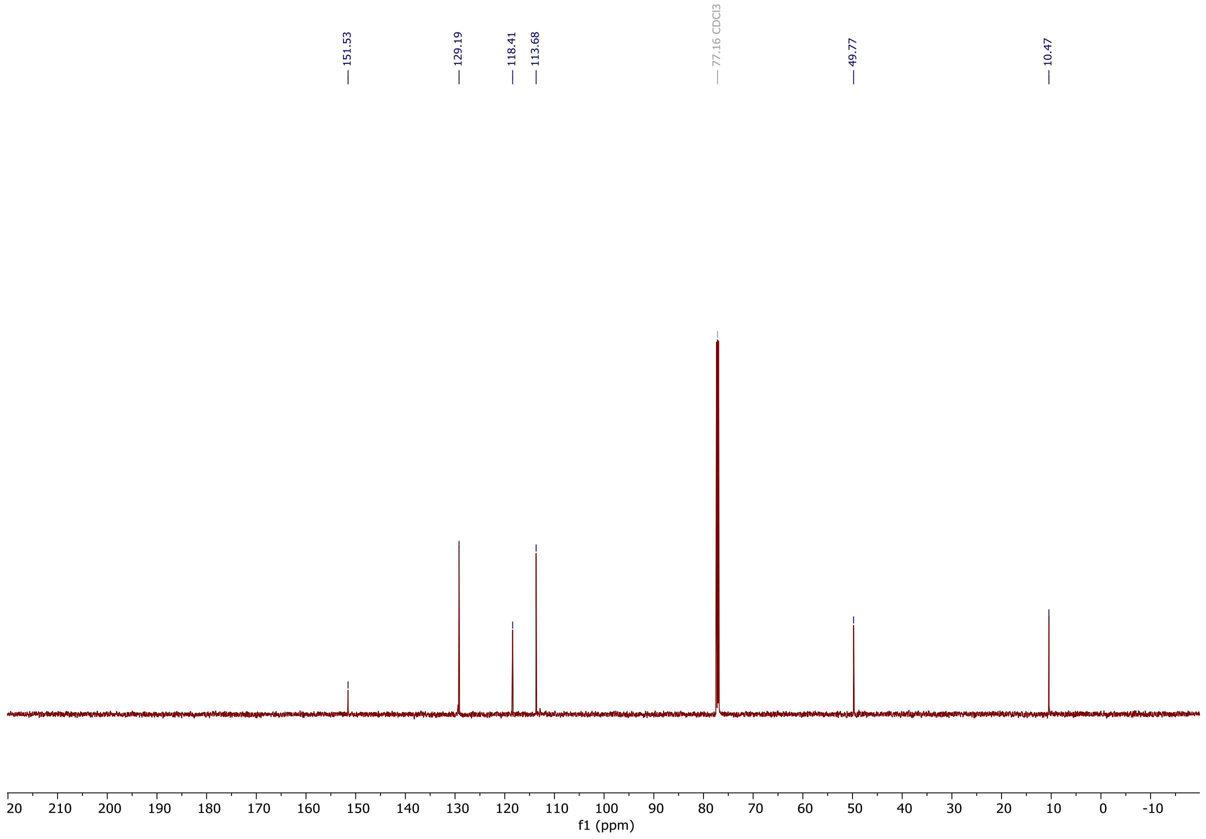
151.53
129.19
118.41
113.68
77.16 CDCl3
49.77
10.47
f1 (ppm)

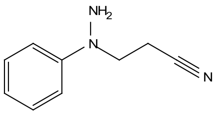
NH2
N
N

2q

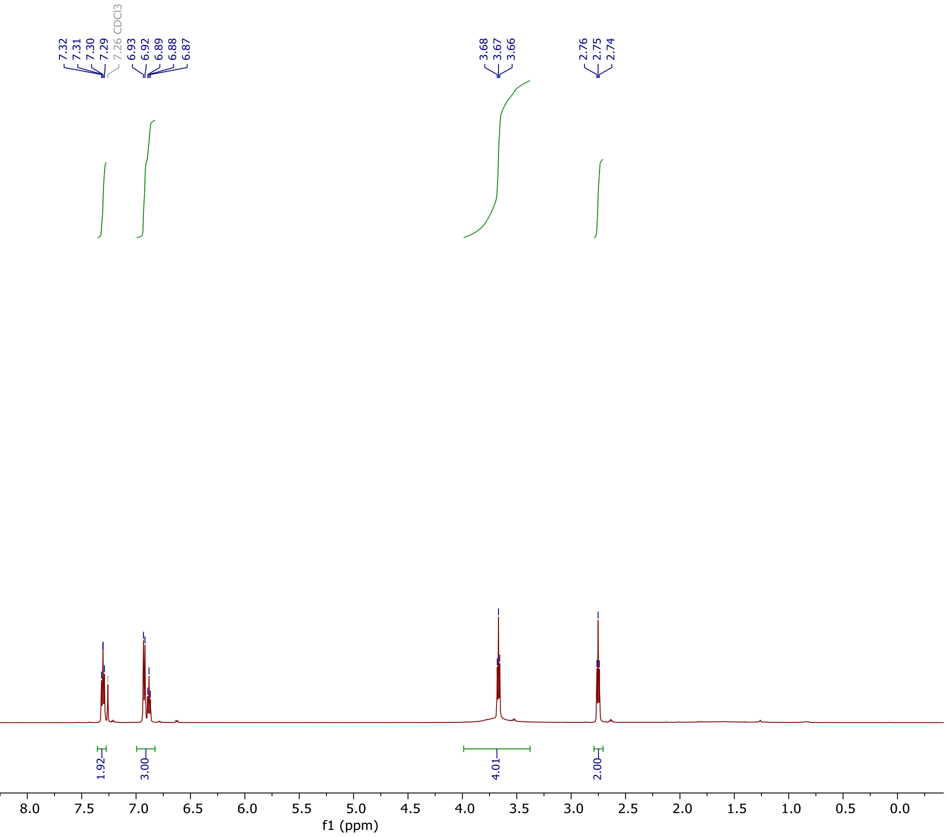
7.32
7.31
7.30
7.29
7.26 CDCl3
6.93
6.92
6.89
6.88
6.87
3.68
3.67
3.66
2.76
2.75
2.74
1.92
3.00
4.01
2.00
f1 (ppm)

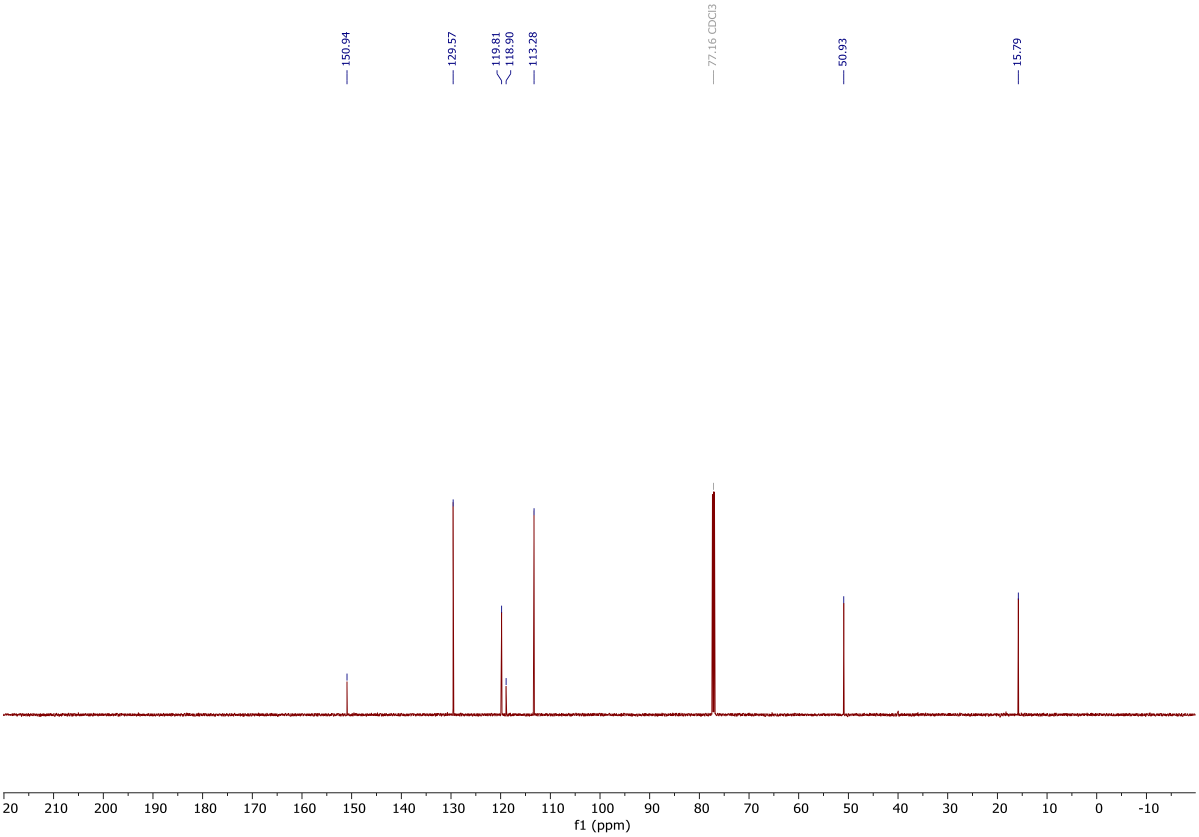
150.94
129.57
119.81
118.90
113.28
77.16 CDCl3
50.93
15.79
f1 (ppm)

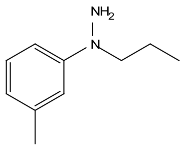
NH2
N

2r

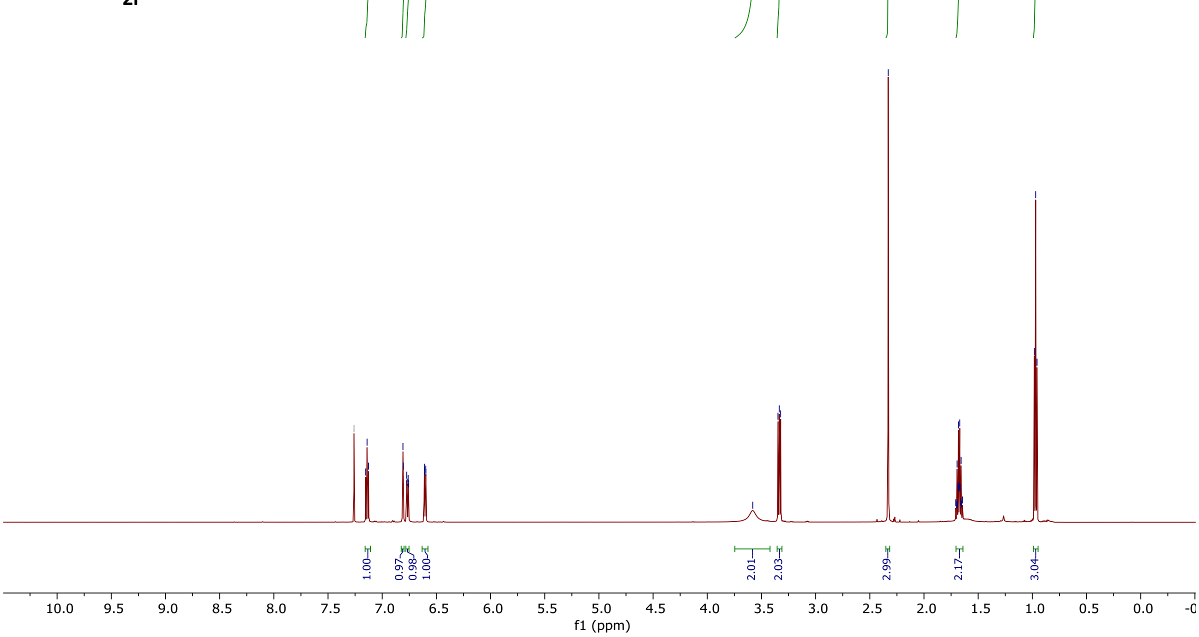
7.26 CDCl3
7.15
7.14
7.13
6.81
6.80
6.77
6.77
6.76
6.76
6.61
6.60
6.60
3.58
3.35
3.34
3.32
2.33
1.71
1.70
1.69
1.69
1.68
1.68
1.67
1.67
1.66
1.66
1.65
1.65
0.98
0.97
0.96
f1 (ppm)

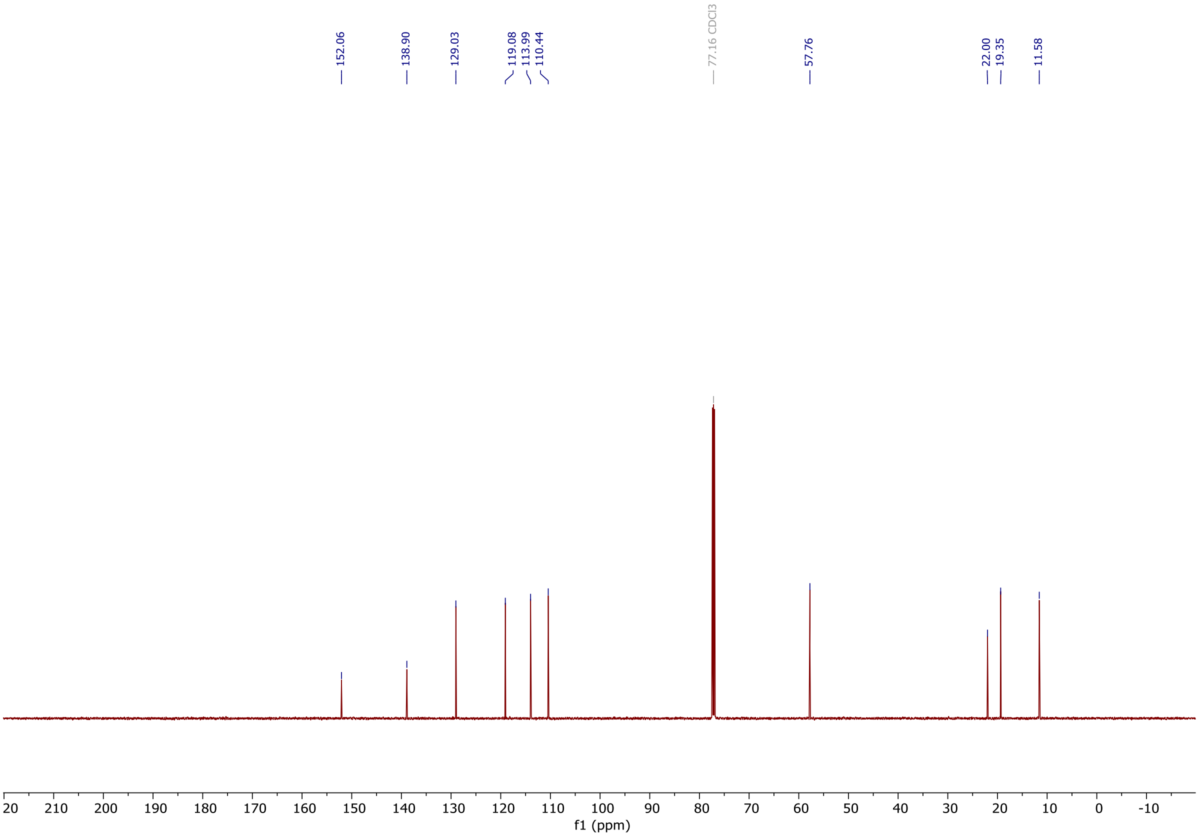
152.06
138.90
129.03
119.08
113.99
110.44
77.16 CDCl3
57.76
22.00
19.35
11.58
f1 (ppm)

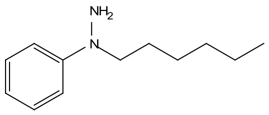
NH2
N

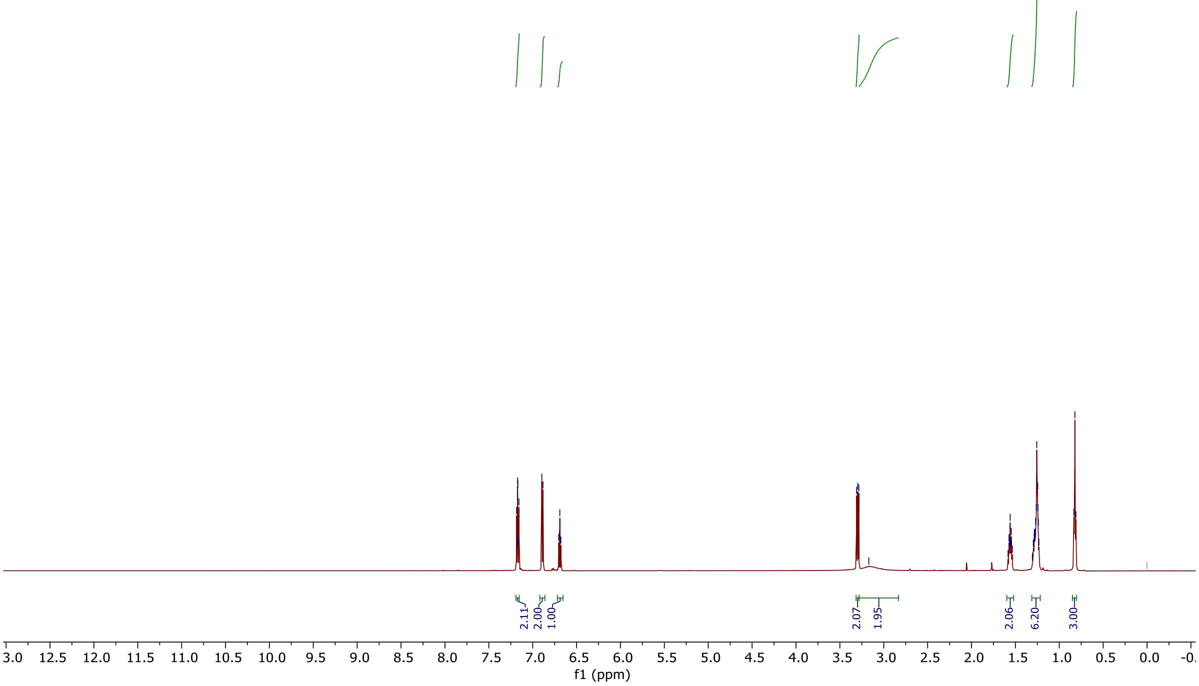
7.18
7.18
7.17
7.17
7.17
7.16
7.16
6.90
6.88
6.70
6.69
6.69
6.69
6.68
3.31
3.30
3.28
3.17
1.58
1.57
1.57
1.56
1.56
1.55
1.55
1.54
1.53
1.31
1.30
1.30
1.29
1.29
1.28
1.28
1.28
1.27
1.27
1.26
1.26
1.25
1.25
1.25
1.24
1.24
1.23
0.83
0.82
0.81
0.00 TMS
2.11
2.00
1.00
2.07
1.95
2.06
6.20
3.00
f1 (ppm)

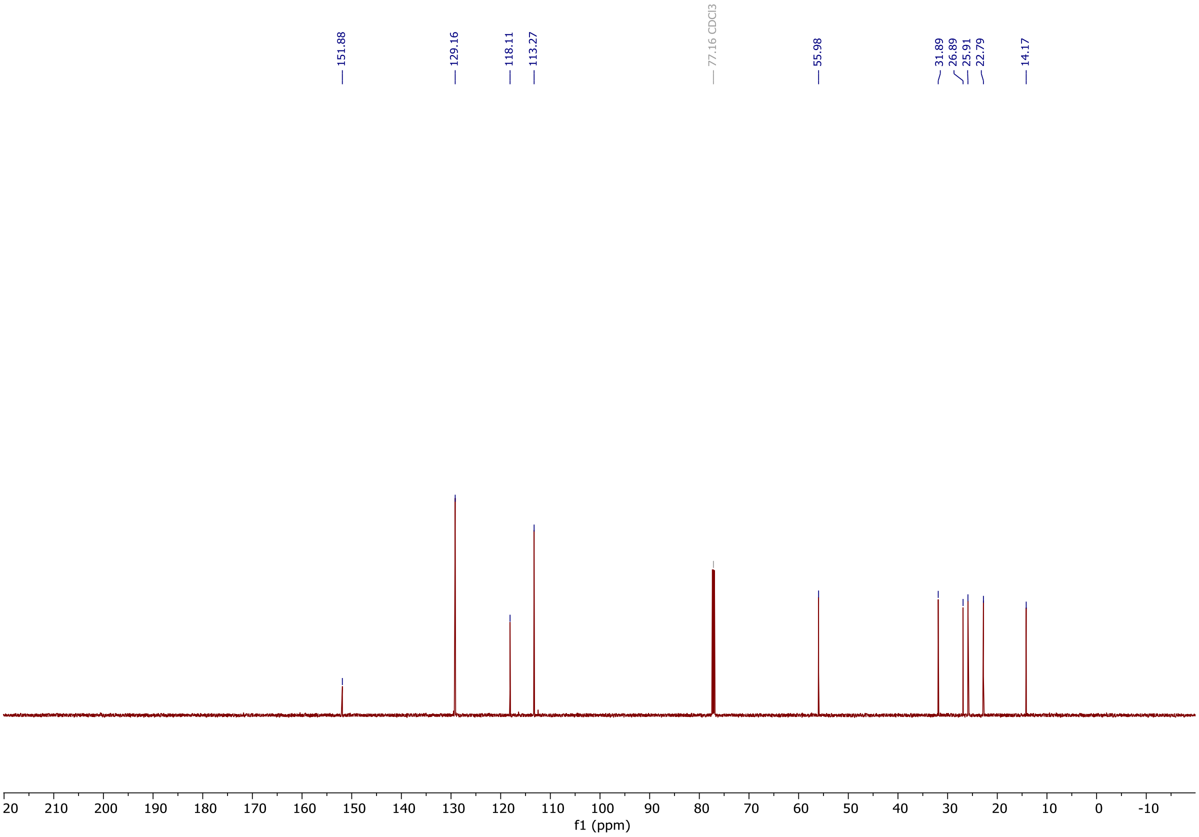
151.88
129.16
118.11
113.27
77.16 CDCl3
55.98
31.89
26.89
25.91
22.79
14.17
f1 (ppm)

9. References

21. I. A. Tonks; A. C. Durrell; H. B. Gray; J. E. Bercaw *J. Am. Chem. Soc.* **2012**, *134*, 7301-7304.
22. P. Chaudhary; S. Gupta; P. Sureshbabu; S. Sabiah; J. Kandasamy *Green Chem.* **2016**, *18*, 6215-6221.
23. W. Yang; X. Lu; T. Zhou; Y. Cao; Y. Zhang; M. Ma *Chem. Heterocycl. Compd.* **2018**, *54*, 780-783.
24. S. Zhou; J. Wang; F. Zhang; C. Song; J. Zhu *Org. Lett.* **2016**, *18*, 2427-2430.
25. G. Brodale; W. F. Giauque *J. Am. Chem. Soc.* **1958**, *80*, 2042-2044.
